# Chronic airway-induced allergy in mice modifies gene expression in the brain toward insulin resistance and inflammatory responses

**DOI:** 10.1186/1742-2094-10-99

**Published:** 2013-08-01

**Authors:** Heela Sarlus, Xiuzhe Wang, Angel Cedazo-Minguez, Marianne Schultzberg, Mircea Oprica

**Affiliations:** 1 Karolinska Institutet, Department of Neurobiology, Care Sciences & Society, Division of Neurodegeneration, Novum, Floor 5, SE-141 86, Stockholm, Sweden; 2 Karolinska Institutet, Department of Neurobiology, Care Sciences & Society, Division of Alzheimer’s Disease Research Center, Novum, Floor 5, SE-141 86, Stockholm, Sweden

**Keywords:** Allergy, Insulin-degrading enzyme, Insulin signaling, Neurodegeneration, Neuroinflammation

## Abstract

**Background:**

Chronic systemic inflammation affects brain functionality and may negatively influence the progression of neurodegenerative disorders. Allergy is a chronic inflammatory disease affecting more than 20% of the Western population. Little is known regarding the influence of allergy on brain functions. The aim of the present study was to obtain a global overview of the genes that drive the effects of peripheral inflammation associated with chronic airway-induced allergy in the brain.

**Methods:**

Airway allergy was induced in C57B/6J mice using ovalbumin as the allergen. Microarray analysis was performed in the hippocampus and frontal cortex in association with Affymetrix. For the data analysis, principal component analysis and orthogonal to latent structures discriminant analysis followed by pathway analysis were used. Quantitative polymerase chain reaction (qPCR) and protein analysis by Western blotting were performed for the validation of microarray results.

**Results:**

Microarray analysis showed low-grade changes in gene expression in the brain induced by airway-associated allergy. Changes in expression were observed for genes involved in antigen processing and presentation, cytokine–cytokine interaction, Toll-like receptor and mitogen-activated protein kinase signaling, as determined by pathway analysis. We confirmed a reduction of insulin-degrading enzyme at the protein level and a decrease in insulin receptor phosphorylation in the brains of allergic mice. Other allergy-induced gene expression changes were confirmed by qPCR, including increased levels of tumor necrosis factor receptor superfamily member 23 and lipopolysaccharide-binding protein.

**Conclusion:**

Airway-associated allergy induces changes in brain gene expression toward induction of insulin resistance and inflammatory responses with potential implications for neurodegenerative disorders.

## Background

Systemic inflammation followed by increased levels of brain proinflammatory cytokines and adaptive behavioral changes constitute a classic example of immune body-to-brain communication that occurs during acute infections and is known as “sickness behavior” [1,2]. However, the effects of chronic peripheral inflammation on the brain have not been studied extensively. Recent data show that chronic systemic inflammation is associated with structural brain changes. White and gray matter atrophy has been observed in the brains of patients with rheumatoid arthritis [3,4] and systemic lupus erythematosus [3,4].

It is known that inflammatory processes occur in the brain in most neurodegenerative disorders. Furthermore, systemic inflammation has been shown to exacerbate the ongoing neurodegenerative processes in the brain in neurodegenerative disorders such as multiple sclerosis, Parkinson disease, prion disease and cerebral ischemia [5,6]. Thus, studies of the influence of chronic peripheral inflammation on the brain are of particular significance, mainly for brain diseases with underlying neurodegenerative pathology.

Asthma, allergic rhinitis and atopic dermatitis are among the most commonly encountered diseases with chronic allergy, known as atopic disorders, often with onset occurring during childhood or adolescence. Asthma is a chronic systemic inflammatory disorder of the airways that affects about 300 million people worldwide [7]. It is characterized by increased levels of cytokines, infiltration of eosinophils and T-helper type 2 (Th2) cells into the airway submucosa, reversible airway obstruction, airway hyperresponsiveness and airway remodeling [8].

Studies with functional brain magnetic resonance imaging in allergic patients have shown increased activity in the brain, mainly in the anterior insular cortex (AIC) and anterior cingulate cortex [9]. The increased AIC activity was correlated with the degree of inflammation in the lungs, as well as with disease severity [10]. These findings indicate that allergy associated with asthma influences neuronal circuits involved in the processing of emotional information.

Allergy is characterized by an anti-inflammatory Th2 profile, suggesting that allergic diseases may be associated with an inflammatory phenotype, which at first glance may prove beneficial for diseases characterized by a proinflammatory Th1 profile such as Alzheimer disease (AD). However, studies in mouse models of allergy have shown effects of inflammation associated with allergy on brain function. Thus, mice challenged with ovalbumin (OVA) had increased expression of the immediate early gene *c-fos* in different brain regions [11]. Increased brain levels of cytokines such as interleukin 1 (IL-1) and tumor necrosis factor α (TNF-α) were found in mice exposed to OVA and particulate matter [12]. In a recent study, using a chronic airway allergy model, we showed increased levels of immunoglobulins (Igs) in the brains of allergic mice [13]. Furthermore, an epidemiological study showed a positive correlation between a history of allergic diseases and risk for dementia [14].

The aim of the present study was to obtain a wider perspective on gene expression in the brain in response to allergy, which may lead to the finding of potential connections with diseases, or groups of diseases, including neurodegenerative disorders.

## Methods

### Animals and assays

#### Animals

Male mice 12 to 14 weeks old C57B6 (20 to 22 g, *n* = 16) were purchased from B&K Universal AB (Sollentuna, Sweden). The animals were housed four per cage under controlled conditions of light–dark cycle (12:12 h, lights on at 06:00 h), temperature (21 ± 1°C), relative humidity (60% to 65%) and food and water *ad libitum*. Upon arrival, the animals were habituated to the environment for 2 wk before the start of experiments and handled daily to minimize the stress level after the start of the chronic allergy protocol. The study was approved by the Stockholm South local committee on ethics of animal experiments (S204/10).

#### Allergen exposure protocol

Both AD and allergy are chronic disorders, and we have previously validated a chronic model of airway-induced allergy using a chronic OVA challenge protocol [13]. Briefly, the mice were sensitized with a single intraperitoneal (i.p.) injection of a 200-μl suspension of Al(OH)_3_ (4 mg/ml) in phosphate-buffered saline (PBS) containing OVA grade III (0.05 mg/ml; Sigma-Aldrich, St Louis, MO, USA) (OVA-alum) on days 0 and 12. The animals were then challenged daily from day 18 to day 23, and then three times per week during an additional 5-wk period, by intranasal instillation of 50 μl of an OVA-alum suspension containing 2 mg/ml OVA. Control animals received PBS instead of OVA but otherwise underwent the same treatment.

The animals were killed 24 h after the last antigen challenge, and the hippocampus, frontal cortex and hypothalamus were quickly dissected out, frozen on dry ice and stored at −70°C until processed for gene expression and biochemical studies, including microarrays, RT-PCR and immunoblot analysis.

### Microarray technology

#### Tissue processing

Total RNA was extracted from the left frontal cortex and hippocampus (approximately 60 mg and 20 mg of tissue, respectively), using the QIAzol lysis reagent buffer and purified using the RNeasy Mini kit according to the manufacturer’s instructions (QIAGEN Sciences, Germantown, MD, USA). The right frontal cortex and hippocampus were processed for Western blotting as described below.

Microarray analysis was performed using Affymetrix whole-transcript expression analysis and the Mouse Gene 1.1^ST^ profiling array (Affymetrix, Santa Clara, CA, USA) in association with the Bioinformatics and Expression Analysis Core Facility (BEA), Karolinska Institutet. The array plate contains more than 770,000 oligonucleotide probes (25-mere) that cross-examine more than 28,000 genes.

### Microarray data analysis

The microarray data processing is summarized into four main steps: (1) quality control, (2) data preprocessing, (3) detection of differentially expressed genes (DEGs) and (4) extraction of biological knowledge [15]. After we performed the quality control, we used the Probe Logarithmic Intensity Error (PLIER) algorithm to obtain the expression level of the gene with a cutoff value of 30 units for the expression above background in all samples. Genes with expression below the background were regarded as not expressed and therefore were filtered out of the analysis. When the proportion of differentially expressed transcripts in the dataset is small, they tend to get buried among the nondifferentially expressed transcripts. There are several alternatives suggested for filtering of genes [15]. Thus, to reduce the noise, genes were filtered by fold change using cutoff values of ±1.10 and ±1.15 for hippocampus and frontal cortex, respectively. This reduced the number of genes from approximately 14,000 to 1,488 in the hippocampus and 1,459 in the frontal cortex.

**Figure 1:**
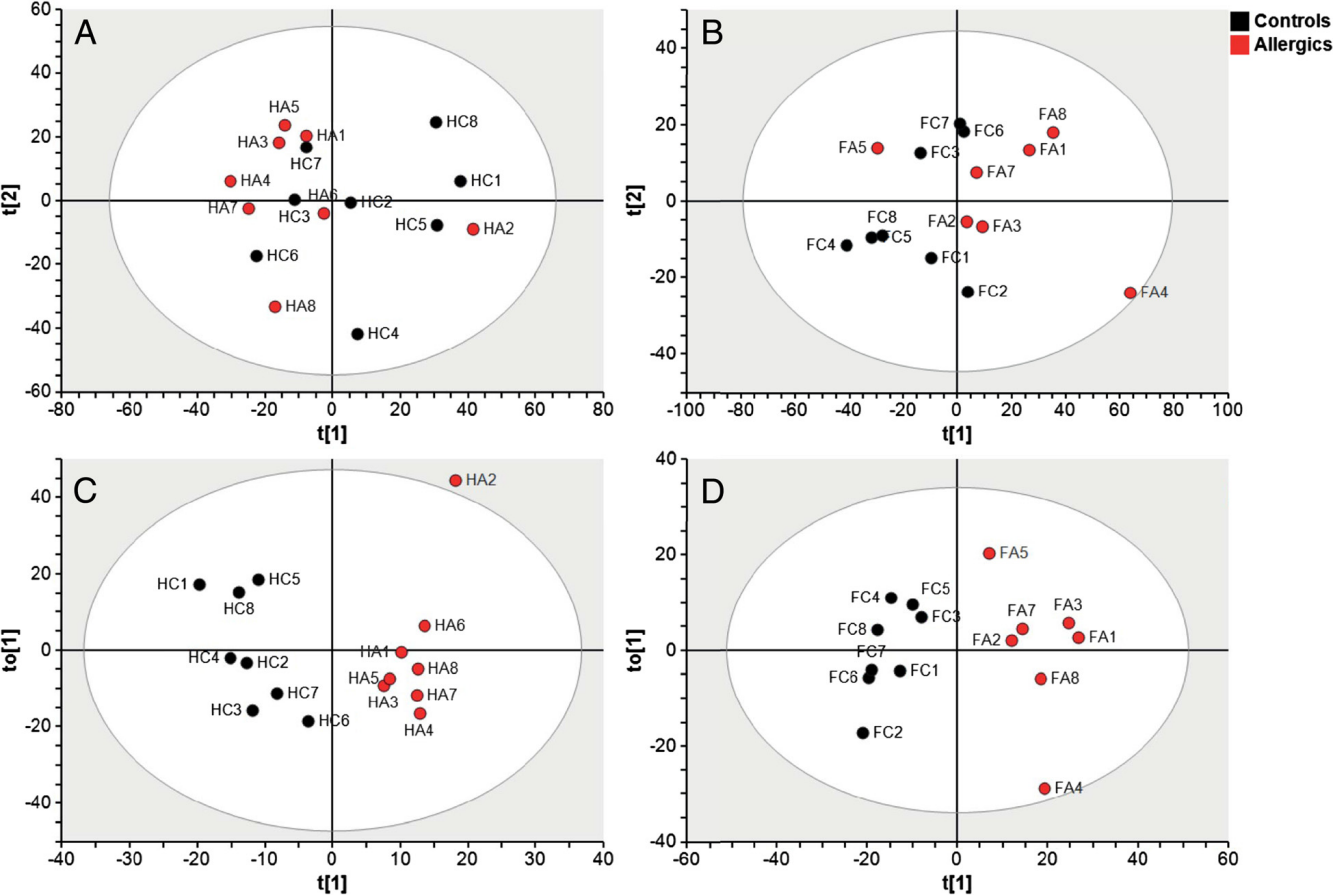
**Allergy induces changes in the brain transcripts as shown by multivariate analysis.** Principal compontent analysis (PCA) and Orthogonal to latent structures discriminant analysis (OPLS-DA) were used to analyze the gene expression in allergic mice (*n* = 8) and control mice (*n* = 8). In the frontal cortex, the number of allergic mice was seven. PCA score plots in the hippocampus **(A)** (*Q*^2^ = 0.415 and *R*^2^ = 0.615) and in the frontal cortex **(B)** (*Q*^2^ = 0.535 and *R*^2^ = 0.692) show an overall view of the data. OPLS-DA score plots indicate group separation between allergic and control mice in the hippocampus **(C)** (*Q*^2^ = 0.488 and *R*^2^ = 0.906) and in the frontal cortex **(D)** (*Q*^2^ = 0.590 and *R*^2^ = 0.961). H = Hippocampus, FC = Frontal cortex, C = Control, A = Allergic.

**Figure 2:**
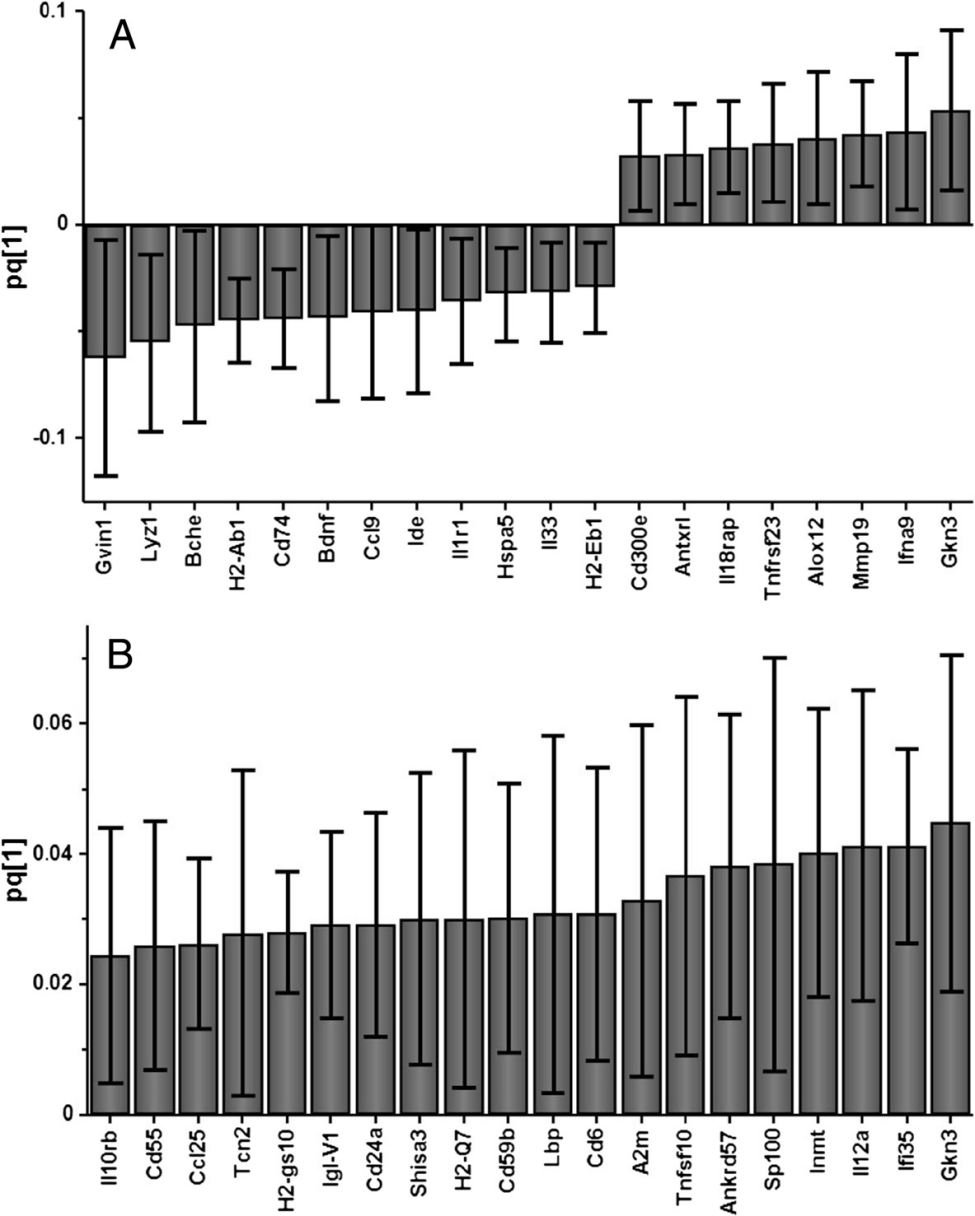
**Allergy induces significant changes of gene expression in the mouse brain.** Loading plots derived from orthogonal projection to latent structures (OPLS-DA) illustrate a set of genes that significantly contributed to separating allergic mice from control mice (*n* = 8) with regard to the hippocampus **(A)** (*n* = 8) and the frontal cortex **(B)** (*n* = 7). The confidence level for each gene is estimated by jackknife algorithm.

Multivariate analysis was performed using the SIMCA P + software package (UMETRICS AB, Umeå, Sweden). Principal component analysis (PCA) was used to provide an overview of the data and to detect possible outliers. Orthogonal projection to latent structures discriminant analysis (OPLS-DA) [16] was performed to detect genes that significantly differed between allergic and control mice. For this purpose, the data were preprocessed by unit variance scaling and detrending. Variables with a high level of variance are more likely to be expressed than those with a low variance. Therefore, unit variance scaling was selected to scale the data appropriately. This method uses the inverse standard deviation (SD) as a scaling weight for each variable. Detrending improves the interpretability of the data by subtracting variable averages from the data; thus the dataset is repositioned around the origin. The score plots (see Figure 1A through 1D) illustrate the deviation from the center of the model projected on the components (to1, to2, to3, …to*n*). The variables that are important for the class separation are visualized in the loading plots (see Figure 2A and 2B).

The number of components in PCA and OPLS-DA depends on *R*^2^ (estimation of fit) and *Q*^2^ (estimation of prediction) values. The *Q*^2^ value calculation is based on sevenfold cross-validation, which means that one-seventh of the data is omitted for each cross-validation round.

Jackknifing is an approach used for finding the precision of an estimate. In PCA and OPLS-DA, jackknifing uncertainty measures of scores and loadings are calculated from the set of multiple models generated from cross-validation [17].

Finally, the significant genes derived from the OPLS-DA model were analyzed with a web-based gene set analysis toolkit (WebGestalt (WEB*-*based GEneSeTAnaLysis Toolkit) version 2.0) [18] according to the instructions given in the software program. Heat maps were used to provide an overview of the DEGs in the respective brain samples. The heat maps were generated using the R statistical software package (GNU General Public License; http://www.r-project.org/).

### Polymerase chain reaction

To confirm the results obtained by microarray analysis, gene expression was analyzed by quantitative polymerase chain reaction (qPCR) using TaqMan probes (Applied Biosystems, Foster City, CA, USA) for TNF receptor superfamily 23 (TNFRS23) (ID Mm00656375_m1), transthyretin (ID Mm004432267_m1) and thyrotropin-releasing hormone (TRH) (ID Mm 01963590_s1) in the hippocampus and lipopolysaccharide-binding protein (LBP) (Mm00493139_m1) in the frontal cortex. The RNA was reverse-transcribed using the TaqMan High Capacity cDNA Reverse Transcription kit (Applied Biosystems). The qPCR was carried out in a total volume of 20 μl in each well containing 10 μl of TaqMan Gene Expression Master Mix (Applied Biosystems), 1 μl of TaqMan probes and 2 μl of cDNA corresponding to 10 ng/μl RNA. The reaction was performed in triplicate, and, for quantification of the expression, the comparative cycle threshold (Ct) method 2^ΔΔCt^ was used, with ΔΔCt = ((Ct_target gene_– Ct_endogenouscontrol_)_allergics_ – (Ct_target gene_ – Ct_endogenous control_)_control_). β-actin (Mm 00607939_s1) was used as an endogenous control.

### Western blot analysis

The hippocampus, frontal cortex and hypothalamus were homogenized by sonication in homogenization buffer consisting of 20 mM Tris•HCl, pH 6.8, containing 137 mM NaCl and 2 mM ethylenediaminetetraacetic acid, 2% Nonidet P-40 and 2% Triton X-100 (Sigma-Aldrich, St Louis, MO, USA), supplemented with a protease inhibitor cocktail (Sigma-Aldrich), phosphatase inhibitors (Halt protease inhibitor cocktail; Pierce Biotechnology, Rockford, IL, USA) and 2 nM okadaic acid (Sigma-Aldrich). The brain tissue homogenates were centrifuged at 4°C for 20 min at 20,000 × *g*, and the supernatants were collected and rapidly frozen. The protein content of the supernatants was determined using a bicinchoninic acid kit (Sigma-Aldrich).

Samples containing equal amounts of proteins (20 to 40 μg) were added to sodium dodecyl sulfate (SDS) in 2 × sample loading buffer (50 mM Tris, pH 6.8, containing 10% glycerol, 0.1% bromophenol blue, 2% SDS and 5% β-mercaptoethanol; Sigma-Aldrich), electrophoresed in 10% SDS polyacrylamide gel and transferred to a 45-μm nitrocellulose membrane (Bio-Rad Laboratories, Hercules, CA, USA). After blocking with 5% nonfat milk in TBS-T (20 mM Tris•HCl, pH 7.4, 137 mM NaCl and 0.1% Tween 20) for 30 min at room temperature, the membrane was incubated overnight at 4°C with primary antibodies (1:1,000 insulin-degrading enzyme (IDE), Abcam, Cambridge, MA, USA; 1:1,000 phosphorylated insulin receptor (p-IR) and 1:1,000 total insulin receptor (t-IR) cell signaling, respectively; 1:2,000 β-actin, cell signaling suggested concentration by manufacturer 1:1000) in blocking solution. The blots were washed with TBS-T for 30 min and incubated with the appropriate secondary antibodies for 2 h at room temperature. After being rinsed in TBS-T for 30 min, the blots were incubated for 5 min with Amersham ECL Prime Western Blotting Detection Reagent (GE Healthcare Life Sciences, Piscataway, NJ, USA), and the chemiluminescence was detected using a charge-coupled device camera (LAS-3000; Fujifilm Life Science, Düsseldorf, Germany). Quantification of signals was performed using Multi Gauge (version 3.0) software (Fuji Photo Film Co, Tokyo, Japan). The protein levels were normalized to β-actin levels, which were used as a housekeeping protein.

### Statistical analysis

The statistical analysis for the validation data was performed using SPSS version 20 statistical software (SPSS, Inc, Chicago, IL, USA) with Student’s *t*-test for normally distributed data.

## Results

### Differentially expressed gene analysis

Increased brain levels of IgG and IgE were found in allergic mice, a finding which is in agreement with our previous data [13]. Microarray analysis revealed changes in brain gene expression in mice with chronic airway allergy, even though most of the genes analyzed were expressed in both allergic and control animals and were not influenced by the allergy. Ten percent of the genes found to be up- or down-regulated by allergy were selected by the means of foldchange. The result of this calculation was 1,488 and 1,459 genes in the hippocampus and frontal cortex, respectively.

PCA was performed for overall analysis to detect possible outliers in the data. Each mouse has its unique expression profile in the variable space, and therefore data that show similar expression profiles are grouped together in the PCA score plot. The results show that the allergic mice differed from control mice, despite some degree of overlap between the groups (Figure 1A and 1B). However, no outliers were detected.

To identify genes that contributed to the differences between allergic and control mice, the data were analyzed by OPLS-DA. It was thus possible to depict a group separation in mice as indicated in the score plots (Figure 1C and 1D). The predictive component in the score plots (t1) indicates that gene expression in allergic mice differs from that in control mice. In addition, the allergic mice cluster shows less variation than control mice in the orthogonal component (to1), possibly reflecting their common allergic phenotype. OPLS-DA identified 257 and 856 significant DEGs in the hippocampus and frontal cortex, respectively. All of the DEGs that contributed to discrimination between the allergic and control mice in each brain region are presented in Additional file 1: Table 1 and Table 2. Some of those genes are presented in loading plots (Figure 2A and 2B).

**Figure 3:**
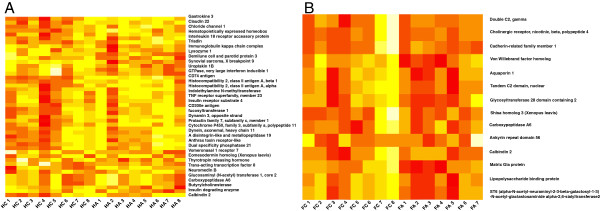
**Allergy induces changes of gene expression in the mouse brain as shown by cluster analysis.** A subset of highly differentially expressed genes (DEGs) was selected from the hippocampus **(A)** and the frontal cortex **(B)**, and the heat maps were generated from log_2_-transformed data using the R statistical software package. H = Hippocampus, FC = Frontal cortex, C = Control, A = Allergic.

The datasets were alternatively analyzed using univariate statistics. To provide an overview of the altered genes in respective regions, a subset of major DEGs based on *P* values and fold changes was selected, log_2_-transformed and visualized as heat maps using the R statistical software package (Figure 3A and 3B). Interestingly, the sample HA2 was identified as an outlier using both statistical approaches (Figures 1C and 3A).

### Functional pathways modified by allergy

Bioinformatics approaches such as pathway analysis provide a tool for interpretation of large gene datasets by putting them in the context of biological processes, pathways and networks. To understand which pathways were altered in the mouse brain due to allergy, we used the WebGestalt version 2.0 algorithm to identify significant functional enrichment in DEGs. The DEGs detected by OPLS-DA were selected for pathway analysis.

**Figure 4:**
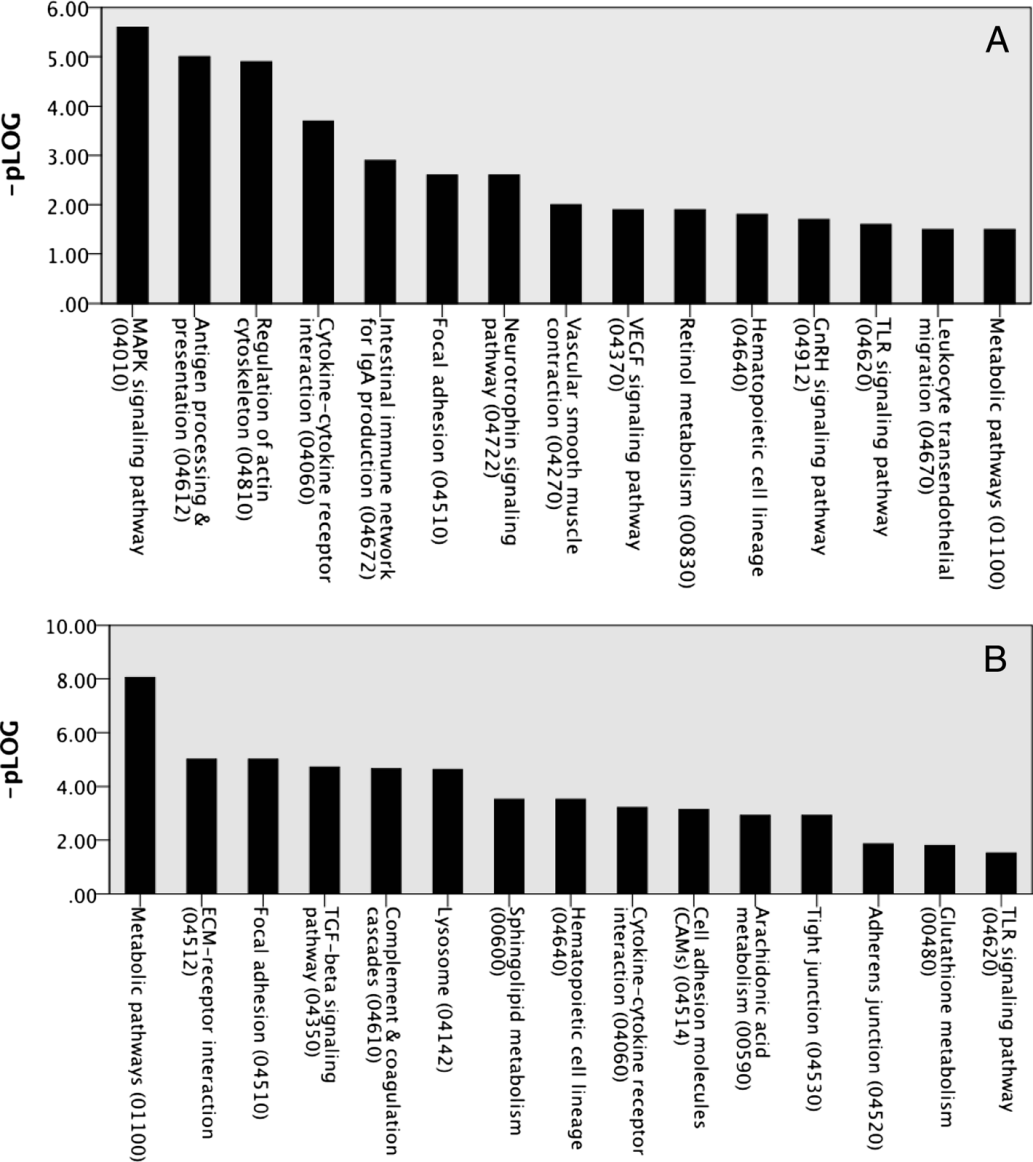
**Pathway analysis of allergy induced differentially expressed genes in the brain.** KEGG (Kyoto Encyclopedia of Genes and Genomes) pathway analysis was performed on significantly altered genes between allergic mice and control mice as analysed with orthogonal projection to latent structures discriminant analysis (OPLS-DA). A subset of the significantly enriched pathways is depicted in hippocampus **(A)** and frontal cortex **(B)**. *–pLog value corresponds to the logarithmized hypergeometric p-value adjusted by the multiple test adjustment.*

The analysis of KEGG (Kyoto Encyclopedia of Genes and Genomes) pathways in the hippocampus and frontal cortex showed significant enrichment for genes involved in several pathways, as depicted in Figure 4A and 4B. The majority of the indicated pathways were involved in inflammatory responses, such as antigen processing and presentation, Toll-like receptor (TLR) signaling, complement and coagulation cascade, JAK-STAT (Janus kinase and signal transducer and activator of transcription) signaling and cytokine–cytokine interactions. The genes that were altered by allergy in some of the pathways are shown in Additional file 2: Table 3 and Table 4 for hippocampus and frontal cortex, respectively.

### Validation of microarrays

**Figure 5:**
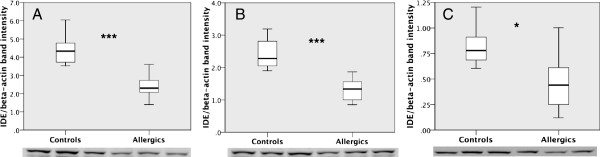
**Allergy decreases the levels of insulin-degrading enzyme (IDE) in the mouse brain.** Western blot analysis showed a decrease in IDE levels in the hippocampus **(A)**, frontal cortex **(B)** and hypothalamus of allergic mice compared to controls **(C)**. The data were analyzed using Student’s *t*-test. The boxplot indicates the first two quartiles with the median, and the whiskers indicate the third and fourth quartiles (*n* = 8 mice per group). Only six control mice were used for the hypothalamus because two samples were lost during sonication. The images below the graph indicate the immunoblotting of IDE. *Asterisk, p < 0.05, triple asterisk p < 0.001*.

**Figure 6:**
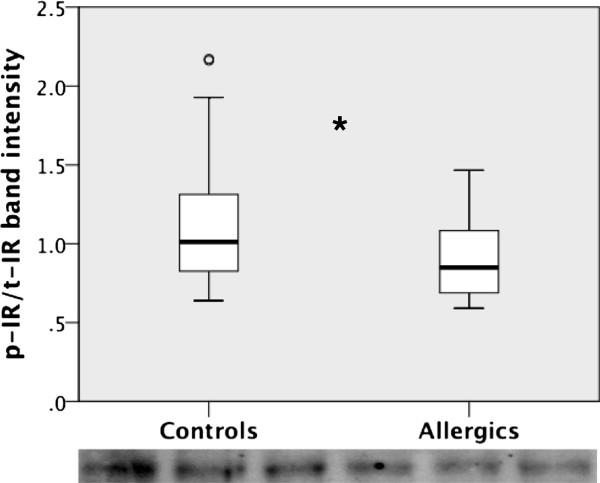
**Allergy decreases the insulin receptor phosphorylation in the mouse brain.** The levels of phosphorylated insulin receptor (p-IR) were significantly reduced in the hippocampus of allergic mice compared to controls. The data were analyzed using Student’s *t*-test. The boxplot shows the first two quartiles with the median, and the whiskers indicate the third and fourth quartiles (*n* = 16 for the allergic group and *n* = 17 for the control group). t-IR, total insulin receptor. The images below the graph show the immunoblotting of p-IR. *Asterisk, p < 0.05,****°****indicates outliers.*

The microarray data showed that allergy was associated with a reduction in IDE, which mediates cleavage of insulin and amyloid-β (Aβ), important proteins in diabetes mellitus and AD, respectively. Western blot analysis of the protein levels showed a significant decrease in IDE in both the hippocampus (Figure 5A) [95% confidence interval (CI) 0.64-1.57; *P* < 0.001] and the frontal cortex (Figure 5B) [95% CI 1.19-2.82; *P* < 0.001] of allergic mice compared to controls. Since insulin signaling in the hypothalamus plays an important role in the regulation of glucose metabolism, the hypothalamic IDE levels were measured. The IDE levels were significantly reduced in the hypothalamus from allergic mice [95% CI 0.59-0.66;*P* < 0.05] (Figure 5C). As insulin is a major substrate for IDE, we further analyzed whether changes in IDE could have a functional impact on insulin signaling. The levels and phosphorylation status of IRs were analyzed by immunoblotting. t-IR levels were similar in allergic and control mice; however, allergy was associated with a significant decrease in the levels of p-IR [95% CI 0.01- 0.31;*P <* 0.05] in the hippocampus (Figure 6) but not in the frontal cortex and hypothalamus, although the data showed a trend similar to that in the hippocampus (data not shown). We also analyzed the expression of transthyretin and TRH by qPCR; however, we found no changes between the groups (data not shown), as indicated by the microarray data.

**Figure 7:**
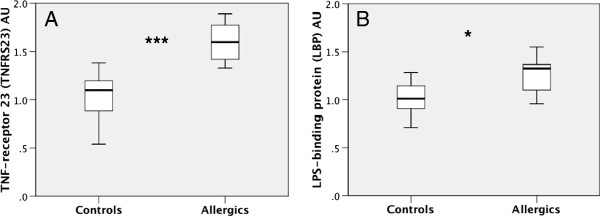
**Confirmation of allergy induced changes in gene expression by quantitative PCR.** RNA was reverse-transcribed and probed for the tumor necrosis factor receptor superfamily member 23 (TNFRS23) in the hippocampus **(A)** and lipopolysaccharide-binding protein (LPB) in the frontal cortex **(B)**. The qPCR results are in accord with the data obtained from microarray analysis. The data were analyzed using Student’s *t*-test. The boxplot indicates the first two quartiles with the median, and the whiskers indicate the third and fourth quartiles (n = 8 mice per group). Asterisk, p< 0.05, triple asterisk, p< 0.001. *AU, arbitrary units.*

We have further validated other significant changes provided by the microarray analysis with qPCR for TNFRS23 in the hippocampus (Figure 7A) and LBP in the frontal cortex (Figure 7B). The results were in line with the microarray data. Thus, the levels of TNFRS23 were increased in the hippocampus of allergic mice [95% CI −0.82- −0.31; *P* < 0.001] and LBP levels were increased in the frontal cortex [95% CI −0.49 - −0.04; *P* < 0.05].

## Discussion

The previously reported association between allergic diseases and dementia [14], as well as the inflammatory changes induced by allergy in the brain [12,13], represents a basis for further studies on the effects of allergy on the brain. We have analyzed gene expression in mouse brains and found that allergy led to changes in the genes and/or proteins involved in the insulin-signaling pathway, in inflammatory pathways and in other pathways.

Inflammation has been shown to play an important role in the development of neurodegenerative diseases [5]. The present microarray data show that several inflammatory pathways were altered in the brain. Furthermore, signaling pathways found to be modified in the allergic mouse brain, including neurotrophin signaling, TLR-signaling, complement and coagulation cascades, and cytokine–cytokine interactions, have been pointed out in AD [19]. In view of these findings, it may be assumed that allergy may render the brain more susceptible to challenges later in life.

The mRNA expression for TNFRS23 and LBP was increased in the allergic brain, consistent with the results obtained from the microarrays. TNFR23 is a decoy receptor with unknown function. Clark *et al*. reported its involvement in parental imprinting [20], and a subsequent study showed an immunomodulatory role by inhibition of T-cell proliferation [21]. In view of these findings, the increase in TNFR23 mRNA upon allergy may be interpreted as a means to modulate an inflammatory response induced by the allergy. LBP is an acute-phase protein mainly produced in the liver. In brain, LBP expression has been detected in the hippocampus and choroid plexus [22]. Together with CD14, LBP is involved in enhancing the strength of LPS-induced responses to type I interferons (IFNs) [23]. In the present study, the transcripts of several type I IFNs were found to be increased in the frontal cortex, indicating that TLR4-signaling may be altered in the allergic brains.

The difference in gene expression was moderate with regard to foldchange in allergic mice compared to control mice in both the hippocampus and the frontal cortex. The use of one time point for the analysis may be a limitation in that early transient changes in gene expression may not be found. This is supported by a previous report based on a chronic allergy model in which the gene expression in the lungs was not only associated with low-grade changes but also characterized by a return to baseline levels of a large number of genes [24]. However, it is likely that chronic conditions may be associated with low-grade changes in contrast to acute conditions and that accumulation of the effects of these gradual and/or transitory changes may have functional consequences over time.

Our data show that allergy was associated with a significant reduction in the IDE levels in the brain. IDE plays a key role in the degradation of insulin and Aβ, classic hallmarks of diabetes mellitus and AD, respectively. Macrophage inflammatory protein 1, a C-C motif chemokine with proinflammatory properties, has been identified as a substrate for IDE [25], raising the possibility that IDE may play a role in suppressing inflammatory responses. A reduction of IDE in response to allergy may thus result in increased levels of C-C motif–containing chemokines. Notably, reduced IDE levels have been found in the brains of AD patients and in mouse models of AD [26], thus rendering a potential link between allergy, insulin-related deficits and AD.

IDE is widely expressed in the brain, especially in neurons; but other cells, including oligodendrocytes, microglia and astrocytes, are putative sources [27-29]. Analysis of the regional distribution pattern of IDE in human brain showed intense staining in brain regions known for their high levels of insulin and insulin receptors, that is, the cerebral cortex and the hippocampus [30]. The observation of reduced IDE levels in the brains of allergic mice motivated further studies on the effects in insulin receptor signaling. In agreement with data from the microarray analysis, there was no significant difference in insulin receptor protein levels between allergic and control mice. Phosphorylation of the insulin receptor is necessary for signaling, and we observed a decreased phosphorylation of the insulin receptor in the hippocampus. The phosphorylation of the insulin receptor in the hypothalamus did not reach significance, presumably due to the small numbers of animals investigated. The observed decrease in levels of the phosphorylated form of this receptor upon allergy suggests an allergy-induced inhibition of insulin signaling in the brain. Insulin signaling in the brain plays an important role in the regulation of peripheral fat and glucose metabolism [31], and deficits in brain insulin signaling have been linked to development of diabetes type 2 (DT2) and obesity [32]. Mice lacking neuronal insulin receptors were found to be obese and showed increased peripheral insulin resistance and hypertriglyceridemia [33]. Previously, it was shown that chronic exposure to TNF-α decreased insulin receptor phosphorylation in adipocytes [34] and that increased levels of TNF-α, IL-6 and IL-1β are linked to systemic inflammation and accompany insulin resistance [35]. In view of these studies and the present findings, it would be interesting to study whether mice with allergy-associated inflammation develop insulin resistance.

In addition to its peripheral actions, insulin has been shown to enhance memory formation [36], presumably by binding to receptors in the hippocampus and adjacent limbic structures that are important for memory. Impaired insulin signaling has been implicated in AD [32], thus underscoring a shared dysregulated pathway between a cognitive disease and a metabolic disorder.

Asthma is associated with DT2 and obesity [37,38], both of which are metabolic disorders with an underlying systemic inflammatory profile. Together with our data, this suggests that systemic inflammation associated with allergy may modify insulin signaling in the brain, which could have consequences for brain function and the pathophysiology of some neurodegenerative disorders.

Analysis at the gene level is advantageous in providing an overview of the transcription in a given biological system, but is insufficient by itself to describe posttranscriptional biological events, including mechanisms controlling the protein translational rate, the half-life of mRNA or protein and the intracellular localization and posttranslational modification of the proteins [39].

In summary, our results show that airway inflammation associated with allergy influences the brain with regard to proteins involved in insulin signaling and genes involved in inflammation, as well as other functional pathways. These results may have implications for further understanding the mechanisms behind an association of chronic inflammation such as allergy with endocrine disorders such as DT2 and obesity and neurodegenerative disorders such as AD, all of which share an ongoing inflammatory component as a common denominator.

## Abbreviations

AD: Alzheimer disease; AIC: Anterior insular cortex; DEG: Differentially expressed gene; DT2: Diabetes type 2; IDE: Insulin-degrading enzyme; Ig: Immunoglobulin; LBP: Lipopolysaccharide-binding protein; OPLS-DA: Orthogonal to latent structures discriminant analysis; OVA: Ovalbumin; PCA: Principal component analysis; Th2: T-helper type 2; TNFRS23: TNF receptor superfamily member 23.

## Competing interests

All authors declare that they have no competing interests.

## Authors’ contribution

HS designed and carried out the experiments,performed data analysis and wrote the manuscript. XW contributed to the allergy induction experiments and data analysis. ACM, MS and MO designed the research, provided supervision and assisted in data analysis and manuscript editing. MS and MO received the grant support. All of the authors read and approved the final manuscript.

## Supplementary Material

Additional file 1: Table 1 Allergy-induced differentially expressed genes (DEGs) in the hippocampus. A total number of 1,488 hippocampal genes were included in orthogonal projection to latent structure discriminant analysis (OPLS-DA) to detect DEGs in allergic mice compared to controls. All 257 genes that were found to significantly contribute to group separation are listed. **Table 2.** Allergy-induced differentially expressed genes (DEGs) in the frontal cortex. A total number of 1,459 frontal cortical genes were subjected to orthogonal projection to latent structure discriminant analysis (OPLS-DA) to detect DEGs in allergic mice compared to controls. All 856 genes that were found to significantly contribute to group separation are listed. Click here for file

Additional file 2: Table 3 Genes involved in enrichment of KEGG^a^ pathways in the hippocampus. Significant differentially expressed genes (DEGs) between allergic mice compared to controls were detected by orthogonal projection to latent structures discriminant analysis (OPLS-DA) and subsequently subjected to pathway analysis. Genes that were involved in significantly enriching the indicated KEGG pathways in the hippocampus are shown in the list. ^a^MAPK, mitogen activated protein kinase. **Table 4.** Genes involved in enrichment of KEGG^a^ pathways in the frontal cortex. Significant differentially expressed genes (DEGs) between allergic mice compared to controls were detected by orthogonal projection to latent structures discriminant analysis (OPLS-DA) and subsequently subjected to pathway analysis. Genes that were involved in significantly enriching the indicated KEGG pathways in the frontal cortex are shown in the list. ^a^Kyoto Encyclopedia of Genes and Genomes. Click here for file
