# Supplementary material for: Chronic airway-induced allergy in mice modifies gene expression in the brain toward insulin resistance and inflammatory responses
Source: J Neuroinflammation. 2013 Aug 1;10:99. doi: 10.1186/1742-2094-10-99 (PMC3750454; doi:10.1186/1742-2094-10-99)
Supplement: Additional file 1: Table 1 — Allergy-induced differentially expressed genes (DEGs) in the hippocampus. A total number of 1,488 hippocampal genes were included in orthogonal projection to latent structure discriminant analysis (OPLS-DA) to detect DEGs in allergic mice compared to controls. All 257 genes that were found to significantly contribute to group separation are listed. Table 2. Allergy-induced differentially expressed genes (DEGs) in the frontal cortex. A total number of 1,459 frontal cortical genes were subjected to orthogonal projection to latent structure discriminant analysis (OPLS-DA) to detect DEGs in allergic mice compared to controls. All 856 genes that were found to significantly contribute to group separation are listed. [file 1742-2094-10-99-S1.docx]

**Table 1**

A total number of 1,488 hippocampal genes were included in OPLS-DA analysis. The 257 genes that significantly contributed to group separation are listed.

^a^The Entrez ID of genes; ^b^Fold change in gene expression in allergic mice with regard to controls;

^c^Subtraction of absolute (Abs) values of jack-knife confidence interval (JKCI) from Abs values of Loadings derived from the cross-validation of the OPLS-DA model. Positive values indicate significance. ^d^p-values calculated by using student’s t-test.

| Gene symbol | Gene name | Probeset | Entrez^a^ | F-C^b^ | Sign^c^ | p-values^d^ |
| --- | --- | --- | --- | --- | --- | --- |
| Agpat5 | 1-acylglycerol-3-phosphate O-acyltransferase 5 (lysophosphatidic acid acyltransferase, epsilon) | 10570573 | 52123 | -1.10 | 0.0022 | 0.0112 |
| Oasl2 | 2'-5' oligoadenylate synthetase-like 2 | 10524621 | 23962 | 1.17 | 0.0063 | 0.0111 |
| Pfkfb3 | 6-phosphofructo-2-kinase/fructose-2,6-biphosphatase 3 | 10480035 | 170768 | -1.14 | 0.0145 | 0.0079 |
| Adamts19 | a disintegrin-like and metallopeptidase (reprolysin type) with thrombospondin type 1 motif, 19 | 10455919 | 240322 | 1.35 | 0.0054 | 0.0232 |
| Actn3 | actinin alpha 3 | 10464836 | 11474 | -1.15 | 0.0079 | 0.0081 |
| Arfip2 | ADP-ribosylation factor interacting protein 2 | 10566502 | 76932 | -1.10 | 0.0003 | 0.0103 |
| Adm2 | adrenomedullin 2 | 10426208 | 223780 | 1.24 | 0.0056 | 0.0040 |
| Acan | aggrecan | 10554249 | 11595 | 1.12 | 0.0000 | 0.0118 |
| Aard | alanine and arginine rich domain containing protein | 10424082 | 239435 | -1.14 | 0.0082 | 0.1266 |
| Amy2a5 | amylase 2a5 | 10501544 | 109959 | 1.11 | 0.0074 | 0.0080 |
| Antxrl | anthrax toxin receptor-like | 10414047 | 239029 | 1.38 | 0.0095 | 0.0027 |
| Alox12 | arachidonate 12-lipoxygenase | 10387821 | 11684 | 1.24 | 0.0094 | 0.0052 |
| Asap1 | ArfGAP with SH# domain, ankyrin repeat and PH domain1 | 10428998 | 13196 | -1.10 | 0.0040 | 0.0157 |
| B4galnt3 | beta-1,4-N-acetyl-galactosaminyl transferase 3 | 10541214 | 330406 | 1.21 | 0.0027 | 0.0138 |
| Bdnf | brain derived neurotrophic factor | 10474399 | 12064 | -1.19 | 0.0049 | 0.0086 |
| Brd3 | bromodomain containing 3 | 10481240 | 67382 | -1.12 | 0.0016 | 0.0085 |
| Bche | butyrylcholinesterase | 10498710 | 12038 | -1.29 | 0.0025 | 0.0050 |
| Mertk | c-mer proto-oncogene tyrosine kinase | 10475890 | 17289 | -1.11 | 0.0073 | 0.0087 |
| Cacna1d | calcium channel, voltage-dependent, L type, alpha 1D subunit | 10418355 | 12289 | 1.12 | 0.0136 | 0.0085 |
| Chst2 | carbohydrate sulfotransferase 2 | 10595718 | 54371 | -1.12 | 0.0033 | 0.0092 |
| Car12 | carbonic anyhydrase 12 | 10586591 | 76459 | -1.14 | 0.0029 | 0.0079 |
| Cpa6 | carboxypeptidase A6 | 10353102 | 329093 | 1.38 | 0.0039 | 0.0386 |
| Cd24a | CD24a antigen | 10362896 | 12484 | 1.15 | 0.0121 | 0.0065 |
| Cd300e | CD300e antigen | 10392839 | 217306 | 1.38 | 0.0065 | 0.0073 |
| Cd74 | CD74 antigen (invariant polypeptide of major histocompatibility complex, class II antigen-associated) | 10456005 | 16149 | -1.32 | 0.0203 | 0.0040 |
| Cenpj | centromere protein J | 10420320 | 219103 | 1.16 | 0.0077 | 0.0066 |
| Cdr2l | cerebellar degeneration-related protein 2-like | 10382502 | 237988 | -1.13 | 0.0134 | 0.0077 |
| Ccl9 | chemokine (C-C motif) ligand 9 | 10389214 | 20308 | -1.16 | 0.0003 | 0.0078 |
| Clcn1 | chloride channel 1 | 10537742 | 12723 | 1.28 | 0.0171 | 0.0092 |
| Ccdc163 | coiled-coil domain containing 163 | 10507334 | 68394 | 1.21 | 0.0141 | 0.0104 |
| Ccdc86 | coiled-coil domain containing 86 | 10466104 | 108673 | -1.12 | 0.0017 | 0.0068 |
| Col6a1 | collagen, type VI, alpha 1 | 10370210 | 12833 | 1.11 | 0.0034 | 0.0331 |
| Col25a1 | collagen, type XXV, alpha 1 | 10496036 | 77018 | -1.12 | 0.0022 | 0.0097 |
| Cplx2 | complexin 2 | 10405253 | 12890 | -1.12 | 0.0000 | 0.0075 |
| Cbfa2t3 | core-binding factor, runt domain, alpha subunit 2, translocated to, 3 (human) | 10582429 | 12398 | -1.13 | 0.0045 | 0.0066 |
| Creld2 | cysteine-rich with EGF-like domains 2 | 10426098 | 76737 | -1.15 | 0.0072 | 0.0045 |
| Cyb5r4 | cytochrome b5 reductase 4 | 10587627 | 266690 | -1.11 | 0.0034 | 0.0115 |
| Cox17 | cytochrome c oxidase, subunit XVII assembly protein homolog (yeast) | 10435693 | 12856 | -1.14 | 0.0019 | 0.0072 |
| Cyp2s1 | cytochrome P450, family 2, subfamily s, polypeptide 1 | 10561128 | 74134 | -1.14 | 0.0007 | 0.0097 |
| Cyp26b1 | cytochrome P450, family 26, subfamily b, polypeptide 1 | 10545771 | 232174 | -1.20 | 0.0084 | 0.0091 |
| Dhrs9 | dehydrogenase/reductase (SDR family) member 9 | 10472538 | 241452 | 1.17 | 0.0039 | 0.0039 |
| Dio2 | deiodinase, iodothyronine, type II | 10401841 | 13371 | -1.20 | 0.0097 | 0.0102 |
| Dohh | deoxyhypusine hydroxylase/monooxygenase | 10365116 | 102115 | -1.17 | 0.0008 | 0.0081 |
| Dgat2l6 | diacylglycerol O-acyltransferase 2-like 6 | 10600980 | 668257 | 1.21 | 0.0005 | 0.0093 |
| Dab2ip | disabled homolog 2 (Drosophila) interacting protein | 10471677 | 69601 | -1.13 | 0.0035 | 0.0106 |
| Dlx5 | distal-less homeobox 5 | 10543058 | 13395 | 1.19 | 0.0055 | 0.0386 |
| Dusp4 | dual specificity phosphatase 4 | 10571312 | 319520 | -1.20 | 0.0021 | 0.0084 |
| Dnahc11 | dynein, axonemal, heavy chain 11 | 10403112 | 13411 | 1.50 | 0.0181 | 0.0032 |
| Ear2 | eosinophil-associated, ribonuclease A family, member 2 | 10414262 | 13587 | 1.19 | 0.0028 | 0.0082 |
| Ephb3 | Eph receptor B3 | 10434559 | 13845 | -1.11 | 0.0043 | 0.0107 |
| Epb4.1l4a | erythrocyte protein band 4.1-like 4a | 10458052 | 13824 | 1.18 | 0.0012 | 0.0061 |
| Eef1d | eukaryotic translation elongation factor 1 delta (guanine nucleotide exchange protein) | 10429657 | 66656 | -1.10 | 0.0180 | 0.0079 |
| Eif2ak3 | eukaryotic translation initiation factor 2 alpha kinase 3 | 10538957 | 13666 | 1.16 | 0.0029 | 0.0104 |
| Ezr | ezrin | 10447602 | 22350 | -1.11 | 0.0005 | 0.0060 |
| Fbxo34 | F-box protein 34 | 10414366 | 78938 | -1.12 | 0.0034 | 0.0102 |
| Fam107a | family with sequence similarity 107, member A | 10417561 | 268709 | -1.14 | 0.0026 | 0.0082 |
| Fam184a | family with sequence similarity 184, member A | 10369132 | 75906 | 1.12 | 0.0087 | 0.0104 |
| Fam194a | family with sequence similarity 194, member A | 10498323 | 545527 | -1.14 | 0.0033 | 0.0122 |
| Fam70a | family with sequence similarity 70, member A | 10604175 | 245386 | 1.14 | 0.0000 | 0.0065 |
| Fitm2 | fat storage-inducing transmembrane protein 2 | 10489368 | 228859 | -1.13 | 0.0019 | 0.0074 |
| Frmpd1 | FERM and PDZ domain containing 1 | 10504534 | 666060 | -1.10 | 0.0056 | 0.0066 |
| Frmd7 | FERM domain containing 7 | 10604508 | 385354 | 1.69 | 0.0030 | 0.0940 |
| Fbn2 | fibrillin 2 | 10458999 | 14119 | 1.20 | 0.0094 | 0.0073 |
| Fgl2 | fibrinogen-like protein 2 | 10519983 | 14190 | -1.13 | 0.0031 | 0.0069 |
| Fgf5 | fibroblast growth factor 5 | 10523490 | 14176 | -1.11 | 0.0148 | 0.0131 |
| Fndc1 | fibronectin type III domain containing 1 | 10447649 | 68655 | 1.16 | 0.0005 | 0.0045 |
| Foxo6 | forkhead box O6 | 10515939 | 329934 | -1.12 | 0.0072 | 0.0036 |
| Fhod3 | formin homology 2 domain containing 3 | 10454369 | 225288 | -1.12 | 0.0021 | 0.0321 |
| Fosl2 | fos-like antigen 2 | 10520862 | 14284 | -1.17 | 0.0150 | 0.0070 |
| Frat1 | frequently rearranged in advanced T-cell lymphomas | 10463164 | 14296 | -1.11 | 0.0101 | 0.0078 |
| Gabpb2 | GA repeat binding protein, beta 2 | 10500103 | 213054 | 1.14 | 0.0063 | 0.0086 |
| Gjb6 | gap junction protein, beta 6 | 10420366 | 14623 | -1.15 | 0.0005 | 0.0082 |
| Gkn3 | gastrokine 3 | 10546001 | 68888 | 1.34 | 0.0160 | 0.0099 |
| Giyd2 | GIY-YIG domain containing 2 | 10568011 | 75764 | 1.20 | 0.0128 | 0.0094 |
| Glipr1 | GLI pathogenesis-related 1 (glioma) | 10372410 | 73690 | -1.16 | 0.0007 | 0.0051 |
| Glt1d1 | glycosyltransferase 1 domain containing 1 | 10525942 | 319804 | -1.13 | 0.0004 | 0.0108 |
| Gadd45b | growth arrest and DNA-damage-inducible 45 beta | 10364950 | 17873 | -1.19 | 0.0174 | 0.0054 |
| Gvin1 | GTPase, very large interferon inducible 1 | 10566574 | 74558 | -1.50 | 0.0066 | 0.0127 |
| Gbp2 | guanylate binding protein 2 | 10496592 | 14469 | -1.14 | 0.0104 | 0.0066 |
| Hsph1 | heat shock 105kDa/110kDa protein 1 | 10535904 | 15505 | -1.13 | 0.0008 | 0.0109 |
| Hspb1 | heat shock protein 1 | 10408928 | 15507 | -1.17 | 0.0085 | 0.0120 |
| Hspa5 | heat shock protein 5 | 10471586 | 14828 | -1.17 | 0.0103 | 0.0059 |
| Helt | Hey-like transcription factor (zebrafish) | 10578547 | 234219 | 1.15 | 0.0010 | 0.0069 |
| Hmgb2 | high mobility group box 2 | 10518350 | 97165 | -1.11 | 0.0015 | 0.0086 |
| H2-Aa | histocompatibility 2, class II antigen A, alpha | 10450154 | 14960 | -1.37 | 0.0200 | 0.0029 |
| H2-Ab1 | histocompatibility 2, class II antigen A, beta 1 | 10444291 | 14961 | -1.53 | 0.0244 | 0.0013 |
| H2-Eb1 | histocompatibility 2, class II antigen E beta | 10444298 | 14969 | -1.18 | 0.0083 | 0.0061 |
| Hist1h1b | histone cluster 1, H1b | 10408081 | 56702 | -1.12 | 0.0128 | 0.0116 |
| Hist1h1c | histone cluster 1, H1c | 10404059 | 50708 | -1.19 | 0.0276 | 0.0084 |
| Hist1h2be | histone cluster 1, H2be | 10408212 | 319179 | -1.12 | 0.0075 | 0.0088 |
| Homer1 | homer homolog 1 (Drosophila) | 10406626 | 26556 | -1.13 | 0.0026 | 0.0154 |
| Htra1 | HtrA serine peptidase 1 | 10558150 | 56213 | -1.12 | 0.0012 | 0.0094 |
| Hpvc-ps | human papillomavirus 18 E5 central sequence motif, pseudogene | 10534056 | 15456 | 1.21 | 0.0003 | 0.0048 |
| Ier5l | immediate early response 5-like | 10481491 | 72500 | -1.12 | 0.0091 | 0.0122 |
| Irgm2 | immunity-related GTPase family M member 2 | 10376326 | 54396 | -1.14 | 0.0186 | 0.0089 |
| Inmt | indolethylamine N-methyltransferase | 10544932 | 21743 | 1.36 | 0.0015 | 0.0076 |
| Incenp | inner centromere protein | 10465861 | 16319 | -1.13 | 0.0127 | 0.0110 |
| Inpp5j | inositol polyphosphate 5-phosphatase J | 10383717 | 170835 | 1.19 | 0.0081 | 0.0099 |
| Ide | insulin degrading enzyme | 10467230 | 15925 | -1.30 | 0.0020 | 0.0059 |
| Itgb1bp2 | integrin beta 1 binding protein 2 | 10601178 | 26549 | 1.19 | 0.0098 | 0.0083 |
| Ifna9 | interferon alpha 9 | 10514296 | 15972 | 1.18 | 0.0071 | 0.0106 |
| Iigp1 | interferon inducible GTPase 1 | 10455961 | 60440 | 1.15 | 0.0066 | 0.0061 |
| Il1r1 | interleukin 1 receptor, type I | 10345762 | 16177 | -1.20 | 0.0064 | 0.0206 |
| Il17ra | interleukin 17 receptor A | 10541246 | 16172 | -1.13 | 0.0166 | 0.0146 |
| Il18rap | interleukin 18 receptor accessory protein | 10345824 | 16174 | 1.26 | 0.0146 | 0.0061 |
| Il33 | interleukin 33 | 10462442 | 77125 | -1.14 | 0.0083 | 0.0065 |
| Iqcf5 | IQ motif containing F5 | 10588542 | 75470 | 1.13 | 0.0134 | 0.0090 |
| Kcns1 | K+ voltage-gated channel, subfamily S, 1 | 10489422 | 16538 | -1.17 | 0.0073 | 0.0085 |
| L2hgdh | L-2-hydroxyglutarate dehydrogenase | 10400742 | 217666 | 1.24 | 0.0062 | 0.0089 |
| Lgals3 | lectin, galactose binding, soluble 3 | 10414360 | 16854 | -1.12 | 0.0111 | 0.0082 |
| Lingo3 | leucine rich repeat and Ig domain containing 3 | 10370999 | 237403 | -1.13 | 0.0052 | 0.0120 |
| Lrrc23 | leucine rich repeat containing 23 | 10547820 | 16977 | 1.31 | 0.0222 | 0.0084 |
| Lgr5 | leucine rich repeat containing G protein coupled receptor 5 | 10372503 | 14160 | 1.45 | 0.0004 | 0.1247 |
| Lgr6 | leucine-rich repeat-containing G protein-coupled receptor 6 | 10357965 | 329252 | 1.55 | 0.0050 | 0.0609 |
| Ltk | leukocyte tyrosine kinase | 10486262 | 17005 | -1.11 | 0.0018 | 0.0110 |
| Lmo2 | LIM domain only 2 | 10474201 | 16909 | -1.12 | 0.0066 | 0.0146 |
| Laptm5 | lysosomal-associated protein transmembrane 5 | 10508663 | 16792 | -1.11 | 0.0017 | 0.0102 |
| Lyz1 | lysozyme 1 | 10372652 | 17110 | -1.34 | 0.0132 | 0.0066 |
| Lyz2 | lysozyme 2 | 10372648 | 17105 | -1.19 | 0.0059 | 0.0021 |
| Mmp19 | matrix metallopeptidase 19 | 10367400 | 58223 | 1.24 | 0.0176 | 0.0056 |
| Med4 | mediator of RNA polymerase II transcription, subunit 4 homolog (yeast) | 10603706 | 67381 | -1.26 | 0.0064 | 0.0031 |
| Meis1 | Meis homeobox 1 | 10384504 | 17268 | 1.20 | 0.0138 | 0.0276 |
| Manf | mesencephalic astrocyte-derived neurotrophic factor | 10596575 | 74840 | -1.17 | 0.0126 | 0.0058 |
| H2-gs10 | MHC class I like protein GS10 | 10444814 | 436493 | -1.16 | 0.0086 | 0.0062 |
| Mir26a-1 | microRNA 26a-1 | 10590071 | 387218 | 1.39 | 0.0158 | 0.0045 |
| Mir490 | microRNA 490 | 10537296 | 735279 | 1.29 | 0.0019 | 0.0238 |
| Map2k2 | mitogen-activated protein kinase kinase 2 | 10364971 | 26396 | -1.11 | 0.0004 | 0.0111 |
| Map3k1 | mitogen-activated protein kinase kinase kinase 1 | 10412100 | 26401 | 1.22 | 0.0047 | 0.0100 |
| Map3k5 | mitogen-activated protein kinase kinase kinase 5 | 10361926 | 26408 | -1.12 | 0.0050 | 0.0121 |
| Mucl1 | mucin-like 1 | 10433179 | 20771 | 1.19 | 0.0082 | 0.0069 |
| Mustn1 | musculoskeletal, embryonic nuclear protein 1 | 10413609 | 66175 | -1.15 | 0.0014 | 0.0131 |
| Mdfic | MyoD family inhibitor domain containing | 10536472 | 16543 | -1.11 | 0.0015 | 0.0031 |
| Myo16 | myosin XVI | 10570029 | 244281 | 1.18 | 0.0077 | 0.0231 |
| Myh9 | myosin, heavy polypeptide 9, non-muscle | 10430201 | 17886 | -1.11 | 0.0044 | 0.0076 |
| Myl4 | myosin, light polypeptide 4 | 10381798 | 17896 | -1.29 | 0.0231 | 0.0827 |
| Mylk | myosin, light polypeptide kinase | 10435345 | 107589 | -1.15 | 0.0061 | 0.0059 |
| Npr1 | natriuretic peptide receptor 1 | 10499811 | 18160 | 1.20 | 0.0038 | 0.0046 |
| Nxph1 | neurexophilin 1 | 10536405 | 18231 | 1.18 | 0.0036 | 0.0117 |
| Nmb | neuromedin B | 10565067 | 68039 | 1.26 | 0.0051 | 0.0057 |
| Npffr2 | neuropeptide FF receptor 2 | 10523048 | 104443 | 1.30 | 0.0048 | 0.1051 |
| Npy | neuropeptide Y | 10538247 | 109648 | -1.10 | 0.0011 | 0.0241 |
| Naip5 | NLR family, apoptosis inhibitory protein 5 | 10411611 | 17951 | -1.11 | 0.0037 | 0.0072 |
| Nfic | nuclear factor I/C | 10371176 | 18029 | -1.13 | 0.0038 | 0.0113 |
| Nfix | nuclear factor I/X | 10573427 | 18032 | -1.22 | 0.0008 | 0.0062 |
| Nupr1 | nuclear protein 1 | 10567995 | 56312 | -1.15 | 0.0183 | 0.0213 |
| Nol4 | nucleolar protein 4 | 10457836 | 319211 | -1.13 | 0.0154 | 0.0190 |
| Nudt3 | nudix (nucleotide diphosphate linked moiety X)-type motif 3 | 10449363 | 56409 | -1.13 | 0.0000 | 0.0098 |
| Olfr111 | olfactory receptor 111 | 10445141 | 545205 | 1.22 | 0.0055 | 0.0296 |
| Olfr1229 | olfactory receptor 1229 | 10484805 | 257921 | 1.22 | 0.0019 | 0.0110 |
| Olfr140 | olfactory receptor 140 | 10484861 | 57272 | 1.26 | 0.0154 | 0.0085 |
| Olfr1507 | olfactory receptor 1507 | 10419723 | 57269 | 1.31 | 0.0040 | 0.0060 |
| Olfr1510 | olfactory receptor 1510 | 10419717 | 258423 | 1.16 | 0.0028 | 0.0063 |
| Olfr325 | olfactory receptor 325 | 10376392 | 258261 | 1.28 | 0.0088 | 0.0060 |
| Olfr391 | olfactory receptor 391 | 10388286 | 258236 | 1.25 | 0.0016 | 0.0050 |
| Olfr722 | olfactory receptor 722 | 10419426 | 258487 | 1.14 | 0.0032 | 0.0055 |
| Orai3 | ORAI calcium release-activated calcium modulator 3 | 10557754 | 269999 | -1.10 | 0.0083 | 0.0103 |
| Odf3 | outer dense fiber of sperm tails 3 | 10558698 | 69287 | 1.17 | 0.0069 | 0.0050 |
| Ovgp1 | oviductal glycoprotein 1 | 10495120 | 12659 | 1.17 | 0.0038 | 0.0091 |
| Parvg | parvin, gamma | 10425866 | 64099 | -1.14 | 0.0032 | 0.0123 |
| Plcxd1 | phosphatidylinositol-specific phospholipase C, X domain containing 1 | 10524089 | 403178 | -1.13 | 0.0023 | 0.0181 |
| Pisd-ps1 | phosphatidylserine decarboxylase, pseudogene 1 | 10373702 | 236604 | 1.13 | 0.0013 | 0.0091 |
| Pik3cg | phosphoinositide-3-kinase, catalytic, gamma polypeptide | 10399924 | 30955 | -1.12 | 0.0084 | 0.0058 |
| Pdgfb | platelet derived growth factor, B polypeptide | 10430660 | 18591 | -1.12 | 0.0007 | 0.0088 |
| Pf4 | platelet factor 4 | 10523134 | 56744 | -1.15 | 0.0119 | 0.0112 |
| Pafah1b2 | platelet-activating factor acetylhydrolase, isoform 1b, subunit 2 | 10593159 | 18475 | -1.28 | 0.0127 | 0.0069 |
| Papolb | poly (A) polymerase beta (testis specific) | 10535329 | 56522 | 1.29 | 0.0022 | 0.0103 |
| Pcbp3 | poly(rC) binding protein 3 | 10370242 | 59093 | 1.23 | 0.0010 | 0.0165 |
| Polr2k | polymerase (RNA) II (DNA directed) polypeptide K | 10423742 | 17749 | -1.17 | 0.0100 | 0.0052 |
| Kctd1 | potassium channel tetramerisation domain containing 1 | 10457606 | 106931 | -1.11 | 0.0004 | 0.0203 |
| Kcnk4 | potassium channel, subfamily K, member 4 | 10465500 | 16528 | -1.12 | 0.0020 | 0.0077 |
| Kcnj12 | potassium inwardly-rectifying channel, subfamily J, member 12 | 10376747 | 16515 | -1.13 | 0.0032 | 0.0111 |
| Kcna5 | potassium voltage-gated channel, shaker-related subfamily, member 5 | 10548043 | 16493 | -1.18 | 0.0178 | 0.0212 |
| Kcng4 | potassium voltage-gated channel, subfamily G, member 4 | 10582146 | 66733 | -1.15 | 0.0033 | 0.0081 |
| Pou6f2 | POU domain, class 6, transcription factor 2 | 10407929 | 218030 | -1.10 | 0.0009 | 0.0257 |
| Pappa | pregnancy-associated plasma protein A | 10505489 | 18491 | 1.20 | 0.0123 | 0.0122 |
| Pawr | PRKC, apoptosis, WT1, regulator | 10366266 | 114774 | -1.14 | 0.0016 | 0.0111 |
| Prr15 | proline rich 15 | 10538373 | 78004 | -1.10 | 0.0010 | 0.0135 |
| Psme2 | proteasome (prosome, macropain) 28 subunit, beta | 10385511 | 19188 | -1.23 | 0.0117 | 0.0058 |
| P2ry1 | purinergic receptor P2Y, G-protein coupled 1 | 10492330 | 18441 | -1.15 | 0.0189 | 0.0133 |
| Pdk4 | pyruvate dehydrogenase kinase, isoenzyme 4 | 10543017 | 27273 | -1.19 | 0.0011 | 0.0063 |
| Pdp1 | pyruvate dehyrogenase phosphatase catalytic subunit 1 | 10511580 | 381511 | -1.11 | 0.0047 | 0.0231 |
| Rab37 | RAB37, member of RAS oncogene family | 10382449 | 58222 | 1.31 | 0.0003 | 0.0147 |
| Rdx | radixin | 10585318 | 19684 | 1.11 | 0.0054 | 0.0094 |
| Rassf1 | Ras association (RalGDS/AF-6) domain family member 1 | 10588669 | 56289 | -1.12 | 0.0063 | 0.0095 |
| Rgs14 | regulator of G-protein signaling 14 | 10405432 | 51791 | -1.14 | 0.0003 | 0.0067 |
| Rgs20 | regulator of G-protein signaling 20 | 10352957 | 58175 | -1.14 | 0.0069 | 0.0228 |
| Rarres2 | retinoic acid receptor responder (tazarotene induced) 2 | 10544573 | 71660 | -1.11 | 0.0052 | 0.0074 |
| Rdh12 | retinol dehydrogenase 12 | 10396840 | 77974 | -1.12 | 0.0104 | 0.0058 |
| Rltpr | RGD motif, leucine rich repeats, tropomodulin domain and proline-rich containing | 10574825 | 234695 | -1.10 | 0.0134 | 0.0123 |
| Arhgef15 | Rho guanine nucleotide exchange factor (GEF) 15 | 10387201 | 442801 | -1.12 | 0.0109 | 0.0163 |
| Rrp7a | ribosomal RNA processing 7 homolog A (S. cerevisiae) | 10430941 | 74778 | -1.13 | 0.0060 | 0.0085 |
| Rimbp3 | RIMS binding protein 3 | 10433971 | 239731 | -1.22 | 0.0017 | 0.0076 |
| Rnf113a1 | ring finger protein 113A1 | 10599243 | 69942 | -1.15 | 0.0167 | 0.0056 |
| Rnf213 | ring finger protein 213 | 10383233 | 672511 | 1.19 | 0.0033 | 0.0077 |
| Rbm8a | RNA binding motif protein 8a | 10494452 | 60365 | -1.13 | 0.0014 | 0.0081 |
| Rnu12 | RNA U12, small nuclear | 10425799 | 104307 | 1.12 | 0.0060 | 0.0124 |
| S100a6 | S100 calcium binding protein A6 (calcyclin) | 10493820 | 20200 | -1.14 | 0.0067 | 0.0089 |
| Sostdc1 | sclerostin domain containing 1 | 10395389 | 66042 | -1.16 | 0.0018 | 0.0360 |
| Sec24c | Sec24 related gene family, member C (S. cerevisiae) | 10417946 | 218811 | -1.15 | 0.0032 | 0.0074 |
| Serpinb1a | serine (or cysteine) peptidase inhibitor, clade B, member 1a | 10408557 | 66222 | -1.18 | 0.0022 | 0.0057 |
| Serpinb6b | serine (or cysteine) peptidase inhibitor, clade B, member 6b | 10404422 | 20708 | -1.17 | 0.0063 | 0.0066 |
| Shmt1 | serine hydroxymethyltransferase 1 (soluble) | 10386604 | 20425 | 1.12 | 0.0047 | 0.0090 |
| Spink10 | serine peptidase inhibitor, Kazal type 10 | 10456171 | 328971 | -1.12 | 0.0002 | 0.0067 |
| Setd6 | SET domain containing 6 | 10574404 | 66083 | -1.14 | 0.0070 | 0.0065 |
| Sh2d5 | SH2 domain containing 5 | 10509514 | 230863 | -1.11 | 0.0014 | 0.0127 |
| Slain2 | SLAIN motif family, member 2 | 10530560 | 75991 | -1.13 | 0.0170 | 0.0068 |
| Snhg1 | small nucleolar RNA host gene (non-protein coding) 1 | 10461156 | 83673 | 1.17 | 0.0026 | 0.0093 |
| Snord38a | small nucleolar RNA, C/D box 38A | 10515425 | 100217424 | 1.31 | 0.0135 | 0.0082 |
| Snora30 | small nucleolar RNA, H/ACA box 30 | 10557703 | 100217442 | 1.15 | 0.0031 | 0.0058 |
| Slc16a6 | solute carrier family 16 (monocarboxylic acid transporters), member 6 | 10392440 | 104681 | -1.10 | 0.0023 | 0.0065 |
| Slc29a1 | solute carrier family 29 (nucleoside transporters), member 1 | 10451123 | 63959 | -1.10 | 0.0125 | 0.0089 |
| Slc39a1 | solute carrier family 39 (zinc transporter), member 1 | 10493709 | 30791 | -1.12 | 0.0093 | 0.0064 |
| Slc43a3 | solute carrier family 43, member 3 | 10473384 | 58207 | 1.13 | 0.0038 | 0.0062 |
| Slco2a1 | solute carrier organic anion transporter family, member 2a1 | 10588263 | 24059 | -1.16 | 0.0009 | 0.0097 |
| Slco5a1 | solute carrier organic anion transporter family, member 5A1 | 10353117 | 240726 | 1.11 | 0.0043 | 0.0372 |
| Spata1 | spermatogenesis associated 1 | 10502696 | 70951 | -1.12 | 0.0194 | 0.0079 |
| St6galnac1 | ST6 (alpha-N-acetyl-neuraminyl-2,3-beta-galactosyl-1,3)-N-acetylgalactosaminide alpha-2,6-sialyltransferase 1 | 10393373 | 20445 | 1.17 | 0.0076 | 0.0034 |
| Stard6 | StAR-related lipid transfer (START) domain containing 6 | 10363181 | 170461 | -1.10 | 0.0042 | 0.0065 |
| Stard9 | START domain containing 9 | 10475226 | 668880 | 1.14 | 0.0127 | 0.0084 |
| Sdf2l1 | stromal cell-derived factor 2-like 1 | 10438098 | 64136 | -1.16 | 0.0030 | 0.0038 |
| Sftpc | surfactant associated protein C | 10421387 | 20389 | -1.19 | 0.0192 | 0.0060 |
| Syt10 | synaptotagmin X | 10431625 | 54526 | 1.24 | 0.0014 | 0.0096 |
| Tgtp1 | T-cell specific GTPase 1 | 10385533 | 21822 | -1.20 | 0.0145 | 0.0087 |
| Tcte3 | t-complex-associated testis expressed 3 | 10448023 | 21647 | -1.11 | 0.0014 | 0.0096 |
| Tatdn2 | TatD DNase domain containing 2 | 10540812 | 381801 | -1.11 | 0.0054 | 0.0082 |
| Terc | telomerase RNA component | 10500345 | 21748 | -1.16 | 0.0015 | 0.0077 |
| Tesc | tescalcin | 10524955 | 57816 | -1.15 | 0.0047 | 0.0087 |
| Thsd7b | thrombospondin, type I, domain containing 7B | 10349529 | 210417 | 1.14 | 0.0053 | 0.0113 |
| Sp8 | trans-acting transcription factor 8 | 10399189 | 320145 | 1.39 | 0.0023 | 0.0267 |
| Tceal5 | transcription elongation factor A (SII)-like 5 | 10606864 | 331532 | -1.12 | 0.0047 | 0.0103 |
| Tle2 | transducin-like enhancer of split 2, homolog of Drosophila E(spl) | 10365145 | 21886 | 1.13 | 0.0015 | 0.0118 |
| Trpc6 | transient receptor potential cation channel, subfamily C, member 6 | 10583163 | 22068 | -1.16 | 0.0098 | 0.0092 |
| Tm4sf1 | transmembrane 4 superfamily member 1 | 10498273 | 17112 | -1.12 | 0.0073 | 0.0086 |
| Tmcc3 | transmembrane and coiled coil domains 3 | 10365891 | 319880 | -1.12 | 0.0052 | 0.0090 |
| Tmem186 | transmembrane protein 186 | 10437586 | 66690 | -1.12 | 0.0012 | 0.0093 |
| Tmem97 | transmembrane protein 97 | 10434668 | 69071 | -1.11 | 0.0020 | 0.0091 |
| Tnnc1 | troponin C, cardiac/slow skeletal | 10413726 | 21924 | -1.19 | 0.0091 | 0.0481 |
| Tsc22d3 | TSC22 domain family, member 3 | 10606989 | 14605 | -1.21 | 0.0237 | 0.0056 |
| Tulp2 | tubby-like protein 2 | 10552966 | 56734 | 1.19 | 0.0068 | 0.0067 |
| Ttll8 | tubulin tyrosine ligase-like family, member 8 | 10431311 | 239591 | 1.21 | 0.0009 | 0.0097 |
| Tnfrsf13c | tumor necrosis factor receptor superfamily, member 13c | 10430818 | 72049 | 1.16 | 0.0044 | 0.0097 |
| Tnfrsf23 | tumor necrosis factor receptor superfamily, member 23 | 10569504 | 79201 | 1.24 | 0.0107 | 0.0117 |
| Ubc | ubiquitin C | 10525885 | 22190 | -1.10 | 0.0012 | 0.0100 |
| Ubr1 | ubiquitin protein ligase E3 component n-recognin 1 | 10475245 | 22222 | -1.11 | 0.0024 | 0.0073 |
| Ugt2b38 | UDP glucuronosyltransferase 2 family, polypeptide B38 | 10531073 | 100559 | 1.13 | 0.0035 | 0.0050 |
| Upp1 | uridine phosphorylase 1 | 10374236 | 22271 | -1.12 | 0.0004 | 0.0036 |
| Vps24 | vacuolar protein sorting 24 (yeast) | 10453692 | 66700 | -1.17 | 0.0060 | 0.0033 |
| Vipr1 | vasoactive intestinal peptide receptor 1 | 10590381 | 22354 | -1.17 | 0.0041 | 0.0159 |
| Vipr2 | vasoactive intestinal peptide receptor 2 | 10399046 | 22355 | 1.65 | 0.0036 | 0.1182 |
| Vmn1r213 | vomeronasal 1 receptor 213 | 10404008 | 171249 | 1.23 | 0.0096 | 0.0092 |
| Vmn1r7 | vomeronasal 1 receptor 7 | 10544993 | 434016 | 1.40 | 0.0204 | 0.0068 |
| Vmn2r54 | vomeronasal 2, receptor 54 | 10560028 | 666085 | 1.21 | 0.0010 | 0.0059 |
| Vwa3b | von Willebrand factor A domain containing 3B | 10345554 | 70853 | -1.13 | 0.0099 | 0.0468 |
| Wfdc1 | WAP four-disulfide core domain 1 | 10575917 | 67866 | -1.22 | 0.0046 | 0.0080 |
| Wdr62 | WD repeat domain 62 | 10551848 | 233064 | -1.24 | 0.0200 | 0.0037 |
| B4galt7 | xylosylprotein beta1,4-galactosyltransferase, polypeptide 7 (galactosyltransferase I) | 10405495 | 218271 | -1.12 | 0.0011 | 0.0081 |
| Zfp330 | zinc finger protein 330 | 10579969 | 30932 | -1.13 | 0.0020 | 0.0074 |
| Zfp367 | zinc finger protein 367 | 10410092 | 238673 | -1.17 | 0.0139 | 0.0126 |
| Zfp459 | zinc finger protein 459 | 10410317 | 328274 | -1.16 | 0.0178 | 0.0089 |
| Zfp608 | zinc finger protein 608 | 10458940 | 269023 | 1.14 | 0.0077 | 0.0074 |
| Zfp628 | zinc finger protein 628 | 10549723 | 232816 | -1.15 | 0.0057 | 0.0049 |
| Zfp777 | zinc finger protein 777 | 10544547 | 72306 | -1.15 | 0.0064 | 0.0098 |
| Zdbf2 | zinc finger, DBF-type containing 2 | 10346878 | 73884 | -1.14 | 0.0112 | 0.0090 |

**Table 2**

A total number of 1,459 frontal cortical genes were included in OPLS-DA analysis. The 856 genes that significantly contributed to group separation are listed.

^a^The Entrez ID of genes; ^b^Fold change in gene expression in allergic mice brain with regard to controls;

^c^Subtraction of absolute (Abs) values of jack-knife confidence interval (JKCI) from Abs values of Loadings derived from the cross-validation of the OPLS-DA model. Positive values indicate significance. ^d^p-values calculated by using student´s t-test.

| Gene symbol | Gene name | Probeset | Entrez^a^ | F-C^b^ | Sign^c^ | p-Values^d^ |
| --- | --- | --- | --- | --- | --- | --- |
| Bdh2 | 3-hydroxybutyrate dehydrogenase, type 2 | 10496251 | 69772 | 1.19 | 0.0100 | 0.0924 |
| Htr2c | 5-hydroxytryptamine (serotonin) receptor 2C | 10602261 | 15560 | 1.47 | 0.0120 | 0.0590 |
| Nt5dc1 | 5'-nucleotidase domain containing 1 | 10368654 | 319638 | 1.20 | 0.0150 | 0.1345 |
| Adam17 | a disintegrin and metallopeptidase domain 17 | 10399605 | 11491 | 1.17 | 0.0042 | 0.1618 |
| Adam4 | a disintegrin and metallopeptidase domain 4 | 10401320 | 11498 | 1.19 | 0.0034 | 0.0958 |
| Abhd14b | abhydrolase domain containing 14b | 10588505 | 76491 | 1.26 | 0.0161 | 0.0493 |
| Abhd2 | abhydrolase domain containing 2 | 10554269 | 54608 | 1.24 | 0.0085 | 0.1278 |
| Acaa2 | acetyl-Coenzyme A acyltransferase 2 (mitochondrial 3-oxoacyl-Coenzyme A thiolase) | 10456699 | 52538 | 1.24 | 0.0081 | 0.1433 |
| Acacb | acetyl-Coenzyme A carboxylase beta | 10524460 | 100705 | 1.35 | 0.0140 | 0.0349 |
| Acss3 | acyl-CoA synthetase short-chain family member 3 | 10372208 | 380660 | 1.26 | 0.0102 | 0.1060 |
| Acad10 | acyl-Coenzyme A dehydrogenase family, member 10 | 10533367 | 71985 | 1.19 | 0.0065 | 0.0937 |
| Acad8 | acyl-Coenzyme A dehydrogenase family, member 8 | 10591947 | 66948 | 1.17 | 0.0056 | 0.1680 |
| Acadl | acyl-Coenzyme A dehydrogenase, long-chain | 10355246 | 11363 | 1.17 | 0.0084 | 0.1624 |
| Adamtsl2 | ADAMTS-like 2 | 10470392 | 77794 | 1.18 | 0.0019 | 0.1522 |
| Akd1 | adenylate kinase domain containing 1 | 10362729 | 633979 | 1.42 | 0.0070 | 0.0759 |
| Arl4d | ADP-ribosylation factor-like 4D | 10381474 | 80981 | 1.17 | 0.0035 | 0.1305 |
| Adrb2 | adrenergic receptor, beta 2 | 10459288 | 11555 | 1.18 | 0.0025 | 0.0741 |
| Aebp1 | AE binding protein 1 | 10374083 | 11568 | 1.30 | 0.0132 | 0.0581 |
| Ahnak | AHNAK nucleoprotein (desmoyokin) | 10461369 | 66395 | 1.27 | 0.0183 | 0.0178 |
| Aard | alanine and arginine rich domain containing protein | 10424082 | 239435 | 1.17 | 0.0005 | 0.0521 |
| Anpep | alanyl (membrane) aminopeptidase | 10564818 | 16790 | 1.20 | 0.0105 | 0.1054 |
| Adh1 | alcohol dehydrogenase 1 (class I) | 10496438 | 11522 | 1.30 | 0.0158 | 0.0226 |
| Aldh3b2 | aldehyde dehydrogenase 3 family, member B2 | 10460253 | 621603 | 1.23 | 0.0039 | 0.0640 |
| Aox1 | aldehyde oxidase 1 | 10346374 | 11761 | 1.30 | 0.0198 | 0.0461 |
| Aox3 | aldehyde oxidase 3 | 10346410 | 71724 | 1.24 | 0.0203 | 0.0318 |
| Alpl | alkaline phosphatase, liver/bone/kidney | 10517587 | 11647 | 1.30 | 0.0255 | 0.0089 |
| A2m | alpha-2-macroglobulin | 10541354 | 232345 | 1.69 | 0.0060 | 0.0342 |
| Alas2 | aminolevulinic acid synthase 2, erythroid | 10602372 | 11656 | 1.54 | 0.0042 | 0.0201 |
| Amy2a5 | amylase 2a5 | 10501494 | 109959 | 1.17 | 0.0022 | 0.1025 |
| Als2cr4 | amyotrophic lateral sclerosis 2 (juvenile) chromosome region, candidate 4 | 10354938 | 381259 | 1.24 | 0.0132 | 0.1029 |
| Angel1 | angel homolog 1 (Drosophila) | 10401684 | 68737 | 1.17 | 0.0087 | 0.0799 |
| Angptl2 | angiopoietin-like 2 | 10471555 | 26360 | 1.56 | 0.0070 | 0.0225 |
| Ace | angiotensin I converting enzyme (peptidyl-dipeptidase A) 1 | 10381962 | 11421 | 1.78 | 0.0021 | 0.0556 |
| Agtrap | angiotensin II, type I receptor-associated protein | 10518455 | 11610 | 1.36 | 0.0310 | 0.0081 |
| Ankrd23 | ankyrin repeat domain 23 | 10353878 | 78321 | 1.18 | 0.0037 | 0.1112 |
| Ankrd33b | ankyrin repeat domain 33B | 10428002 | 67434 | 1.24 | 0.0039 | 0.0267 |
| Ankrd57 | ankyrin repeat domain 57 | 10363346 | 268301 | 1.29 | 0.0148 | 0.0216 |
| Anxa2 | annexin A2 | 10586744 | 12306 | 1.27 | 0.0050 | 0.1257 |
| Anxa4 | annexin A4 | 10545958 | 11746 | 1.18 | 0.0119 | 0.1213 |
| Antxr1 | anthrax toxin receptor 1 | 10545974 | 69538 | 1.26 | 0.0187 | 0.0554 |
| Airn | antisense Igf2r RNA | 10441787 | 104103 | 1.23 | 0.0064 | 0.0832 |
| Apoc1 | apolipoprotein C-I | 10560618 | 11812 | 1.21 | 0.0102 | 0.0712 |
| Apod | apolipoprotein D | 10439009 | 11815 | 1.21 | 0.0026 | 0.1169 |
| Aifm2 | apoptosis-inducing factor, mitochondrion-associated 2 | 10363528 | 71361 | 1.20 | 0.0050 | 0.0639 |
| Aqp1 | aquaporin 1 | 10538459 | 11826 | 1.84 | 0.0067 | 0.0581 |
| Armc2 | armadillo repeat containing 2 | 10368877 | 213402 | 1.18 | 0.0056 | 0.0612 |
| Arvcf | armadillo repeat gene deleted in velo-cardio-facial syndrome | 10434165 | 11877 | 1.17 | 0.0040 | 0.1091 |
| Arrdc2 | arrestin domain containing 2 | 10579406 | 70807 | 1.17 | 0.0098 | 0.0692 |
| Arsg | arylsulfatase G | 10382271 | 74008 | 1.18 | 0.0127 | 0.1551 |
| Asprv1 | aspartic peptidase, retroviral-like 1 | 10539739 | 67855 | 1.24 | 0.0009 | 0.0336 |
| Acy3 | aspartoacylase (aminoacylase) 3 | 10460263 | 71670 | 1.17 | 0.0056 | 0.0309 |
| Aga | aspartylglucosaminidase | 10571774 | 11593 | 1.21 | 0.0124 | 0.0951 |
| Atoh8 | atonal homolog 8 (Drosophila) | 10545372 | 71093 | 1.26 | 0.0218 | 0.0182 |
| Abca4 | ATP-binding cassette, sub-family A (ABC1), member 4 | 10495712 | 11304 | 1.82 | 0.0027 | 0.0721 |
| Abcb1a | ATP-binding cassette, sub-family B (MDR/TAP), member 1A | 10519527 | 18671 | 1.23 | 0.0052 | 0.0194 |
| Abcc1 | ATP-binding cassette, sub-family C (CFTR/MRP), member 1 | 10433735 | 17250 | 1.16 | 0.0106 | 0.1028 |
| Abcc12 | ATP-binding cassette, sub-family C (CFTR/MRP), member 12 | 10580418 | 244562 | 1.21 | 0.0050 | 0.1039 |
| Abcc4 | ATP-binding cassette, sub-family C (CFTR/MRP), member 4 | 10422280 | 239273 | 1.22 | 0.0108 | 0.1189 |
| Agbl2 | ATP/GTP binding protein-like 2 | 10473708 | 271813 | 1.28 | 0.0014 | 0.2155 |
| Atp13a5 | ATPase type 13A5 | 10438822 | 268878 | 1.17 | 0.0016 | 0.0626 |
| Atp2a3 | ATPase, Ca++ transporting, ubiquitous | 10378216 | 53313 | 1.16 | 0.0091 | 0.0446 |
| Atp8b1 | ATPase, class I, type 8B, member 1 | 10459421 | 54670 | 1.15 | 0.0017 | 0.1467 |
| Atp10d | ATPase, class V, type 10D | 10522335 | 231287 | 1.26 | 0.0179 | 0.0449 |
| Atp11c | ATPase, class VI, type 11C | 10604799 | 320940 | 1.27 | 0.0203 | 0.0498 |
| Atp7a | ATPase, Cu++ transporting, alpha polypeptide | 10601360 | 11977 | 1.19 | 0.0074 | 0.2177 |
| Atp6v0e | ATPase, H+ transporting, lysosomal V0 subunit E | 10443021 | 11974 | 1.18 | 0.0062 | 0.1836 |
| Bcl2 | B-cell leukemia/lymphoma 2 | 10357043 | 12043 | 1.15 | 0.0068 | 0.1730 |
| Baiap2l1 | BAI1-associated protein 2-like 1 | 10535559 | 66898 | 1.56 | 0.0042 | 0.0578 |
| Bcam | basal cell adhesion molecule | 10560655 | 57278 | 1.27 | 0.0063 | 0.0696 |
| Batf | basic leucine zipper transcription factor, ATF-like | 10397359 | 53314 | 1.26 | 0.0029 | 0.0726 |
| Batf2 | basic leucine zipper transcription factor, ATF-like 2 | 10460767 | 74481 | 1.23 | 0.0119 | 0.0846 |
| Batf3 | basic leucine zipper transcription factor, ATF-like 3 | 10352717 | 381319 | 1.17 | 0.0074 | 0.0339 |
| Best3 | bestrophin 3 | 10366528 | 382427 | 1.25 | 0.0067 | 0.1269 |
| Bhmt | betaine-homocysteine methyltransferase | 10457114 | 12116 | 1.21 | 0.0185 | 0.0517 |
| Bgn | biglycan | 10600169 | 12111 | 1.18 | 0.0069 | 0.1268 |
| Btd | biotinidase | 10413803 | 26363 | 1.18 | 0.0028 | 0.2086 |
| Bst2 | bone marrow stromal cell antigen 2 | 10579532 | 69550 | 1.22 | 0.0043 | 0.0260 |
| Bmp4 | bone morphogenetic protein 4 | 10419261 | 12159 | 1.32 | 0.0126 | 0.0169 |
| Bmp5 | bone morphogenetic protein 5 | 10587231 | 12160 | 1.16 | 0.0130 | 0.1074 |
| Bmp6 | bone morphogenetic protein 6 | 10404686 | 12161 | 1.52 | 0.0048 | 0.0532 |
| Bmp7 | bone morphogenetic protein 7 | 10490129 | 12162 | 1.47 | 0.0055 | 0.0262 |
| Bdkrb1 | bradykinin receptor, beta 1 | 10398121 | 12061 | 1.20 | 0.0042 | 0.0749 |
| Bcas1 | breast carcinoma amplified sequence 1 | 10490061 | 76960 | 1.16 | 0.0071 | 0.1502 |
| Bin2 | bridging integrator 2 | 10432640 | 668218 | 1.18 | 0.0188 | 0.0429 |
| Baz1a | bromodomain adjacent to zinc finger domain 1A | 10400357 | 217578 | 1.23 | 0.0043 | 0.0040 |
| Clec2j | C-type lectin domain family 2, member J | 10548295 | 677440 | 1.40 | 0.0053 | 0.0324 |
| C1qtnf1 | C1q and tumor necrosis factor related protein 1 | 10383025 | 56745 | 1.16 | 0.0138 | 0.0988 |
| C1qtnf5 | C1q and tumor necrosis factor related protein 5 | 10584653 | 235312 | 1.63 | 0.0057 | 0.0538 |
| Cdh1 | cadherin 1 | 10575052 | 12550 | 1.20 | 0.0041 | 0.1497 |
| Cdh18 | cadherin 18 | 10423271 | 320865 | 1.17 | 0.0015 | 0.1054 |
| Cdh3 | cadherin 3 | 10575034 | 12560 | 1.39 | 0.0087 | 0.0244 |
| Cab39l | calcium binding protein 39-like | 10415678 | 69008 | 1.34 | 0.0090 | 0.0742 |
| Cacnb2 | calcium channel, voltage-dependent, beta 2 subunit | 10480254 | 12296 | 1.15 | 0.0038 | 0.1013 |
| Capsl | calcyphosine-like | 10423024 | 75568 | 1.25 | 0.0001 | 0.1509 |
| Calml4 | calmodulin-like 4 | 10586118 | 75600 | 1.56 | 0.0053 | 0.0978 |
| Capn6 | calpain 6 | 10607143 | 12338 | 1.25 | 0.0210 | 0.0148 |
| Cast | calpastatin | 10410656 | 12380 | 1.19 | 0.0153 | 0.0562 |
| Cnn2 | calponin 2 | 10364593 | 12798 | 1.23 | 0.0085 | 0.0580 |
| Cml3 | camello-like 3 | 10545862 | 93674 | 1.22 | 0.0160 | 0.0942 |
| Cml5 | camello-like 5 | 10545874 | 69049 | 1.17 | 0.0078 | 0.1359 |
| Chst14 | carbohydrate (N-acetylgalactosamine 4-0) sulfotransferase 14 | 10474860 | 72136 | 1.22 | 0.0141 | 0.0332 |
| Car14 | carbonic anhydrase 14 | 10500283 | 23831 | 1.48 | 0.0099 | 0.0553 |
| Car12 | carbonic anyhydrase 12 | 10586591 | 76459 | 1.38 | 0.0101 | 0.0793 |
| Ces5 | carboxylesterase 5 | 10574545 | 234673 | 1.17 | 0.0103 | 0.0640 |
| Cpt1a | carnitine palmitoyltransferase 1a, liver | 10460157 | 12894 | 1.18 | 0.0059 | 0.2169 |
| Cpt2 | carnitine palmitoyltransferase 2 | 10514933 | 12896 | 1.16 | 0.0012 | 0.2567 |
| Cndp1 | carnosine dipeptidase 1 (metallopeptidase M20 family) | 10460072 | 338403 | 1.24 | 0.0184 | 0.0535 |
| Carns1 | carnosine synthase 1 | 10464642 | 107239 | 1.15 | 0.0164 | 0.0772 |
| Crtap | cartilage associated protein | 10597413 | 56693 | 1.28 | 0.0192 | 0.0353 |
| Cass4 | Cas scaffolding protein family member 4 | 10490116 | 320664 | 1.15 | 0.0004 | 0.1918 |
| Casp8 | caspase 8 | 10346564 | 12370 | 1.30 | 0.0163 | 0.0766 |
| Ctnnal1 | catenin (cadherin associated protein), alpha-like 1 | 10513061 | 54366 | 1.31 | 0.0159 | 0.0608 |
| Ctsd | cathepsin D | 10569319 | 13033 | 1.17 | 0.0032 | 0.2356 |
| Ctsh | cathepsin H | 10587733 | 13036 | 1.24 | 0.0096 | 0.0967 |
| Ctsm | cathepsin M | 10409943 | 64139 | 1.18 | 0.0021 | 0.1843 |
| Cd24a | CD24a antigen | 10362896 | 12484 | 1.22 | 0.0119 | 0.0907 |
| Cd274 | CD274 antigen | 10462390 | 60533 | 1.18 | 0.0002 | 0.1562 |
| Cd55 | CD55 antigen | 10357488 | 13136 | 1.31 | 0.0069 | 0.0827 |
| Cd59a | CD59a antigen | 10474229 | 12509 | 1.54 | 0.0006 | 0.0901 |
| Cd59b | CD59b antigen | 10474223 | 333883 | 1.27 | 0.0097 | 0.0675 |
| Cd6 | CD6 antigen | 10466054 | 12511 | 1.17 | 0.0084 | 0.0147 |
| Cd63 | CD63 antigen | 10367436 | 12512 | 1.22 | 0.0068 | 0.1409 |
| Cd74 | CD74 antigen (invariant polypeptide of major histocompatibility complex, class II antigen-associated) | 10456005 | 16149 | 1.28 | 0.0002 | 0.1859 |
| Cd82 | CD82 antigen | 10485213 | 12521 | 1.27 | 0.0053 | 0.1611 |
| Cd9 | CD9 antigen | 10548030 | 12527 | 1.17 | 0.0030 | 0.2133 |
| Cdc14b | CDC14 cell division cycle 14 homolog B (S. cerevisiae) | 10410099 | 218294 | 1.18 | 0.0129 | 0.1499 |
| Cks2 | CDC28 protein kinase regulatory subunit 2 | 10405185 | 66197 | 1.18 | 0.0057 | 0.1546 |
| Cdc42bpg | CDC42 binding protein kinase gamma (DMPK-like) | 10460841 | 240505 | 1.16 | 0.0140 | 0.0613 |
| Cdc42ep3 | CDC42 effector protein (Rho GTPase binding) 3 | 10453049 | 260409 | 1.21 | 0.0074 | 0.1380 |
| Crabp1 | cellular retinoic acid binding protein I | 10585438 | 12903 | 1.41 | 0.0050 | 0.0707 |
| Cenpj | centromere protein J | 10420320 | 219103 | 1.22 | 0.0028 | 0.0667 |
| Cep110 | centrosomal protein 110 | 10471608 | 26920 | 1.18 | 0.0036 | 0.1162 |
| Cep290 | centrosomal protein 290 | 10366073 | 216274 | 1.15 | 0.0017 | 0.1871 |
| Cep63 | centrosomal protein 63 | 10596117 | 28135 | 1.16 | 0.0000 | 0.1442 |
| Cdr2 | cerebellar degeneration-related 2 | 10567564 | 12585 | 1.23 | 0.0069 | 0.1823 |
| Cbln4 | cerebellin 4 precursor protein | 10490097 | 228942 | -1.17 | 0.0104 | 0.2176 |
| Ccl25 | chemokine (C-C motif) ligand 25 | 10569962 | 20300 | 1.18 | 0.0131 | 0.1044 |
| Chia | chitinase, acidic | 10495136 | 81600 | 1.30 | 0.0030 | 0.0732 |
| Clic3 | chloride intracellular channel 3 | 10470101 | 69454 | 1.31 | 0.0114 | 0.0811 |
| Clic6 | chloride intracellular channel 6 | 10436958 | 209195 | 1.62 | 0.0006 | 0.0980 |
| Chdh | choline dehydrogenase | 10413517 | 218865 | 1.19 | 0.0098 | 0.0954 |
| Chrnb4 | cholinergic receptor, nicotinic, beta polypeptide 4 | 10593767 | 108015 | 1.88 | 0.0047 | 0.0587 |
| Chrdl1 | chordin-like 1 | 10607124 | 83453 | 1.18 | 0.0034 | 0.2460 |
| Cgnl1 | cingulin-like 1 | 10594855 | 68178 | 1.50 | 0.0119 | 0.0334 |
| Cmtm7 | CKLF-like MARVEL transmembrane domain containing 7 | 10597461 | 102545 | 1.22 | 0.0099 | 0.1107 |
| Cmtm8 | CKLF-like MARVEL transmembrane domain containing 8 | 10597470 | 70031 | 1.31 | 0.0037 | 0.0828 |
| Cldn2 | claudin 2 | 10602033 | 12738 | 1.98 | 0.0017 | 0.0541 |
| Cnksr3 | Cnksr family member 3 | 10361323 | 215748 | 1.24 | 0.0127 | 0.0459 |
| F7 | coagulation factor VII | 10570280 | 14068 | 1.22 | 0.0053 | 0.0743 |
| Cchcr1 | coiled-coil alpha-helical rod protein 1 | 10444858 | 240084 | 1.19 | 0.0041 | 0.0946 |
| Ccdc108 | coiled-coil domain containing 108 | 10355670 | 241116 | 1.56 | 0.0140 | 0.0251 |
| Ccdc113 | coiled-coil domain containing 113 | 10574365 | 244608 | 1.22 | 0.0216 | 0.0794 |
| Ccdc114 | coiled-coil domain containing 114 | 10553148 | 211535 | 1.29 | 0.0123 | 0.0713 |
| Ccdc135 | coiled-coil domain containing 135 | 10574288 | 330830 | 1.68 | 0.0055 | 0.0720 |
| Ccdc138 | coiled-coil domain containing 138 | 10363312 | 76138 | 1.15 | 0.0056 | 0.1105 |
| Ccdc141 | coiled-coil domain containing 141 | 10484197 | 545428 | 1.22 | 0.0085 | 0.1377 |
| Ccdc157 | coiled-coil domain containing 157 | 10383833 | 216516 | 1.30 | 0.0231 | 0.0203 |
| Ccdc33 | coiled-coil domain containing 33 | 10594015 | 382077 | 1.15 | 0.0017 | 0.1340 |
| Ccdc60 | coiled-coil domain containing 60 | 10533034 | 269693 | 1.39 | 0.0155 | 0.0410 |
| Ccdc88b | coiled-coil domain containing 88B | 10465446 | 78317 | 1.19 | 0.0019 | 0.0560 |
| Col1a1 | collagen, type I, alpha 1 | 10380419 | 12842 | 1.17 | 0.0125 | 0.0690 |
| Col9a3 | collagen, type IX, alpha 3 | 10479423 | 12841 | 1.37 | 0.0046 | 0.0050 |
| Col5a1 | collagen, type V, alpha 1 | 10481259 | 12831 | 1.23 | 0.0153 | 0.0214 |
| Col6a1 | collagen, type VI, alpha 1 | 10370210 | 12833 | 1.22 | 0.0047 | 0.0606 |
| Col6a3 | collagen, type VI, alpha 3 | 10356520 | 12835 | 1.22 | 0.0010 | 0.1845 |
| Col6a6 | collagen, type VI, alpha 6 | 10596383 | 245026 | 1.24 | 0.0037 | 0.0617 |
| Col8a1 | collagen, type VIII, alpha 1 | 10440091 | 12837 | 2.13 | 0.0068 | 0.0328 |
| Col12a1 | collagen, type XII, alpha 1 | 10595211 | 12816 | 1.20 | 0.0104 | 0.0592 |
| Col14a1 | collagen, type XIV, alpha 1 | 10424140 | 12818 | 1.18 | 0.0136 | 0.0511 |
| Col18a1 | collagen, type XVIII, alpha 1 | 10370259 | 12822 | 1.17 | 0.0133 | 0.0157 |
| Col27a1 | collagen, type XXVII, alpha 1 | 10505374 | 373864 | 1.15 | 0.0014 | 0.1123 |
| Colec12 | collectin sub-family member 12 | 10453747 | 140792 | 1.17 | 0.0059 | 0.2284 |
| C1rl | complement component 1, r subcomponent-like | 10541670 | 232371 | 1.19 | 0.0022 | 0.0647 |
| C4b | complement component 4B (Childo blood group) | 10450242 | 12268 | 1.26 | 0.0048 | 0.0487 |
| Cfh | complement component factor h | 10358339 | 12628 | 1.21 | 0.0076 | 0.1428 |
| Cplx3 | complexin 3 | 10593947 | 235415 | 1.17 | 0.0078 | 0.1391 |
| Cdan1 | congenital dyserythropoietic anemia, type I (human) | 10475229 | 68968 | 1.23 | 0.0040 | 0.0193 |
| Cops3 | COP9 (constitutive photomorphogenic) homolog, subunit 3 (Arabidopsis thaliana) | 10376555 | 26572 | 1.18 | 0.0168 | 0.0705 |
| Crhr2 | corticotropin releasing hormone receptor 2 | 10544913 | 12922 | 1.40 | 0.0014 | 0.0261 |
| Cxadr | coxsackie virus and adenovirus receptor | 10436608 | 13052 | 1.19 | 0.0150 | 0.0592 |
| Crb2 | crumbs homolog 2 (Drosophila) | 10471819 | 241324 | 1.21 | 0.0102 | 0.1291 |
| Crybb1 | crystallin, beta B1 | 10524338 | 12960 | 1.23 | 0.0123 | 0.0714 |
| Cdkn1c | cyclin-dependent kinase inhibitor 1C (P57) | 10569429 | 12577 | 1.30 | 0.0124 | 0.0479 |
| Cst10 | cystatin 10 (chondrocytes) | 10476931 | 58214 | 1.23 | 0.0051 | 0.0372 |
| Cst9 | cystatin 9 | 10476928 | 13013 | 1.21 | 0.0075 | 0.1097 |
| Csrp2 | cysteine and glycine-rich protein 2 | 10366293 | 13008 | 1.39 | 0.0097 | 0.0747 |
| Cyyr1 | cysteine and tyrosine-rich protein 1 | 10440513 | 224405 | 1.31 | 0.0058 | 0.0024 |
| Cox6b2 | cytochrome c oxidase subunit VIb polypeptide 2 | 10559649 | 333182 | 1.30 | 0.0012 | 0.0528 |
| Cox7a1 | cytochrome c oxidase, subunit VIIa 1 | 10551836 | 12865 | 1.18 | 0.0063 | 0.1780 |
| Cox8b | cytochrome c oxidase, subunit VIIIb | 10569008 | 12869 | 1.59 | 0.0039 | 0.1072 |
| Cyp4f13 | cytochrome P450, family 4, subfamily f, polypeptide 13 | 10449955 | 170716 | 1.21 | 0.0206 | 0.0555 |
| Cish | cytokine inducible SH2-containing protein | 10588577 | 12700 | 1.23 | 0.0158 | 0.0175 |
| Dock6 | dedicator of cytokinesis 6 | 10591576 | 319899 | 1.37 | 0.0227 | 0.0029 |
| Degs2 | degenerative spermatocyte homolog 2 (Drosophila), lipid desaturase | 10402575 | 70059 | 1.24 | 0.0107 | 0.0117 |
| Dhrs11 | dehydrogenase/reductase (SDR family) member 11 | 10389300 | 192970 | 1.22 | 0.0135 | 0.0488 |
| Dhrs13 | dehydrogenase/reductase (SDR family) member 13 | 10379006 | 70451 | 1.16 | 0.0082 | 0.1166 |
| Dlec1 | deleted in lung and esophageal cancer 1 | 10590096 | 320256 | 1.26 | 0.0113 | 0.0834 |
| Dnase2a | deoxyribonuclease II alpha | 10573461 | 13423 | 1.21 | 0.0162 | 0.1007 |
| Dsg2 | desmoglein 2 | 10454172 | 13511 | 1.26 | 0.0062 | 0.1361 |
| Def6 | differentially expressed in FDCP 6 | 10443319 | 23853 | 1.17 | 0.0008 | 0.0271 |
| Dab2 | disabled homolog 2 (Drosophila) | 10422728 | 13132 | 1.31 | 0.0072 | 0.0769 |
| Ddr1 | discoidin domain receptor family, member 1 | 10450579 | 12305 | 1.17 | 0.0072 | 0.1406 |
| Ddr2 | discoidin domain receptor family, member 2 | 10359929 | 18214 | 1.19 | 0.0074 | 0.1034 |
| Dlx1as | distal-less homeobox 1, antisense | 10483624 | 111970 | 1.20 | 0.0084 | 0.0908 |
| Dram1 | DNA-damage regulated autophagy modulator 1 | 10371607 | 71712 | 1.22 | 0.0110 | 0.0810 |
| Dnajb13 | DnaJ (Hsp40) related, subfamily B, member 13 | 10565890 | 69387 | 1.20 | 0.0054 | 0.1131 |
| Dct | dopachrome tautomerase | 10422249 | 13190 | 1.31 | 0.0088 | 0.0530 |
| Dcdc5 | doublecortin domain containing 5 | 10474355 | 329482 | 1.22 | 0.0041 | 0.1059 |
| Dmrt3 | doublesex and mab-3 related transcription factor 3 | 10462228 | 240590 | 1.19 | 0.0015 | 0.1394 |
| Dusp15 | dual specificity phosphatase-like 15 | 10488678 | 252864 | 1.19 | 0.0013 | 0.1171 |
| Dnahc10 | dynein, axonemal, heavy chain 10 | 10525858 | 56087 | 1.21 | 0.0008 | 0.2253 |
| Dnahc11 | dynein, axonemal, heavy chain 11 | 10403112 | 13411 | 1.42 | 0.0055 | 0.0981 |
| Dnahc2 | dynein, axonemal, heavy chain 2 | 10387397 | 327954 | 1.27 | 0.0123 | 0.0610 |
| Dnahc6 | dynein, axonemal, heavy chain 6 | 10545515 | 330355 | 1.41 | 0.0020 | 0.1453 |
| Dnahc7b | dynein, axonemal, heavy chain 7B | 10354576 | 227058 | 1.38 | 0.0095 | 0.0288 |
| Dmpk | dystrophia myotonica-protein kinase | 10550574 | 13400 | 1.21 | 0.0078 | 0.0682 |
| Elf4 | E74-like factor 4 (ets domain transcription factor) | 10604393 | 56501 | 1.27 | 0.0120 | 0.0153 |
| Ebf1 | early B-cell factor 1 | 10375360 | 13591 | 1.27 | 0.0139 | 0.0127 |
| Ebf2 | early B-cell factor 2 | 10421026 | 13592 | 1.15 | 0.0082 | 0.1110 |
| Enpp7 | ectonucleotide pyrophosphatase/phosphodiesterase 7 | 10383047 | 238011 | 1.19 | 0.0059 | 0.1518 |
| Edaradd | EDAR (ectodysplasin-A receptor)-associated death domain | 10407782 | 171211 | 1.16 | 0.0083 | 0.1127 |
| Efhc1 | EF-hand domain (C-terminal) containing 1 | 10345046 | 71877 | 1.26 | 0.0039 | 0.1350 |
| Efhc2 | EF-hand domain (C-terminal) containing 2 | 10603768 | 74405 | 1.16 | 0.0142 | 0.2044 |
| Egfl8 | EGF-like domain 8 | 10450212 | 81701 | 1.19 | 0.0107 | 0.0622 |
| Egln3 | EGL nine homolog 3 (C. elegans) | 10400304 | 112407 | 1.19 | 0.0180 | 0.1349 |
| Eaf2 | ELL associated factor 2 | 10439346 | 106389 | 1.18 | 0.0085 | 0.0576 |
| Elovl7 | ELOVL family member 7, elongation of long chain fatty acids (yeast) | 10407072 | 74559 | 1.39 | 0.0025 | 0.1435 |
| Emb | embigin | 10407327 | 13723 | 1.30 | 0.0089 | 0.1059 |
| Efs | embryonal Fyn-associated substrate | 10419892 | 13644 | 1.21 | 0.0049 | 0.1032 |
| Emcn | endomucin | 10496359 | 59308 | 1.26 | 0.0186 | 0.0209 |
| Edn1 | endothelin 1 | 10404783 | 13614 | 1.20 | 0.0004 | 0.0159 |
| Elmo3 | engulfment and cell motility 3, ced-12 homolog (C. elegans) | 10574694 | 234683 | 1.26 | 0.0294 | 0.0165 |
| Eno3 | enolase 3, beta muscle | 10377938 | 13808 | 1.19 | 0.0072 | 0.0340 |
| Eno4 | enolase 4 | 10464342 | 226265 | 1.40 | 0.0152 | 0.0454 |
| Ephb1 | Eph receptor B1 | 10596115 | 270190 | 1.21 | 0.0099 | 0.0418 |
| Efemp2 | epidermal growth factor-containing fibulin-like extracellular matrix protein 2 | 10460603 | 58859 | 1.25 | 0.0153 | 0.0413 |
| Ephx1 | epoxide hydrolase 1, microsomal | 10360684 | 13849 | 1.31 | 0.0085 | 0.0823 |
| Eps8l2 | EPS8-like 2 | 10558880 | 98845 | 1.34 | 0.0160 | 0.0211 |
| Epn3 | epsin 3 | 10389990 | 71889 | 1.34 | 0.0022 | 0.1214 |
| Epb4.1 | erythrocyte protein band 4.1 | 10516823 | 269587 | 1.16 | 0.0069 | 0.0978 |
| Eif2s3y | eukaryotic translation initiation factor 2, subunit 3, structural gene Y-linked | 10608001 | 26908 | 1.19 | 0.0032 | 0.1139 |
| Eif4e | eukaryotic translation initiation factor 4E | 10496490 | 13684 | 1.16 | 0.0087 | 0.0702 |
| Exd1 | exonuclease 3'-5' domain containing 1 | 10486241 | 241624 | 1.19 | 0.0093 | 0.0575 |
| Eapa2 | experimental autoimmune prostatitis antigen 2 | 10544444 | 403088 | 1.20 | 0.0003 | 0.2298 |
| Ezr | ezrin | 10447602 | 22350 | 1.19 | 0.0060 | 0.2076 |
| Fbxo36 | F-box protein 36 | 10347910 | 66153 | 1.20 | 0.0084 | 0.1672 |
| Fbxo46 | F-box protein 46 | 10550597 | 243867 | 1.17 | 0.0100 | 0.0696 |
| F11r | F11 receptor | 10351623 | 16456 | 1.36 | 0.0156 | 0.0585 |
| Fcgr4 | Fc receptor, IgG, low affinity IV | 10351509 | 246256 | 1.16 | 0.0054 | 0.1406 |
| Fes | feline sarcoma oncogene | 10564938 | 14159 | 1.17 | 0.0081 | 0.0738 |
| Frrs1 | ferric-chelate reductase 1 | 10495596 | 20321 | 1.32 | 0.0182 | 0.0337 |
| Fhdc1 | FH2 domain containing 1 | 10499062 | 229474 | 1.16 | 0.0072 | 0.1296 |
| Fbn1 | fibrillin 1 | 10487040 | 14118 | 1.19 | 0.0052 | 0.2161 |
| Fap | fibroblast activation protein | 10483081 | 14089 | 1.89 | 0.0132 | 0.0090 |
| Fgfbp1 | fibroblast growth factor binding protein 1 | 10529819 | 14181 | 1.33 | 0.0154 | 0.0290 |
| Fgfr2 | fibroblast growth factor receptor 2 | 10568436 | 14183 | 1.16 | 0.0085 | 0.1885 |
| Fbln5 | fibulin 5 | 10402211 | 23876 | 1.19 | 0.0133 | 0.1027 |
| Fbln7 | fibulin 7 | 10475932 | 70370 | 1.17 | 0.0117 | 0.1335 |
| Filip1l | filamin A interacting protein 1-like | 10436369 | 78749 | 1.16 | 0.0142 | 0.0624 |
| Flna | filamin, alpha | 10605256 | 192176 | 1.23 | 0.0147 | 0.0364 |
| Fkbp9 | FK506 binding protein 9 | 10538547 | 27055 | 1.20 | 0.0121 | 0.1282 |
| Fmo1 | flavin containing monooxygenase 1 | 10359571 | 14261 | 1.23 | 0.0021 | 0.1007 |
| Folr1 | folate receptor 1 (adult) | 10566034 | 14275 | 1.85 | 0.0002 | 0.0756 |
| Foxc1 | forkhead box C1 | 10404407 | 17300 | 1.16 | 0.0035 | 0.1991 |
| Foxc2 | forkhead box C2 | 10576051 | 14234 | 1.23 | 0.0086 | 0.1322 |
| Foxd1 | forkhead box D1 | 10406845 | 15229 | 1.31 | 0.0022 | 0.0529 |
| Foxf2 | forkhead box F2 | 10404404 | 14238 | 1.27 | 0.0049 | 0.0158 |
| Foxj1 | forkhead box J1 | 10393266 | 15223 | 1.24 | 0.0071 | 0.1124 |
| Fhad1 | forkhead-associated (FHA) phosphopeptide binding domain 1 | 10518075 | 329977 | 1.44 | 0.0184 | 0.0261 |
| Frem1 | Fras1 related extracellular matrix protein 1 | 10514088 | 329872 | 1.54 | 0.0087 | 0.0552 |
| Fxn | frataxin | 10466771 | 14297 | 1.18 | 0.0008 | 0.0993 |
| Fzd2 | frizzled homolog 2 (Drosophila) | 10381603 | 57265 | 1.18 | 0.0146 | 0.0743 |
| Fzd4 | frizzled homolog 4 (Drosophila) | 10554808 | 14366 | 1.32 | 0.0114 | 0.0781 |
| Fzd6 | frizzled homolog 6 (Drosophila) | 10423825 | 14368 | 1.21 | 0.0271 | 0.0436 |
| Fut11 | fucosyltransferase 11 | 10413012 | 73068 | 1.19 | 0.0091 | 0.1059 |
| Fxyd1 | FXYD domain-containing ion transport regulator 1 | 10562211 | 56188 | 1.27 | 0.0088 | 0.1098 |
| Gpatch8 | G patch domain containing 8 | 10391746 | 237943 | 1.18 | 0.0037 | 0.0644 |
| Gpr108 | G protein-coupled receptor 108 | 10452356 | 78308 | 1.18 | 0.0055 | 0.1561 |
| Gpr125 | G protein-coupled receptor 125 | 10529957 | 70693 | 1.15 | 0.0063 | 0.2110 |
| Gpr126 | G protein-coupled receptor 126 | 10367982 | 215798 | 1.26 | 0.0127 | 0.0168 |
| Gpr153 | G protein-coupled receptor 153 | 10510700 | 100129 | 1.18 | 0.0066 | 0.2710 |
| Grk1 | G protein-coupled receptor kinase 1 | 10570379 | 24013 | 1.18 | 0.0017 | 0.1533 |
| Grk4 | G protein-coupled receptor kinase 4 | 10521243 | 14772 | 1.17 | 0.0006 | 0.1805 |
| Gprc5c | G protein-coupled receptor, family C, group 5, member C | 10382425 | 70355 | 1.18 | 0.0173 | 0.0435 |
| Galm | galactose mutarotase | 10447084 | 319625 | 1.15 | 0.0047 | 0.2495 |
| Glb1l | galactosidase, beta 1-like | 10355785 | 74577 | 1.24 | 0.0112 | 0.0821 |
| Ggnbp1 | gametogenetin binding protein 1 | 10443120 | 70772 | 1.23 | 0.0234 | 0.0139 |
| Ggt5 | gamma-glutamyltransferase 5 | 10364072 | 23887 | 1.24 | 0.0222 | 0.0081 |
| Gje1 | gap junction protein, epsilon 1 | 10368021 | 76743 | 1.20 | 0.0021 | 0.1017 |
| Gjc1 | gap junction protein, gamma 1 | 10391762 | 14615 | 1.22 | 0.0075 | 0.0311 |
| Gast | gastrin | 10381118 | 14459 | 1.18 | 0.0082 | 0.0562 |
| Gkn3 | gastrokine 3 | 10546001 | 68888 | 1.63 | 0.0188 | 0.0004 |
| Gata2 | GATA binding protein 2 | 10539873 | 14461 | 1.20 | 0.0242 | 0.0160 |
| Gata5 | GATA binding protein 5 | 10479369 | 14464 | 1.15 | 0.0064 | 0.1768 |
| Gsn | gelsolin | 10471655 | 227753 | 1.20 | 0.0048 | 0.1205 |
| Ghdc | GH3 domain containing | 10391277 | 80860 | 1.15 | 0.0086 | 0.1204 |
| Gmfg | glia maturation factor, gamma | 10542981 | 63986 | 1.17 | 0.0265 | 0.0150 |
| Glis1 | GLIS family zinc finger 1 | 10506701 | 230587 | 1.18 | 0.0026 | 0.0551 |
| Gbgt1 | globoside alpha-1,3-N-acetylgalactosaminyltransferase 1 | 10470555 | 227671 | 1.18 | 0.0122 | 0.0805 |
| Grm8 | glutamate receptor, metabotropic 8 | 10543494 | 14823 | 1.20 | 0.0130 | 0.1470 |
| Gpx3 | glutathione peroxidase 3 | 10376201 | 14778 | 1.37 | 0.0038 | 0.0552 |
| Gpx8 | glutathione peroxidase 8 (putative) | 10412207 | 69590 | 1.45 | 0.0038 | 0.0985 |
| Gstt2 | glutathione S-transferase, theta 2 | 10370013 | 14872 | 1.23 | 0.0115 | 0.0413 |
| Gk5 | glycerol kinase 5 (putative) | 10587988 | 235533 | 1.16 | 0.0001 | 0.1815 |
| Gnmt | glycine N-methyltransferase | 10451451 | 14711 | 1.26 | 0.0050 | 0.0379 |
| Glt28d2 | glycosyltransferase 28 domain containing 2 | 10499108 | 320302 | 1.41 | 0.0135 | 0.0068 |
| Gyltl1b | glycosyltransferase-like 1B | 10485131 | 228366 | 1.40 | 0.0028 | 0.0554 |
| Gpihbp1 | GPI-anchored HDL-binding protein 1 | 10424695 | 68453 | 1.21 | 0.0004 | 0.1506 |
| Grn | granulin | 10381601 | 14824 | 1.20 | 0.0249 | 0.0294 |
| Gzmm | granzyme M (lymphocyte met-ase 1) | 10364460 | 16904 | 1.21 | 0.0069 | 0.0242 |
| Gas6 | growth arrest specific 6 | 10577164 | 14456 | 1.20 | 0.0055 | 0.1435 |
| Gfi1b | growth factor independent 1B | 10481304 | 14582 | 1.20 | 0.0155 | 0.0285 |
| Gnb3 | guanine nucleotide binding protein (G protein), beta 3 | 10547858 | 14695 | 2.25 | 0.0031 | 0.0831 |
| Gnat2 | guanine nucleotide binding protein, alpha transducing 2 | 10495259 | 14686 | 1.26 | 0.0033 | 0.0512 |
| Gbp2 | guanylate binding protein 2 | 10496592 | 14469 | 1.24 | 0.0151 | 0.0415 |
| Gbp3 | guanylate binding protein 3 | 10496580 | 55932 | 1.18 | 0.0053 | 0.0857 |
| Gbp4 | guanylate binding protein 4 | 10531987 | 17472 | 1.18 | 0.0063 | 0.0900 |
| Hes6 | hairy and enhancer of split 6 (Drosophila) | 10356593 | 55927 | 1.17 | 0.0006 | 0.1304 |
| Hey2 | hairy/enhancer-of-split related with YRPW motif 2 | 10368556 | 15214 | 1.20 | 0.0061 | 0.1199 |
| Hdhd2 | haloacid dehalogenase-like hydrolase domain containing 2 | 10456812 | 76987 | 1.20 | 0.0070 | 0.0601 |
| Hspb6 | heat shock protein, alpha-crystallin-related, B6 | 10551966 | 243912 | 1.30 | 0.0188 | 0.0529 |
| Hhatl | hedgehog acyltransferase-like | 10597841 | 74770 | 1.20 | 0.0105 | 0.1347 |
| Heg1 | HEG homolog 1 (zebrafish) | 10435266 | 77446 | 1.29 | 0.0098 | 0.0596 |
| Hba-a1 | hemoglobin alpha, adult chain 1 | 10375051 | 15122 | 2.54 | 0.0041 | 0.0315 |
| Hba-a2 | hemoglobin alpha, adult chain 2 | 10375058 | 110257 | 2.54 | 0.0043 | 0.0313 |
| Hbb-b1 | hemoglobin, beta adult major chain | 10566254 | 15129 | 2.60 | 0.0056 | 0.0192 |
| Hs3st3a1 | heparan sulfate (glucosamine) 3-O-sulfotransferase 3A1 | 10376956 | 15478 | 1.21 | 0.0046 | 0.0614 |
| Hpse | heparanase | 10531737 | 15442 | 1.38 | 0.0164 | 0.0481 |
| Hmgb1-rs17 | high mobility group box 1, related sequence 17 | 10571250 | 628431 | 1.15 | 0.0025 | 0.1068 |
| H2-Q7 | histocompatibility 2, Q region locus 7 | 10444830 | 15018 | 1.23 | 0.0042 | 0.0267 |
| Hist1h1b | histone cluster 1, H1b | 10408081 | 56702 | 1.17 | 0.0049 | 0.1025 |
| Hoxd13 | homeobox D13 | 10472946 | 15433 | 1.19 | 0.0044 | 0.0380 |
| Hoxd4 | homeobox D4 | 10472970 | 15436 | 1.35 | 0.0199 | 0.0258 |
| Msx1 | homeobox, msh-like 1 | 10529651 | 17701 | 1.37 | 0.0131 | 0.0132 |
| Hus1b | Hus1 homolog b (S. pombe) | 10408519 | 210554 | 1.31 | 0.0137 | 0.0024 |
| Hyal1 | hyaluronoglucosaminidase 1 | 10588691 | 15586 | 1.16 | 0.0027 | 0.2173 |
| Hyal3 | hyaluronoglucosaminidase 3 | 10588701 | 109685 | 1.19 | 0.0035 | 0.1373 |
| Hydin | hydrocephalus inducing | 10575380 | 244653 | 1.33 | 0.0010 | 0.1417 |
| Hsd3b7 | hydroxy-delta-5-steroid dehydrogenase, 3 beta- and steroid delta-isomerase 7 | 10557782 | 101502 | 1.16 | 0.0157 | 0.0556 |
| Hsd17b1 | hydroxysteroid (17-beta) dehydrogenase 1 | 10381218 | 15485 | 1.16 | 0.0109 | 0.0767 |
| Ighv1-72 | immunoglobulin heavy variable V1-72 | 10403043 | 619916 | 1.17 | 0.0043 | 0.1167 |
| Igl-V1 | immunoglobulin lambda chain, variable 1 | 10438405 | 16142 | 1.23 | 0.0148 | 0.0587 |
| Islr | immunoglobulin superfamily containing leucine-rich repeat | 10594044 | 26968 | 1.25 | 0.0080 | 0.1259 |
| Igsf5 | immunoglobulin superfamily, member 5 | 10437195 | 72058 | 1.28 | 0.0068 | 0.0110 |
| Igsf9 | immunoglobulin superfamily, member 9 | 10351801 | 93842 | 1.18 | 0.0096 | 0.0818 |
| Inadl | InaD-like (Drosophila) | 10506058 | 12695 | 1.20 | 0.0202 | 0.0668 |
| Inmt | indolethylamine N-methyltransferase | 10544932 | 21743 | 1.64 | 0.0181 | 0.0053 |
| Id1 | inhibitor of DNA binding 1 | 10477169 | 15901 | 1.18 | 0.0129 | 0.0566 |
| Id3 | inhibitor of DNA binding 3 | 10509163 | 15903 | 1.19 | 0.0150 | 0.1142 |
| Impa2 | inositol (myo)-1(or 4)-monophosphatase 2 | 10456383 | 114663 | 1.17 | 0.0069 | 0.1042 |
| Itpr3 | inositol 1,4,5-triphosphate receptor 3 | 10443131 | 16440 | 1.24 | 0.0084 | 0.0020 |
| Itpripl1 | inositol 1,4,5-triphosphate receptor interacting protein-like 1 | 10487359 | 73338 | 1.20 | 0.0083 | 0.0542 |
| Itpkb | inositol 1,4,5-trisphosphate 3-kinase B | 10352234 | 320404 | 1.17 | 0.0021 | 0.1505 |
| Inppl1 | inositol polyphosphate phosphatase-like 1 | 10565996 | 16332 | 1.16 | 0.0027 | 0.0978 |
| Insl5 | insulin-like 5 | 10514708 | 23919 | 1.18 | 0.0024 | 0.1209 |
| Igf2 | insulin-like growth factor 2 | 10569344 | 16002 | 1.51 | 0.0008 | 0.0516 |
| Igfbp1 | insulin-like growth factor binding protein 1 | 10374223 | 16006 | 1.23 | 0.0034 | 0.0487 |
| Igfbp2 | insulin-like growth factor binding protein 2 | 10347277 | 16008 | 1.66 | 0.0020 | 0.0534 |
| Itga1 | integrin alpha 1 | 10412298 | 109700 | 1.19 | 0.0038 | 0.0319 |
| Itga5 | integrin alpha 5 (fibronectin receptor alpha) | 10433114 | 16402 | 1.24 | 0.0182 | 0.0028 |
| Itfg3 | integrin alpha FG-GAP repeat containing 3 | 10449266 | 106581 | 1.20 | 0.0135 | 0.1154 |
| Itih5 | inter-alpha (globulin) inhibitor H5 | 10469151 | 209378 | 1.16 | 0.0129 | 0.0553 |
| Ifna12 | interferon alpha 12 | 10514300 | 242519 | 1.25 | 0.0079 | 0.1029 |
| Ifi30 | interferon gamma inducible protein 30 | 10579347 | 65972 | 1.25 | 0.0254 | 0.0392 |
| Ifitm2 | interferon induced transmembrane protein 2 | 10553299 | 80876 | 1.16 | 0.0020 | 0.1444 |
| Ifitm3 | interferon induced transmembrane protein 3 | 10569017 | 66141 | 1.24 | 0.0066 | 0.1108 |
| Ifih1 | interferon induced with helicase C domain 1 | 10483110 | 71586 | 1.17 | 0.0104 | 0.0769 |
| Irf6 | interferon regulatory factor 6 | 10352815 | 54139 | 1.17 | 0.0100 | 0.0716 |
| Ifi35 | interferon-induced protein 35 | 10381408 | 70110 | 1.22 | 0.0261 | 0.0041 |
| Ifit2 | interferon-induced protein with tetratricopeptide repeats 2 | 10462613 | 15958 | 1.15 | 0.0116 | 0.1789 |
| Ifi27l1 | interferon, alpha-inducible protein 27 like 1 | 10397975 | 52668 | 1.20 | 0.0057 | 0.1474 |
| Il10rb | interleukin 10 receptor, beta | 10436841 | 16155 | 1.23 | 0.0049 | 0.0978 |
| Il12a | interleukin 12a | 10492540 | 16159 | 1.23 | 0.0173 | 0.0051 |
| Il15 | interleukin 15 | 10579958 | 16168 | 1.17 | 0.0067 | 0.1169 |
| Il15ra | interleukin 15 receptor, alpha chain | 10469289 | 16169 | 1.15 | 0.0051 | 0.1281 |
| Il17rd | interleukin 17 receptor D | 10413398 | 171463 | 1.24 | 0.0161 | 0.0169 |
| Ifltd1 | intermediate filament tail domain containing 1 | 10549265 | 74071 | 1.20 | 0.0041 | 0.0175 |
| Intu | inturned planar cell polarity effector homolog (Drosophila) | 10491753 | 380614 | 1.17 | 0.0085 | 0.1143 |
| Iqcd | IQ motif containing D | 10525103 | 75732 | 1.34 | 0.0271 | 0.0071 |
| Iqgap1 | IQ motif containing GTPase activating protein 1 | 10565018 | 29875 | 1.18 | 0.0002 | 0.1584 |
| Iqca | IQ motif containing with AAA domain | 10356498 | 74918 | 1.27 | 0.0201 | 0.0399 |
| Klk7 | kallikrein related-peptidase 7 (chymotryptic, stratum corneum) | 10552508 | 23993 | 1.28 | 0.0055 | 0.0304 |
| Kdelc2 | KDEL (Lys-Asp-Glu-Leu) containing 2 | 10585338 | 68304 | 1.17 | 0.0079 | 0.1038 |
| Klhdc7a | kelch domain containing 7A | 10517727 | 242721 | 1.16 | 0.0032 | 0.2984 |
| Klhl36 | kelch-like 36 (Drosophila) | 10575955 | 234796 | 1.16 | 0.0045 | 0.1896 |
| Krt18 | keratin 18 | 10427075 | 16668 | 1.45 | 0.0002 | 0.1208 |
| Krtcap3 | keratinocyte associated protein 3 | 10520753 | 69815 | 1.16 | 0.0004 | 0.1269 |
| Kirrel | kin of IRRE like (Drosophila) | 10499168 | 170643 | 1.15 | 0.0124 | 0.0819 |
| Kdr | kinase insert domain protein receptor | 10530692 | 16542 | 1.26 | 0.0214 | 0.0082 |
| Kif9 | kinesin family member 9 | 10589541 | 16578 | 1.26 | 0.0083 | 0.0771 |
| Kifc1 | kinesin family member C1 | 10443047 | 16580 | 1.19 | 0.0146 | 0.0403 |
| Kl | klotho | 10527870 | 16591 | 1.63 | 0.0008 | 0.1061 |
| Kremen1 | kringle containing transmembrane protein 1 | 10383970 | 84035 | 1.18 | 0.0052 | 0.1129 |
| Lama5 | laminin, alpha 5 | 10490384 | 16776 | 1.40 | 0.0222 | 0.0138 |
| Lamb2 | laminin, beta 2 | 10588942 | 16779 | 1.16 | 0.0062 | 0.0680 |
| Lce1c | late cornified envelope 1C | 10493889 | 73719 | 1.19 | 0.0007 | 0.3033 |
| Lce3c | late cornified envelope 3C | 10493900 | 94060 | 1.26 | 0.0035 | 0.0472 |
| Ltbp3 | latent transforming growth factor beta binding protein 3 | 10460666 | 16998 | 1.22 | 0.0220 | 0.0368 |
| Lgals3bp | lectin, galactoside-binding, soluble, 3 binding protein | 10393573 | 19039 | 1.45 | 0.0045 | 0.0573 |
| Lepr | leptin receptor | 10506301 | 16847 | 1.45 | 0.0007 | 0.0916 |
| Lrrc23 | leucine rich repeat containing 23 | 10547820 | 16977 | 1.45 | 0.0174 | 0.0236 |
| Lrrc43 | leucine rich repeat containing 43 | 10525575 | 381741 | 1.17 | 0.0112 | 0.0533 |
| Lrrc46 | leucine rich repeat containing 46 | 10390319 | 69297 | 1.21 | 0.0103 | 0.0603 |
| Lrrc56 | leucine rich repeat containing 56 | 10558825 | 70552 | 1.16 | 0.0159 | 0.0588 |
| Lrig3 | leucine-rich repeats and immunoglobulin-like domains 3 | 10366746 | 320398 | 1.21 | 0.0005 | 0.1158 |
| Lrriq1 | leucine-rich repeats and IQ motif containing 1 | 10372151 | 74978 | 1.30 | 0.0021 | 0.1704 |
| Lekr1 | leucine, glutamate and lysine rich 1 | 10492442 | 624866 | 1.20 | 0.0176 | 0.0414 |
| Ltc4s | leukotriene C4 synthase | 10385583 | 17001 | 1.43 | 0.0044 | 0.0947 |
| Lpxn | leupaxin | 10461765 | 107321 | 1.18 | 0.0136 | 0.0853 |
| Lhx3 | LIM homeobox protein 3 | 10480912 | 16871 | 1.20 | 0.0090 | 0.0662 |
| Lsr | lipolysis stimulated lipoprotein receptor | 10562181 | 54135 | 1.23 | 0.0012 | 0.0264 |
| Lbp | lipopolysaccharide binding protein | 10478048 | 16803 | 1.72 | 0.0034 | 0.0802 |
| Pygl | liver glycogen phosphorylase | 10400844 | 110095 | 1.19 | 0.0058 | 0.1198 |
| Ldlrap1 | low density lipoprotein receptor adaptor protein 1 | 10517301 | 100017 | 1.17 | 0.0148 | 0.0009 |
| Lrp5 | low density lipoprotein receptor-related protein 5 | 10464504 | 16973 | 1.18 | 0.0147 | 0.0968 |
| Lrp10 | low-density lipoprotein receptor-related protein 10 | 10415065 | 65107 | 1.19 | 0.0062 | 0.1426 |
| Lhb | luteinizing hormone beta | 10552942 | 16866 | 1.23 | 0.0118 | 0.0321 |
| Lyl1 | lymphoblastomic leukemia 1 | 10573419 | 17095 | 1.16 | 0.0056 | 0.0985 |
| Lamp2 | lysosomal-associated membrane protein 2 | 10604187 | 16784 | 1.15 | 0.0062 | 0.1658 |
| Lyz1 | lysozyme 1 | 10372652 | 17110 | 1.36 | 0.0144 | 0.0389 |
| Loxl1 | lysyl oxidase-like 1 | 10594066 | 16949 | 1.18 | 0.0224 | 0.0753 |
| Loxl3 | lysyl oxidase-like 3 | 10539263 | 16950 | 1.20 | 0.0070 | 0.1100 |
| Mpa2l | macrophage activation 2 like | 10531994 | 100702 | 1.20 | 0.0096 | 0.0256 |
| Mad2l2 | MAD2 mitotic arrest deficient-like 2 (yeast) | 10510286 | 71890 | 1.18 | 0.0061 | 0.1100 |
| Mfsd7c | major facilitator superfamily domain containing 7C | 10397364 | 217721 | 1.35 | 0.0105 | 0.0407 |
| Mr1 | major histocompatibility complex, class I-related | 10358982 | 15064 | 1.24 | 0.0044 | 0.1564 |
| Mvp | major vault protein | 10568115 | 78388 | 1.21 | 0.0159 | 0.0629 |
| Mamdc2 | MAM domain containing 2 | 10466712 | 71738 | 1.31 | 0.0146 | 0.0460 |
| Mrc2 | mannose receptor, C type 2 | 10381898 | 17534 | 1.21 | 0.0240 | 0.0071 |
| Man2b1 | mannosidase 2, alpha B1 | 10573583 | 17159 | 1.17 | 0.0037 | 0.1690 |
| Mrgpra3 | MAS-related GPR, member A3 | 10563722 | 233222 | 1.23 | 0.0020 | 0.1071 |
| Mgp | matrix Gla protein | 10548879 | 17313 | 1.39 | 0.0076 | 0.0279 |
| Mmp15 | matrix metallopeptidase 15 | 10574350 | 17388 | 1.25 | 0.0035 | 0.1461 |
| Mmp23 | matrix metallopeptidase 23 | 10519140 | 26561 | 1.16 | 0.0004 | 0.1803 |
| Mxra8 | matrix-remodelling associated 8 | 10511180 | 74761 | 1.24 | 0.0012 | 0.1817 |
| Mks1 | Meckel syndrome, type 1 | 10380189 | 380718 | 1.23 | 0.0195 | 0.0311 |
| Med4 | mediator of RNA polymerase II transcription, subunit 4 homolog (yeast) | 10603706 | 67381 | 1.26 | 0.0030 | 0.1059 |
| Mrap2 | melanocortin 2 receptor accessory protein 2 | 10587633 | 244958 | 1.27 | 0.0103 | 0.0540 |
| Mpp7 | membrane protein, palmitoylated 7 (MAGUK p55 subfamily member 7) | 10457357 | 75739 | 1.28 | 0.0066 | 0.0406 |
| Ms4a15 | membrane-spanning 4-domains, subfamily A, member 15 | 10466120 | 545279 | 1.54 | 0.0001 | 0.0913 |
| Mesp1 | mesoderm posterior 1 | 10564813 | 17292 | 1.16 | 0.0011 | 0.1165 |
| Msgn1 | mesogenin 1 | 10399387 | 56184 | 1.26 | 0.0125 | 0.0325 |
| Mars2 | methionine-tRNA synthetase 2 (mitochondrial) | 10346328 | 212679 | 1.15 | 0.0105 | 0.1266 |
| Mccc1 | methylcrotonoyl-Coenzyme A carboxylase 1 (alpha) | 10497773 | 72039 | 1.18 | 0.0049 | 0.1699 |
| Mettl7a1 | methyltransferase like 7A1 | 10426891 | 70152 | 1.16 | 0.0008 | 0.1879 |
| H2-gs10 | MHC class I like protein GS10 | 10444814 | 436493 | 1.25 | 0.0186 | 0.0801 |
| H2-t9 | MHC class Ib T9 | 10450699 | 630294 | 1.17 | 0.0004 | 0.1004 |
| Mitf | microphthalmia-associated transcription factor | 10540248 | 17342 | 1.19 | 0.0122 | 0.1278 |
| Mir100 | microRNA 100 | 10584589 | 723892 | 1.22 | 0.0114 | 0.0723 |
| Mir101a | microRNA 101a | 10514697 | 387143 | 1.18 | 0.0021 | 0.1584 |
| Mir15a | microRNA 15a | 10420668 | 387174 | 1.18 | 0.0070 | 0.0840 |
| Mir181b-1 | microRNA 181b-1 | 10350341 | 723890 | 1.26 | 0.0040 | 0.0320 |
| Mir30c-2 | microRNA 30c-2 | 10345089 | 723964 | 1.24 | 0.0140 | 0.0058 |
| Mir30e | microRNA 30e | 10515981 | 723836 | 1.23 | 0.0013 | 0.0910 |
| Mir384 | microRNA 384 | 10606257 | 723861 | 1.23 | 0.0052 | 0.0808 |
| Mir455 | microRNA 455 | 10505436 | 735262 | 1.23 | 0.0023 | 0.1222 |
| Mir504 | microRNA 504 | 10604761 | 100124476 | 1.24 | 0.0031 | 0.0750 |
| Mir505 | microRNA 505 | 10604832 | 751545 | 1.22 | 0.0127 | 0.0358 |
| Mir540 | microRNA 540 | 10398336 | 723880 | 1.16 | 0.0008 | 0.1317 |
| Mir669c | microRNA 669c | 10469239 | 735273 | 1.20 | 0.0061 | 0.1525 |
| Mir9-3 | microRNA 9-3 | 10554323 | 723968 | 1.21 | 0.0090 | 0.0214 |
| Mtfr1 | mitochondrial fission regulator 1 | 10490955 | 67472 | 1.27 | 0.0066 | 0.0976 |
| Map3k15 | mitogen-activated protein kinase kinase kinase 15 | 10602865 | 270672 | 1.30 | 0.0061 | 0.0594 |
| Morn2 | MORN repeat containing 2 | 10447100 | 378462 | 1.15 | 0.0045 | 0.1932 |
| Morn3 | MORN repeat containing 3 | 10533594 | 74890 | 1.17 | 0.0199 | 0.0127 |
| Mospd1 | motile sperm domain containing 1 | 10604630 | 70380 | 1.18 | 0.0050 | 0.2380 |
| Muc1 | mucin 1, transmembrane | 10493474 | 17829 | 1.24 | 0.0071 | 0.0188 |
| Pygm | muscle glycogen phosphorylase | 10460947 | 19309 | 1.16 | 0.0014 | 0.2607 |
| Mycbpap | MYCBP associated protein | 10390001 | 104601 | 1.16 | 0.0101 | 0.0535 |
| Mpz | myelin protein zero | 10351525 | 17528 | 1.18 | 0.0080 | 0.0798 |
| Mpzl2 | myelin protein zero-like 2 | 10584827 | 14012 | 1.24 | 0.0142 | 0.1015 |
| Mybl2 | myeloblastosis oncogene-like 2 | 10478355 | 17865 | 1.19 | 0.0089 | 0.1204 |
| Myoc | myocilin | 10351131 | 17926 | 1.20 | 0.0051 | 0.2050 |
| Myof | myoferlin | 10467258 | 226101 | 1.22 | 0.0145 | 0.0489 |
| Myo5c | myosin VC | 10587150 | 208943 | 1.25 | 0.0114 | 0.0592 |
| Myo7a | myosin VIIA | 10565634 | 17921 | 1.26 | 0.0140 | 0.0675 |
| Myo18b | myosin XVIIIb | 10532578 | 74376 | 1.20 | 0.0076 | 0.0606 |
| Myh14 | myosin, heavy polypeptide 14 | 10562856 | 71960 | 1.19 | 0.0101 | 0.1081 |
| Myh7 | myosin, heavy polypeptide 7, cardiac muscle, beta | 10419934 | 140781 | 1.19 | 0.0152 | 0.0754 |
| Mylk | myosin, light polypeptide kinase | 10435345 | 107589 | 1.30 | 0.0143 | 0.0422 |
| Naaladl2 | N-acetylated alpha-linked acidic dipeptidase-like 2 | 10497490 | 635702 | 1.24 | 0.0228 | 0.0351 |
| Nqo1 | NAD(P)H dehydrogenase, quinone 1 | 10581538 | 18104 | 1.26 | 0.0141 | 0.1368 |
| Npr1 | natriuretic peptide receptor 1 | 10499811 | 18160 | 1.25 | 0.0075 | 0.0764 |
| Npr3 | natriuretic peptide receptor 3 | 10427796 | 18162 | 1.22 | 0.0098 | 0.0837 |
| Ntn4 | netrin 4 | 10365817 | 57764 | 1.16 | 0.0004 | 0.1147 |
| Neu2 | neuraminidase 2 | 10348234 | 23956 | 1.22 | 0.0081 | 0.1994 |
| Nradd | neurotrophin receptor associated death domain | 10597173 | 67169 | 1.22 | 0.0275 | 0.0088 |
| Nid2 | nidogen 2 | 10412921 | 18074 | 1.33 | 0.0125 | 0.0492 |
| Nek11 | NIMA (never in mitosis gene a)-related expressed kinase 11 | 10596327 | 208583 | 1.34 | 0.0216 | 0.0258 |
| Nek5 | NIMA (never in mitosis gene a)-related expressed kinase 5 | 10577471 | 330721 | 1.35 | 0.0131 | 0.0754 |
| Nek8 | NIMA (never in mitosis gene a)-related expressed kinase 8 | 10388758 | 140859 | 1.18 | 0.0094 | 0.1072 |
| Naip2 | NLR family, apoptosis inhibitory protein 2 | 10411595 | 17948 | 1.17 | 0.0003 | 0.1691 |
| Nme5 | non-metastatic cells 5, protein expressed in (nucleoside-diphosphate kinase) | 10458122 | 75533 | 1.16 | 0.0002 | 0.2034 |
| Notch2 | Notch gene homolog 2 (Drosophila) | 10494595 | 18129 | 1.27 | 0.0125 | 0.0578 |
| Notch4 | Notch gene homolog 4 (Drosophila) | 10444352 | 18132 | 1.16 | 0.0003 | 0.1085 |
| Sp100 | nuclear antigen Sp100 | 10347948 | 20684 | 1.19 | 0.0067 | 0.0032 |
| Nfyc | nuclear transcription factor-Y gamma | 10515974 | 18046 | 1.20 | 0.0003 | 0.1026 |
| Nup210l | nucleoporin 210-like | 10493662 | 77595 | 1.19 | 0.0111 | 0.0874 |
| Nxn | nucleoredoxin | 10388532 | 18230 | 1.24 | 0.0060 | 0.1178 |
| Nod1 | nucleotide-binding oligomerization domain containing 1 | 10544891 | 107607 | 1.48 | 0.0244 | 0.0151 |
| Nynrin | NYN domain and retroviral integrase containing | 10415408 | 277154 | 1.17 | 0.0027 | 0.1945 |
| Oca2 | oculocutaneous albinism II | 10553743 | 18431 | 1.68 | 0.0066 | 0.0525 |
| Olfr1366 | olfactory receptor 1366 | 10408056 | 258280 | 1.20 | 0.0058 | 0.0708 |
| Olfr1371 | olfactory receptor 1371 | 10385774 | 276865 | 1.19 | 0.0055 | 0.0673 |
| Olfr1427 | olfactory receptor 1427 | 10466286 | 258674 | 1.21 | 0.0020 | 0.1055 |
| Olfr1507 | olfactory receptor 1507 | 10419723 | 57269 | 1.57 | 0.0010 | 0.0534 |
| Olfr288 | olfactory receptor 288 | 10432129 | 545140 | 1.48 | 0.0139 | 0.0137 |
| Olfr319 | olfactory receptor 319 | 10376406 | 258493 | 1.30 | 0.0112 | 0.0194 |
| Olfr402 | olfactory receptor 402 | 10378429 | 258703 | 1.18 | 0.0020 | 0.0573 |
| Olfr412 | olfactory receptor 412 | 10378443 | 258153 | 1.21 | 0.0037 | 0.1767 |
| Olfr430 | olfactory receptor 430 | 10351888 | 258713 | 1.32 | 0.0158 | 0.0355 |
| Olfr486 | olfactory receptor 486 | 10566680 | 258489 | 1.30 | 0.0150 | 0.0582 |
| Olfr518 | olfactory receptor 518 | 10566705 | 258303 | 1.26 | 0.0048 | 0.0565 |
| Olfr574 | olfactory receptor 574 | 10555753 | 258357 | 1.61 | 0.0269 | 0.0015 |
| Olfr744 | olfactory receptor 744 | 10414487 | 257884 | 1.28 | 0.0035 | 0.1413 |
| Olfr816 | olfactory receptor 816 | 10373655 | 258667 | 1.35 | 0.0009 | 0.1168 |
| Olfr850 | olfactory receptor 850 | 10591194 | 258516 | 1.27 | 0.0055 | 0.0693 |
| Ooep | oocyte expressed protein homolog (dog) | 10595159 | 67968 | 1.20 | 0.0043 | 0.0307 |
| Orai1 | ORAI calcium release-activated calcium modulator 1 | 10525464 | 109305 | 1.22 | 0.0001 | 0.0923 |
| Ormdl2 | ORM1-like 2 (S. cerevisiae) | 10373577 | 66844 | 1.18 | 0.0068 | 0.1803 |
| Otx1 | orthodenticle homolog 1 (Drosophila) | 10384615 | 18423 | 1.16 | 0.0099 | 0.0857 |
| Otx2 | orthodenticle homolog 2 (Drosophila) | 10419356 | 18424 | 1.74 | 0.0006 | 0.0632 |
| Odf3b | outer dense fiber of sperm tails 3B | 10431558 | 70113 | 1.23 | 0.0160 | 0.0663 |
| Pax3 | paired box gene 3 | 10355916 | 18505 | 1.21 | 0.0038 | 0.0568 |
| Prrx2 | paired related homeobox 2 | 10471067 | 20204 | 1.21 | 0.0120 | 0.0239 |
| Pon3 | paraoxonase 3 | 10542993 | 269823 | 1.61 | 0.0003 | 0.0904 |
| Pdzd2 | PDZ domain containing 2 | 10427816 | 68070 | 1.23 | 0.0221 | 0.0600 |
| Pm20d1 | peptidase M20 domain containing 1 | 10349694 | 212933 | 1.19 | 0.0031 | 0.1463 |
| Pin1l | peptidylprolyl cis/trans isomerase, NIMA-interacting 1-like | 10485594 | 241593 | 1.21 | 0.0046 | 0.0551 |
| Phxr1 | per-hexamer repeat gene 1 | 10468970 | 18686 | 1.15 | 0.0040 | 0.1187 |
| Plin3 | perilipin 3 | 10452030 | 66905 | 1.15 | 0.0114 | 0.2049 |
| Hspg2 | perlecan (heparan sulfate proteoglycan 2) | 10509280 | 15530 | 1.26 | 0.0172 | 0.0009 |
| Perp | PERP, TP53 apoptosis effector | 10361887 | 64058 | 1.45 | 0.0042 | 0.0918 |
| Phactr4 | phosphatase and actin regulator 4 | 10516910 | 100169 | 1.21 | 0.0056 | 0.0781 |
| Ppap2c | phosphatidic acid phosphatase type 2C | 10370552 | 50784 | 1.21 | 0.0139 | 0.1032 |
| Pigz | phosphatidylinositol glycan anchor biosynthesis, class Z | 10434993 | 239827 | 1.15 | 0.0051 | 0.1835 |
| Pisd | phosphatidylserine decarboxylase | 10529239 | 320951 | 1.16 | 0.0070 | 0.1191 |
| Pisd-ps1 | phosphatidylserine decarboxylase, pseudogene 1 | 10373702 | 236604 | 1.30 | 0.0109 | 0.0351 |
| Pik3ap1 | phosphoinositide-3-kinase adaptor protein 1 | 10467578 | 83490 | 1.17 | 0.0208 | 0.0638 |
| Pla2g3 | phospholipase A2, group III | 10373756 | 237625 | 1.24 | 0.0286 | 0.0071 |
| Pla2g12a | phospholipase A2, group XIIA | 10496015 | 66350 | 1.16 | 0.0103 | 0.0720 |
| Pla2g16 | phospholipase A2, group XVI | 10461093 | 225845 | 1.18 | 0.0041 | 0.1933 |
| Plcb3 | phospholipase C, beta 3 | 10465521 | 18797 | 1.16 | 0.0003 | 0.1696 |
| Plcd1 | phospholipase C, delta 1 | 10597575 | 18799 | 1.16 | 0.0097 | 0.1445 |
| Pld1 | phospholipase D1 | 10491106 | 18805 | 1.23 | 0.0146 | 0.0773 |
| Plscr1 | phospholipid scramblase 1 | 10356082 | 22038 | 1.23 | 0.0033 | 0.1003 |
| Plscr2 | phospholipid scramblase 2 | 10587799 | 18828 | 1.31 | 0.0047 | 0.0895 |
| Pltp | phospholipid transfer protein | 10489569 | 18830 | 1.32 | 0.0178 | 0.0389 |
| Phkg2 | phosphorylase kinase, gamma 2 (testis) | 10568266 | 68961 | 1.17 | 0.0018 | 0.1801 |
| Pih1d2 | PIH1 domain containing 2 | 10585206 | 72614 | 1.24 | 0.0062 | 0.0718 |
| Pkp1 | plakophilin 1 | 10358124 | 18772 | 1.24 | 0.0120 | 0.0253 |
| Pgcp | plasma glutamate carboxypeptidase | 10423556 | 54381 | 1.28 | 0.0053 | 0.1158 |
| Pear1 | platelet endothelial aggregation receptor 1 | 10499216 | 73182 | 1.26 | 0.0095 | 0.0335 |
| Pdgfd | platelet-derived growth factor, D polypeptide | 10583021 | 71785 | 1.32 | 0.0075 | 0.1021 |
| Plek2 | pleckstrin 2 | 10401149 | 27260 | 1.19 | 0.0161 | 0.1001 |
| Plekhg3 | pleckstrin homology domain containing, family G (with RhoGef domain) member 3 | 10396671 | 263406 | 1.20 | 0.0010 | 0.1428 |
| Phldb2 | pleckstrin homology-like domain, family B, member 2 | 10439710 | 208177 | 1.31 | 0.0235 | 0.0092 |
| Plxnb2 | plexin B2 | 10431424 | 140570 | 1.23 | 0.0112 | 0.1135 |
| Podn | podocan | 10514939 | 242608 | 1.20 | 0.0203 | 0.0313 |
| Pdpn | podoplanin | 10518147 | 14726 | 1.23 | 0.0057 | 0.1535 |
| Pvrl2 | poliovirus receptor-related 2 | 10560644 | 19294 | 1.17 | 0.0091 | 0.0978 |
| Parp3 | poly (ADP-ribose) polymerase family, member 3 | 10596492 | 235587 | 1.24 | 0.0170 | 0.0115 |
| Parp9 | poly (ADP-ribose) polymerase family, member 9 | 10435457 | 80285 | 1.20 | 0.0125 | 0.0658 |
| Kctd11 | potassium channel tetramerisation domain containing 11 | 10387689 | 216858 | 1.21 | 0.0035 | 0.0397 |
| Kcnmb1 | potassium large conductance calcium-activated channel, subfamily M, beta member 1 | 10375137 | 16533 | 1.16 | 0.0032 | 0.2541 |
| Kcne2 | potassium voltage-gated channel, Isk-related subfamily, gene 2 | 10436947 | 246133 | 2.09 | 0.0036 | 0.0394 |
| Kcnh6 | potassium voltage-gated channel, subfamily H (eag-related), member 6 | 10381994 | 192775 | 1.21 | 0.0074 | 0.0838 |
| Prdm1 | PR domain containing 1, with ZNF domain | 10368970 | 12142 | 1.18 | 0.0014 | 0.1426 |
| Pbxip1 | pre-B-cell leukemia transcription factor interacting protein 1 | 10493537 | 229534 | 1.23 | 0.0124 | 0.0783 |
| Pcolce2 | procollagen C-endopeptidase enhancer 2 | 10587880 | 76477 | 1.46 | 0.0041 | 0.0828 |
| Pcolce | procollagen C-endopeptidase enhancer protein | 10534862 | 18542 | 1.67 | 0.0040 | 0.0474 |
| Paqr5 | progestin and adipoQ receptor family member V | 10594277 | 74090 | 1.19 | 0.0169 | 0.0624 |
| Pdcd11 | programmed cell death 11 | 10468292 | 18572 | 1.20 | 0.0058 | 0.0563 |
| Prl7c1 | prolactin family 7, subfamily c, member 1 | 10408444 | 67505 | 1.37 | 0.0188 | 0.0393 |
| Prrg4 | proline rich Gla (G-carboxyglutamic acid) 4 (transmembrane) | 10485624 | 228413 | 1.47 | 0.0019 | 0.1253 |
| Prb1 | proline-rich protein BstNI subfamily 1 | 10548639 | 381833 | 1.17 | 0.0189 | 0.0402 |
| Prpmp5 | proline-rich protein MP5 | 10548656 | 381832 | 1.27 | 0.0113 | 0.0367 |
| Prom1 | prominin 1 | 10529824 | 19126 | 1.24 | 0.0016 | 0.0753 |
| Ptgis | prostaglandin I2 (prostacyclin) synthase | 10489878 | 19223 | 1.22 | 0.0168 | 0.0306 |
| Psmb9 | proteasome (prosome, macropain) subunit, beta type 9 (large multifunctional peptidase 2) | 10450145 | 16912 | 1.22 | 0.0010 | 0.0946 |
| Pacsin3 | protein kinase C and casein kinase substrate in neurons 3 | 10473861 | 80708 | 1.17 | 0.0088 | 0.1057 |
| Prkcq | protein kinase C, theta | 10469255 | 18761 | 1.23 | 0.0038 | 0.1822 |
| Ppp1r1b | protein phosphatase 1, regulatory (inhibitor) subunit 1B | 10380862 | 19049 | 1.21 | 0.0095 | 0.1410 |
| Ppp1r3b | protein phosphatase 1, regulatory (inhibitor) subunit 3B | 10571321 | 244416 | 1.36 | 0.0019 | 0.0116 |
| Ppm1f | protein phosphatase 1F (PP2C domain containing) | 10433937 | 68606 | 1.20 | 0.0055 | 0.0737 |
| Ppef2 | protein phosphatase, EF hand calcium-binding domain 2 | 10531348 | 19023 | 1.24 | 0.0245 | 0.0169 |
| Ptpla | protein tyrosine phosphatase-like (proline instead of catalytic arginine), member a | 10480249 | 30963 | 1.19 | 0.0096 | 0.0738 |
| Ptprf | protein tyrosine phosphatase, receptor type, F | 10515613 | 19268 | 1.16 | 0.0058 | 0.1454 |
| Ppfibp2 | PTPRF interacting protein, binding protein 2 (liprin beta 2) | 10556082 | 19024 | 1.18 | 0.0102 | 0.1763 |
| Pnp | purine-nucleoside phosphorylase | 10414514 | 18950 | 1.17 | 0.0122 | 0.1078 |
| Pyroxd2 | pyridine nucleotide-disulphide oxidoreductase domain 2 | 10467784 | 74580 | 1.18 | 0.0110 | 0.0968 |
| Rilp | Rab interacting lysosomal protein | 10378627 | 280408 | 1.32 | 0.0207 | 0.0020 |
| Rab11fip1 | RAB11 family interacting protein 1 (class I) | 10577954 | 75767 | 1.29 | 0.0151 | 0.0448 |
| Rep15 | RAB15 effector protein | 10542822 | 66532 | 1.19 | 0.0106 | 0.0387 |
| Rab20 | RAB20, member RAS oncogene family | 10577025 | 19332 | 1.30 | 0.0165 | 0.0061 |
| Rabep2 | rabaptin, RAB GTPase binding effector protein 2 | 10557405 | 70314 | 1.15 | 0.0116 | 0.0559 |
| Rem1 | rad and gem related GTP binding protein 1 | 10477140 | 19700 | 1.25 | 0.0040 | 0.0186 |
| Rad51ap1 | RAD51 associated protein 1 | 10548086 | 19362 | 1.22 | 0.0144 | 0.0073 |
| Rsph10b2 | radial spoke head 10 homolog B (Chlamydomonas) | 10527285 | 75136 | 1.21 | 0.0157 | 0.0542 |
| Rsph9 | radial spoke head 9 homolog (Chlamydomonas) | 10451213 | 75564 | 1.17 | 0.0008 | 0.2384 |
| Rdx | radixin | 10585318 | 19684 | 1.19 | 0.0160 | 0.0367 |
| Rassf9 | Ras association (RalGDS/AF-6) domain family (N-terminal) member 9 | 10366153 | 237504 | 1.38 | 0.0060 | 0.1204 |
| Rhod | ras homolog gene family, member D | 10464754 | 11854 | 1.29 | 0.0126 | 0.0778 |
| Rest | RE1-silencing transcription factor | 10522712 | 19712 | 1.15 | 0.0071 | 0.1915 |
| Rec8 | REC8 homolog (yeast) | 10415332 | 56739 | 1.21 | 0.0147 | 0.0625 |
| Rgs3 | regulator of G-protein signaling 3 | 10607113 | 50780 | 1.20 | 0.0071 | 0.1230 |
| Rgs22 | regulator of G-protein signalling 22 | 10428124 | 626596 | 1.34 | 0.0036 | 0.0965 |
| Rtel1 | regulator of telomere elongation helicase 1 | 10479560 | 269400 | 1.16 | 0.0010 | 0.1200 |
| Rxfp2 | relaxin/insulin-like family peptide receptor 2 | 10527713 | 140498 | 1.34 | 0.0029 | 0.0620 |
| Retn | resistin | 10569870 | 57264 | 1.25 | 0.0240 | 0.0137 |
| Rrh | retinal pigment epithelium derived rhodopsin homolog | 10502135 | 20132 | 1.38 | 0.0168 | 0.0223 |
| Sag | retinal S-antigen | 10348301 | 20215 | 1.50 | 0.0106 | 0.0515 |
| Rbl1 | retinoblastoma-like 1 (p107) | 10489127 | 19650 | 1.17 | 0.0118 | 0.1002 |
| Rai14 | retinoic acid induced 14 | 10427744 | 75646 | 1.27 | 0.0145 | 0.0244 |
| Rbp1 | retinol binding protein 1, cellular | 10588037 | 19659 | 1.33 | 0.0055 | 0.1139 |
| Rbp3 | retinol binding protein 3, interstitial | 10414034 | 19661 | 2.28 | 0.0036 | 0.0910 |
| Rdh10 | retinol dehydrogenase 10 (all-trans) | 10344952 | 98711 | 1.17 | 0.0089 | 0.1467 |
| Rdh5 | retinol dehydrogenase 5 | 10373588 | 19682 | 1.86 | 0.0053 | 0.0386 |
| Arhgef16 | Rho guanine nucleotide exchange factor (GEF) 16 | 10519012 | 230972 | 1.21 | 0.0087 | 0.0782 |
| Arhgef5 | Rho guanine nucleotide exchange factor (GEF) 5 | 10537834 | 54324 | 1.29 | 0.0253 | 0.0053 |
| Rho | rhodopsin | 10540984 | 212541 | 1.22 | 0.0140 | 0.0328 |
| Rhpn1 | rhophilin, Rho GTPase binding protein 1 | 10424711 | 14787 | 1.18 | 0.0211 | 0.0259 |
| Rprl1 | ribonuclease P RNA-like 1 | 10545192 | 19783 | 1.28 | 0.0003 | 0.1243 |
| Rnaset2a | ribonuclease T2A | 10441539 | 100037283 | 1.18 | 0.0071 | 0.1380 |
| Rnase11 | ribonuclease, RNase A family, 11 (non-active) | 10419557 | 497113 | 1.18 | 0.0077 | 0.0923 |
| Rps3a | ribosomal protein S3A | 10407120 | 20091 | 1.19 | 0.0033 | 0.1206 |
| Rnf152 | ring finger protein 152 | 10357003 | 320311 | 1.40 | 0.0183 | 0.0442 |
| Rnf213 | ring finger protein 213 | 10383214 | 672511 | 1.24 | 0.0155 | 0.0242 |
| Rnf43 | ring finger protein 43 | 10380116 | 207742 | 1.19 | 0.0042 | 0.1384 |
| Rbm15b | RNA binding motif protein 15B | 10596568 | 109095 | 1.15 | 0.0032 | 0.2007 |
| Rbm47 | RNA binding motif protein 47 | 10530269 | 245945 | 1.78 | 0.0096 | 0.0349 |
| Rbpms | RNA binding protein gene with multiple splicing | 10578123 | 19663 | 1.18 | 0.0006 | 0.0297 |
| Runx1 | runt related transcription factor 1 | 10441003 | 12394 | 1.20 | 0.0166 | 0.0525 |
| S100a6 | S100 calcium binding protein A6 (calcyclin) | 10493820 | 20200 | 1.15 | 0.0076 | 0.1607 |
| Sall3 | sal-like 3 (Drosophila) | 10459999 | 20689 | 1.16 | 0.0056 | 0.1097 |
| Sec14l5 | SEC14-like 5 (S. cerevisiae) | 10433373 | 665119 | 1.26 | 0.0139 | 0.0514 |
| Scrn2 | secernin 2 | 10380721 | 217140 | 1.15 | 0.0122 | 0.0379 |
| Sfrp1 | secreted frizzled-related protein 1 | 10570957 | 20377 | 1.38 | 0.0005 | 0.1025 |
| Sfrp4 | secreted frizzled-related protein 4 | 10403834 | 20379 | 1.18 | 0.0088 | 0.1530 |
| Sfrp5 | secreted frizzled-related sequence protein 5 | 10467744 | 54612 | 1.57 | 0.0192 | 0.0006 |
| Sema3a | sema domain, immunoglobulin domain (Ig), short basic domain, secreted, (semaphorin) 3A | 10519717 | 20346 | 1.19 | 0.0011 | 0.4763 |
| Sema3b | sema domain, immunoglobulin domain (Ig), short basic domain, secreted, (semaphorin) 3B | 10596680 | 20347 | 1.38 | 0.0071 | 0.0330 |
| Sema3f | sema domain, immunoglobulin domain (Ig), short basic domain, secreted, (semaphorin) 3F | 10596747 | 20350 | 1.31 | 0.0184 | 0.0277 |
| Serpinb1b | serine (or cysteine) peptidase inhibitor, clade B, member 1b | 10404447 | 282663 | 1.26 | 0.0077 | 0.0827 |
| Serpinh1 | serine (or cysteine) peptidase inhibitor, clade H, member 1 | 10565794 | 12406 | 1.20 | 0.0117 | 0.1105 |
| Serhl | serine hydrolase-like | 10425781 | 68607 | 1.19 | 0.0040 | 0.1089 |
| Serinc2 | serine incorporator 2 | 10516765 | 230779 | 1.17 | 0.0072 | 0.2112 |
| Spint2 | serine protease inhibitor, Kunitz type 2 | 10561712 | 20733 | 1.47 | 0.0144 | 0.0380 |
| Stk36 | serine/threonine kinase 36 (fused homolog, Drosophila) | 10347427 | 269209 | 1.20 | 0.0137 | 0.0665 |
| Stk39 | serine/threonine kinase 39, STE20/SPS1 homolog (yeast) | 10483381 | 53416 | 1.15 | 0.0034 | 0.2212 |
| Sdpr | serum deprivation response | 10346164 | 20324 | 1.22 | 0.0057 | 0.1038 |
| Sgk3 | serum/glucocorticoid regulated kinase 3 | 10344750 | 170755 | 1.29 | 0.0226 | 0.0360 |
| Sft2d2 | SFT2 domain containing 2 | 10359713 | 108735 | 1.24 | 0.0151 | 0.1150 |
| Sh3tc2 | SH3 domain and tetratricopeptide repeats 2 | 10456140 | 225608 | 1.20 | 0.0073 | 0.0438 |
| Sh3rf2 | SH3 domain containing ring finger 2 | 10455299 | 269016 | 1.39 | 0.0024 | 0.1376 |
| Sh3d19 | SH3 domain protein D19 | 10492864 | 27059 | 1.28 | 0.0122 | 0.0778 |
| Shank2 | SH3/ankyrin domain gene 2 | 10559341 | 210274 | 1.58 | 0.0102 | 0.0382 |
| Shisa3 | shisa homolog 3 (Xenopus laevis) | 10522288 | 330096 | 1.47 | 0.0077 | 0.0758 |
| Shroom4 | shroom family member 4 | 10598240 | 208431 | 1.17 | 0.0041 | 0.1021 |
| Sgol1 | shugoshin-like 1 (S. pombe) | 10451805 | 72415 | 1.27 | 0.0038 | 0.0344 |
| Scube3 | signal peptide, CUB domain, EGF-like 3 | 10443276 | 268935 | 1.42 | 0.0145 | 0.0304 |
| Stat5a | signal transducer and activator of transcription 5A | 10381172 | 20850 | 1.15 | 0.0002 | 0.0993 |
| Steap2 | six transmembrane epithelial antigen of prostate 2 | 10528008 | 74051 | 1.64 | 0.0044 | 0.0770 |
| Steap1 | six transmembrane epithelial antigen of the prostate 1 | 10528015 | 70358 | 1.90 | 0.0046 | 0.0870 |
| Snhg1 | small nucleolar RNA host gene (non-protein coding) 1 | 10461156 | 83673 | 1.32 | 0.0137 | 0.0234 |
| Snord32a | small nucleolar RNA, C/D box 32A | 10563114 | 27209 | 1.25 | 0.0071 | 0.0319 |
| Snord33 | small nucleolar RNA, C/D box 33 | 10563112 | 27208 | 1.25 | 0.0049 | 0.0830 |
| Snord87 | small nucleolar RNA, C/D box 87 | 10353034 | 266793 | 1.27 | 0.0085 | 0.0642 |
| Snord95 | small nucleolar RNA, C/D box 95 | 10375501 | 100216540 | 1.17 | 0.0041 | 0.0757 |
| Snora28 | small nucleolar RNA, H/ACA box 28 | 10398693 | 100316932 | 1.18 | 0.0023 | 0.1090 |
| Snora30 | small nucleolar RNA, H/ACA box 30 | 10557703 | 100217442 | 1.29 | 0.0058 | 0.0653 |
| Snora34 | small nucleolar RNA, H/ACA box 34 | 10432176 | 100217417 | 1.24 | 0.0074 | 0.1044 |
| Snora73a | small nucleolar RNA, H/ACA box 73a | 10516908 | 100306944 | 1.23 | 0.0021 | 0.2521 |
| Snora73b | small nucleolar RNA, H/ACA box 73b | 10516906 | 100306945 | 1.27 | 0.0028 | 0.1662 |
| Snora75 | small nucleolar RNA, H/ACA box 75 | 10356329 | 100303740 | 1.18 | 0.0058 | 0.0935 |
| Smo | smoothened homolog (Drosophila) | 10536917 | 319757 | 1.16 | 0.0040 | 0.1740 |
| Slc12a2 | solute carrier family 12, member 2 | 10455873 | 20496 | 1.22 | 0.0078 | 0.1457 |
| Slc12a4 | solute carrier family 12, member 4 | 10581395 | 20498 | 1.32 | 0.0206 | 0.0333 |
| Slc12a7 | solute carrier family 12, member 7 | 10406111 | 20499 | 1.28 | 0.0213 | 0.0287 |
| Slc13a1 | solute carrier family 13 (sodium/sulfate symporters), member 1 | 10543411 | 55961 | 1.18 | 0.0045 | 0.1352 |
| Slc13a4 | solute carrier family 13 (sodium/sulfate symporters), member 4 | 10543921 | 243755 | 1.77 | 0.0026 | 0.0422 |
| Slc16a12 | solute carrier family 16 (monocarboxylic acid transporters), member 12 | 10467153 | 240638 | 1.56 | 0.0113 | 0.0292 |
| Slc16a4 | solute carrier family 16 (monocarboxylic acid transporters), member 4 | 10495206 | 229699 | 1.43 | 0.0089 | 0.0422 |
| Slc16a6 | solute carrier family 16 (monocarboxylic acid transporters), member 6 | 10392440 | 104681 | 1.20 | 0.0134 | 0.1389 |
| Slc16a9 | solute carrier family 16 (monocarboxylic acid transporters), member 9 | 10363860 | 66859 | 1.43 | 0.0110 | 0.0510 |
| Slc19a1 | solute carrier family 19 (sodium/hydrogen exchanger), member 1 | 10364239 | 20509 | 1.29 | 0.0261 | 0.0085 |
| Slc2a12 | solute carrier family 2 (facilitated glucose transporter), member 12 | 10362104 | 353169 | 1.74 | 0.0022 | 0.0750 |
| Slc22a8 | solute carrier family 22 (organic anion transporter), member 8 | 10461115 | 19879 | 1.31 | 0.0140 | 0.0112 |
| Slc22a18 | solute carrier family 22 (organic cation transporter), member 18 | 10559297 | 18400 | 1.25 | 0.0092 | 0.0324 |
| Slc22a21 | solute carrier family 22 (organic cation transporter), member 21 | 10385883 | 56517 | 1.19 | 0.0063 | 0.0845 |
| Slc22a5 | solute carrier family 22 (organic cation transporter), member 5 | 10385872 | 20520 | 1.25 | 0.0169 | 0.0585 |
| Slc24a1 | solute carrier family 24 (sodium/potassium/calcium exchanger), member 1 | 10594490 | 214111 | 1.17 | 0.0082 | 0.0720 |
| Slc24a5 | solute carrier family 24, member 5 | 10475567 | 317750 | 1.36 | 0.0048 | 0.1027 |
| Slc25a40 | solute carrier family 25, member 40 | 10595570 | 319653 | 1.16 | 0.0120 | 0.0741 |
| Slc26a7 | solute carrier family 26, member 7 | 10511631 | 208890 | 1.35 | 0.0211 | 0.0105 |
| Slc28a3 | solute carrier family 28 (sodium-coupled nucleoside transporter), member 3 | 10409713 | 114304 | 1.68 | 0.0131 | 0.0279 |
| Slc29a4 | solute carrier family 29 (nucleoside transporters), member 4 | 10527148 | 243328 | 1.21 | 0.0013 | 0.2210 |
| Slc31a1 | solute carrier family 31, member 1 | 10505276 | 20529 | 1.44 | 0.0108 | 0.0738 |
| Slc37a2 | solute carrier family 37 (glycerol-3-phosphate transporter), member 2 | 10592266 | 56857 | 1.36 | 0.0128 | 0.0818 |
| Slc38a3 | solute carrier family 38, member 3 | 10596718 | 76257 | 1.22 | 0.0068 | 0.1619 |
| Slc39a4 | solute carrier family 39 (zinc transporter), member 4 | 10430006 | 72027 | 1.82 | 0.0103 | 0.0477 |
| Slc4a2 | solute carrier family 4 (anion exchanger), member 2 | 10520187 | 20535 | 1.57 | 0.0086 | 0.0404 |
| Slc4a5 | solute carrier family 4, sodium bicarbonate cotransporter, member 5 | 10539393 | 232156 | 2.17 | 0.0041 | 0.0488 |
| Slc5a6 | solute carrier family 5 (sodium-dependent vitamin transporter), member 6 | 10529052 | 330064 | 1.17 | 0.0130 | 0.1562 |
| Slc6a20a | solute carrier family 6 (neurotransmitter transporter), member 20A | 10597960 | 102680 | 1.26 | 0.0037 | 0.0940 |
| Slc7a10 | solute carrier family 7 (cationic amino acid transporter, y+ system), member 10 | 10552143 | 53896 | 1.25 | 0.0139 | 0.0654 |
| Slc7a11 | solute carrier family 7 (cationic amino acid transporter, y+ system), member 11 | 10498024 | 26570 | 1.15 | 0.0077 | 0.1653 |
| Slco1a4 | solute carrier organic anion transporter family, member 1a4 | 10548996 | 28250 | 1.36 | 0.0107 | 0.0052 |
| Slco1a5 | solute carrier organic anion transporter family, member 1a5 | 10549041 | 108096 | 1.20 | 0.0104 | 0.0367 |
| Slco1c1 | solute carrier organic anion transporter family, member 1c1 | 10542596 | 58807 | 1.25 | 0.0120 | 0.1190 |
| Sstr1 | somatostatin receptor 1 | 10395889 | 20605 | 1.17 | 0.0054 | 0.2462 |
| Sorbs3 | sorbin and SH3 domain containing 3 | 10421269 | 20410 | 1.21 | 0.0097 | 0.0964 |
| Smoc1 | SPARC related modular calcium binding 1 | 10396936 | 64075 | 1.21 | 0.0007 | 0.1039 |
| Spdya | speedy homolog A (Xenopus laevis) | 10446679 | 70891 | 1.15 | 0.0035 | 0.2430 |
| Spag16 | sperm associated antigen 16 | 10347163 | 66722 | 1.48 | 0.0010 | 0.0833 |
| Spag6 | sperm associated antigen 6 | 10438049 | 50525 | 1.21 | 0.0010 | 0.1376 |
| Spag8 | sperm associated antigen 8 | 10512598 | 433700 | 1.21 | 0.0068 | 0.0720 |
| Spef2 | sperm flagellar 2 | 10427693 | 320277 | 1.39 | 0.0106 | 0.0772 |
| Ssfa2 | sperm specific antigen 2 | 10473160 | 70599 | 1.18 | 0.0085 | 0.1312 |
| Spata17 | spermatogenesis associated 17 | 10360942 | 74717 | 1.26 | 0.0121 | 0.0517 |
| Spata6 | spermatogenesis associated 6 | 10507040 | 67946 | 1.30 | 0.0066 | 0.0854 |
| Smpdl3a | sphingomyelin phosphodiesterase, acid-like 3A | 10363231 | 57319 | 1.19 | 0.0111 | 0.1364 |
| Sgms2 | sphingomyelin synthase 2 | 10502224 | 74442 | 1.41 | 0.0068 | 0.1034 |
| S1pr3 | sphingosine-1-phosphate receptor 3 | 10405179 | 13610 | 1.16 | 0.0130 | 0.0787 |
| Sfrs17b | splicing factor, arginine/serine-rich 17b | 10604038 | 338351 | 1.18 | 0.0035 | 0.1377 |
| St6galnac2 | ST6 (alpha-N-acetyl-neuraminyl-2,3-beta-galactosyl-1,3)-N-acetylgalactosaminide alpha-2,6-sialyltransferase 2 | 10393166 | 20446 | 1.53 | 0.0086 | 0.0405 |
| Steap4 | STEAP family member 4 | 10519497 | 117167 | 1.44 | 0.0124 | 0.0275 |
| Stra6 | stimulated by retinoic acid gene 6 | 10585803 | 20897 | 1.28 | 0.0124 | 0.0971 |
| Stra8 | stimulated by retinoic acid gene 8 | 10537231 | 20899 | 1.19 | 0.0010 | 0.1852 |
| Sulf1 | sulfatase 1 | 10344897 | 240725 | 1.90 | 0.0051 | 0.0381 |
| Sult1c2 | sulfotransferase family, cytosolic, 1C, member 2 | 10451818 | 69083 | 1.71 | 0.0056 | 0.0477 |
| Sod3 | superoxide dismutase 3, extracellular | 10521824 | 20657 | 1.26 | 0.0112 | 0.0831 |
| Susd2 | sushi domain containing 2 | 10369932 | 71733 | 1.20 | 0.0027 | 0.2188 |
| Svep1 | sushi, von Willebrand factor type A, EGF and pentraxin domain containing 1 | 10513208 | 64817 | 1.18 | 0.0121 | 0.0110 |
| Syne2 | synaptic nuclear envelope 2 | 10396608 | 319565 | 1.29 | 0.0168 | 0.0482 |
| Syngr2 | synaptogyrin 2 | 10382980 | 20973 | 1.20 | 0.0092 | 0.1032 |
| Syt9 | synaptotagmin IX | 10556067 | 60510 | 1.29 | 0.0039 | 0.1293 |
| Syt15 | synaptotagmin XV | 10414081 | 319508 | 1.15 | 0.0051 | 0.0538 |
| Stxbp4 | syntaxin binding protein 4 | 10389797 | 20913 | 1.16 | 0.0039 | 0.1775 |
| Tgtp1 | T-cell specific GTPase 1 | 10385533 | 21822 | 1.22 | 0.0071 | 0.0806 |
| Tcirg1 | T-cell, immune regulator 1, ATPase, H+ transporting, lysosomal V0 protein A3 | 10464529 | 27060 | 1.18 | 0.0024 | 0.0927 |
| Tc2n | tandem C2 domains, nuclear | 10402195 | 74413 | 1.88 | 0.0027 | 0.0719 |
| Tapbp | TAP binding protein | 10444068 | 21356 | 1.20 | 0.0138 | 0.0642 |
| Tas2r130 | taste receptor, type 2, member 130 | 10548602 | 387355 | 1.17 | 0.0088 | 0.0432 |
| Taf1d | TATA box binding protein (Tbp)-associated factor, RNA polymerase I, D | 10583318 | 75316 | 1.16 | 0.0015 | 0.0496 |
| Tbc1d2 | TBC1 domain family, member 2 | 10512791 | 381605 | 1.25 | 0.0184 | 0.0418 |
| Tbc1d9 | TBC1 domain family, member 9 | 10573128 | 71310 | 1.19 | 0.0094 | 0.1604 |
| Tgds | TDP-glucose 4,6-dehydratase | 10422259 | 76355 | 1.15 | 0.0044 | 0.1618 |
| Tshz1 | teashirt zinc finger family member 1 | 10460057 | 110796 | 1.19 | 0.0069 | 0.1003 |
| Tekt1 | tektin 1 | 10388174 | 21689 | 1.16 | 0.0029 | 0.2471 |
| Tekt2 | tektin 2 | 10516335 | 24084 | 1.19 | 0.0069 | 0.0706 |
| Tenc1 | tensin like C1 domain-containing phosphatase | 10427095 | 209039 | 1.29 | 0.0238 | 0.0031 |
| Tex9 | testis expressed gene 9 | 10594911 | 21778 | 1.18 | 0.0056 | 0.0951 |
| Tsga14 | testis specific gene A14 | 10543725 | 83922 | 1.16 | 0.0113 | 0.1197 |
| Tsga8 | testis specific gene A8 | 10605568 | 60600 | 1.18 | 0.0116 | 0.0730 |
| Tspan33 | tetraspanin 33 | 10536908 | 232670 | 1.20 | 0.0020 | 0.1680 |
| Tspan4 | tetraspanin 4 | 10558961 | 64540 | 1.24 | 0.0116 | 0.0431 |
| Ttc18 | tetratricopeptide repeat domain 18 | 10417841 | 76670 | 1.30 | 0.0006 | 0.1101 |
| Ttc21a | tetratricopeptide repeat domain 21A | 10590212 | 74052 | 1.32 | 0.0096 | 0.1090 |
| Ttc25 | tetratricopeptide repeat domain 25 | 10381140 | 74407 | 1.32 | 0.0242 | 0.0157 |
| Tgif1 | TGFB-induced factor homeobox 1 | 10452633 | 21815 | 1.17 | 0.0227 | 0.0650 |
| Thbs1 | thrombospondin 1 | 10474700 | 21825 | 1.23 | 0.0126 | 0.0938 |
| Thbs2 | thrombospondin 2 | 10447951 | 21826 | 1.18 | 0.0037 | 0.1550 |
| Thsd1 | thrombospondin, type I, domain 1 | 10570786 | 56229 | 1.21 | 0.0079 | 0.0350 |
| Trip10 | thyroid hormone receptor interactor 10 | 10446235 | 106628 | 1.18 | 0.0151 | 0.0289 |
| Trh | thyrotropin releasing hormone | 10546417 | 22044 | 3.57 | 0.0020 | 0.0857 |
| Tjp3 | tight junction protein 3 | 10371111 | 27375 | 1.18 | 0.0220 | 0.0319 |
| Traf1 | TNF receptor-associated factor 1 | 10481949 | 22029 | 1.25 | 0.0115 | 0.0795 |
| Tlr4 | toll-like receptor 4 | 10505517 | 21898 | 1.17 | 0.0072 | 0.0662 |
| Tor3a | torsin family 3, member A | 10359181 | 30935 | 1.19 | 0.0057 | 0.1429 |
| Traf3ip1 | TRAF3 interacting protein 1 | 10348600 | 74019 | 1.22 | 0.0232 | 0.0261 |
| Sp3 | trans-acting transcription factor 3 | 10483646 | 20687 | 1.18 | 0.0005 | 0.1659 |
| Tcn2 | transcobalamin 2 | 10383799 | 21452 | 1.37 | 0.0029 | 0.1045 |
| Tcea3 | transcription elongation factor A (SII), 3 | 10509204 | 21401 | 1.21 | 0.0220 | 0.0040 |
| Trp53rk | transformation related protein 53 regulating kinase | 10478748 | 76367 | 1.18 | 0.0022 | 0.0949 |
| Trp73 | transformation related protein 73 | 10518989 | 22062 | 1.16 | 0.0023 | 0.1305 |
| Tgfbi | transforming growth factor, beta induced | 10405587 | 21810 | 1.16 | 0.0046 | 0.2836 |
| Tgfbr2 | transforming growth factor, beta receptor II | 10597518 | 21813 | 1.23 | 0.0189 | 0.0337 |
| Trpm3 | transient receptor potential cation channel, subfamily M, member 3 | 10462039 | 226025 | 1.51 | 0.0124 | 0.0411 |
| Trpv4 | transient receptor potential cation channel, subfamily V, member 4 | 10532839 | 63873 | 1.75 | 0.0020 | 0.0684 |
| Tmprss11a | transmembrane protease, serine 11a | 10530974 | 194597 | 1.87 | 0.0014 | 0.0644 |
| Tmprss5 | transmembrane protease, serine 5 (spinesin) | 10585146 | 80893 | 1.23 | 0.0033 | 0.1013 |
| Tmem107 | transmembrane protein 107 | 10377418 | 66910 | 1.20 | 0.0129 | 0.1185 |
| Tmem123 | transmembrane protein 123 | 10583145 | 71929 | 1.19 | 0.0052 | 0.1546 |
| Tmem146 | transmembrane protein 146 | 10446136 | 106757 | 1.24 | 0.0122 | 0.0631 |
| Tmem149 | transmembrane protein 149 | 10551989 | 101883 | 1.19 | 0.0022 | 0.0936 |
| Tmem176b | transmembrane protein 176B | 10544596 | 65963 | 1.16 | 0.0036 | 0.2350 |
| Tmem179b | transmembrane protein 179B | 10465783 | 67706 | 1.20 | 0.0136 | 0.0826 |
| Tmem184a | transmembrane protein 184a | 10535174 | 231832 | 1.60 | 0.0102 | 0.0492 |
| Tmem20 | transmembrane protein 20 | 10462918 | 240660 | 1.21 | 0.0094 | 0.0982 |
| Tmem72 | transmembrane protein 72 | 10547191 | 319776 | 2.04 | 0.0042 | 0.0403 |
| Tmem98 | transmembrane protein 98 | 10379489 | 103743 | 1.36 | 0.0058 | 0.0791 |
| Tchp | trichoplein, keratin filament binding | 10524572 | 77832 | 1.23 | 0.0219 | 0.0026 |
| Trim12 | tripartite motif-containing 12 | 10566326 | 76681 | 1.19 | 0.0046 | 0.1173 |
| Trim21 | tripartite motif-containing 21 | 10566144 | 20821 | 1.16 | 0.0075 | 0.1239 |
| Trim56 | tripartite motif-containing 56 | 10534679 | 384309 | 1.17 | 0.0082 | 0.1103 |
| Tmod4 | tropomodulin 4 | 10494160 | 50874 | 1.22 | 0.0015 | 0.0949 |
| Tnni1 | troponin I, skeletal, slow 1 | 10350149 | 21952 | 1.16 | 0.0032 | 0.1084 |
| Tnnt1 | troponin T1, skeletal, slow | 10559547 | 21955 | 1.16 | 0.0003 | 0.0289 |
| Ttll6 | tubulin tyrosine ligase-like family, member 6 | 10380599 | 237930 | 1.35 | 0.0252 | 0.0219 |
| Ttll8 | tubulin tyrosine ligase-like family, member 8 | 10431311 | 239591 | 1.22 | 0.0264 | 0.0210 |
| Ttll9 | tubulin tyrosine ligase-like family, member 9 | 10477218 | 74711 | 1.16 | 0.0104 | 0.0911 |
| Tubb6 | tubulin, beta 6 | 10456400 | 67951 | 1.22 | 0.0067 | 0.0722 |
| Tinagl1 | tubulointerstitial nephritis antigen-like 1 | 10516735 | 94242 | 1.32 | 0.0220 | 0.0372 |
| Tuft1 | tuftelin 1 | 10500011 | 22156 | 1.26 | 0.0038 | 0.0718 |
| Tnfsf10 | tumor necrosis factor (ligand) superfamily, member 10 | 10491091 | 22035 | 1.25 | 0.0091 | 0.0100 |
| Tnfrsf1a | tumor necrosis factor receptor superfamily, member 1a | 10541895 | 21937 | 1.16 | 0.0013 | 0.1442 |
| Tnfaip8 | tumor necrosis factor, alpha-induced protein 8 | 10455647 | 106869 | 1.26 | 0.0107 | 0.0900 |
| Ubc | ubiquitin C | 10525885 | 22190 | 1.20 | 0.0167 | 0.0346 |
| Ubxn11 | UBX domain protein 11 | 10508936 | 67586 | 1.26 | 0.0203 | 0.0266 |
| Ugt8a | UDP galactosyltransferase 8A | 10501963 | 22239 | 1.15 | 0.0034 | 0.2491 |
| Galnt4 | UDP-N-acetyl-alpha-D-galactosamine:polypeptide N-acetylgalactosaminyltransferase 4 | 10366038 | 14426 | 1.20 | 0.0094 | 0.0597 |
| Unc13c | unc-13 homolog C (C. elegans) | 10594969 | 208898 | 1.15 | 0.0020 | 0.1558 |
| Ucp2 | uncoupling protein 2 (mitochondrial, proton carrier) | 10555389 | 22228 | 1.33 | 0.0069 | 0.0914 |
| Ushbp1 | Usher syndrome 1C binding protein 1 | 10579486 | 234395 | 1.20 | 0.0031 | 0.0860 |
| Uaca | uveal autoantigen with coiled-coil domains and ankyrin repeats | 10586017 | 72565 | 1.19 | 0.0071 | 0.0493 |
| Vsig10 | V-set and immunoglobulin domain containing 10 | 10524878 | 231668 | 1.18 | 0.0112 | 0.0712 |
| Vtcn1 | V-set domain containing T cell activation inhibitor 1 | 10494761 | 242122 | 1.31 | 0.0141 | 0.0192 |
| Vash2 | vasohibin 2 | 10361055 | 226841 | 1.16 | 0.0139 | 0.1005 |
| Vasn | vasorin | 10433274 | 246154 | 1.19 | 0.0134 | 0.0855 |
| Vaultrc5 | vault RNA component 5 | 10455015 | 378472 | 1.23 | 0.0005 | 0.0950 |
| Vat1l | vesicle amine transport protein 1 homolog-like (T. californica) | 10575693 | 270097 | 1.34 | 0.0032 | 0.1137 |
| Vamp8 | vesicle-associated membrane protein 8 | 10545409 | 22320 | 1.33 | 0.0092 | 0.1076 |
| Vgll3 | vestigial like 3 (Drosophila) | 10436487 | 73569 | 1.45 | 0.0139 | 0.0206 |
| Vmn1r206 | vomeronasal 1 receptor 206 | 10408142 | 171250 | 1.18 | 0.0028 | 0.1505 |
| Vmn1r211 | vomeronasal 1 receptor 211 | 10408152 | 171277 | 1.27 | 0.0083 | 0.0681 |
| Vmn1r213 | vomeronasal 1 receptor 213 | 10404008 | 171249 | 1.18 | 0.0063 | 0.0945 |
| Vmn1r234 | vomeronasal 1 receptor 234 | 10442182 | 171232 | 1.26 | 0.0072 | 0.0895 |
| Vmn1r86 | vomeronasal 1 receptor 86 | 10560089 | 100312473 | 1.15 | 0.0009 | 0.2144 |
| Vmn1r-ps103 | vomeronasal 1 receptor, pseudogene 103 | 10403998 | 171245 | 1.24 | 0.0035 | 0.0854 |
| Vmn2r50 | vomeronasal 2, receptor 50 | 10549932 | 434117 | 1.20 | 0.0088 | 0.0962 |
| Vmn2r33 | vomeronasal 2, receptor33 | 10549929 | 624512 | 1.20 | 0.0021 | 0.0881 |
| Vwa1 | von Willebrand factor A domain containing 1 | 10519196 | 246228 | 1.21 | 0.0010 | 0.0587 |
| Vwa3a | von Willebrand factor A domain containing 3A | 10556962 | 233813 | 1.22 | 0.0042 | 0.1598 |
| Vwf | Von Willebrand factor homolog | 10541910 | 22371 | 1.30 | 0.0057 | 0.0223 |
| Wfdc13 | WAP four-disulfide core domain 13 | 10478544 | 408190 | 1.28 | 0.0002 | 0.0383 |
| Wfikkn2 | WAP, follistatin/kazal, immunoglobulin, kunitz and netrin domain containing 2 | 10389879 | 278507 | 1.57 | 0.0120 | 0.0166 |
| Wfikkn1 | WAP, FS, Ig, KU, and NTR-containing protein 1 | 10449148 | 215001 | 1.21 | 0.0003 | 0.1518 |
| Wdr25 | WD repeat domain 25 | 10398308 | 212198 | 1.17 | 0.0115 | 0.0799 |
| Wdr52 | WD repeat domain 52 | 10435862 | 212517 | 1.38 | 0.0047 | 0.1257 |
| Wdr63 | WD repeat domain 63 | 10502661 | 242253 | 1.49 | 0.0014 | 0.1062 |
| Wdr65 | WD repeat domain 65 | 10515803 | 68625 | 1.24 | 0.0095 | 0.0447 |
| Wdr72 | WD repeat domain 72 | 10587051 | 546144 | 1.26 | 0.0117 | 0.0203 |
| Wdr78 | WD repeat domain 78 | 10514713 | 242584 | 1.22 | 0.0104 | 0.1241 |
| Wdr86 | WD repeat domain 86 | 10528679 | 269633 | 1.42 | 0.0084 | 0.0053 |
| Wdr90 | WD repeat domain 90 | 10449111 | 106618 | 1.16 | 0.0003 | 0.1582 |
| Whrn | whirlin | 10513692 | 73750 | 1.15 | 0.0077 | 0.1504 |
| Wls | wntless homolog (Drosophila) | 10497149 | 68151 | 1.19 | 0.0079 | 0.1490 |
| Ybx2 | Y box protein 2 | 10377662 | 53422 | 1.16 | 0.0085 | 0.0950 |
| Zbtb34 | zinc finger and BTB domain containing 34 | 10481827 | 241311 | 1.17 | 0.0117 | 0.0784 |
| Zbtb46 | zinc finger and BTB domain containing 46 | 10490682 | 72147 | 1.21 | 0.0066 | 0.0460 |
| Zbtb7b | zinc finger and BTB domain containing 7B | 10499612 | 22724 | 1.16 | 0.0158 | 0.0997 |
| Zfp229 | zinc finger protein | 10442250 | 381067 | 1.22 | 0.0092 | 0.0922 |
| Zfp119 | zinc finger protein 119 | 10451879 | 104349 | 1.21 | 0.0132 | 0.0439 |
| Zfp167 | zinc finger protein 167 | 10590479 | 382118 | 1.33 | 0.0159 | 0.0052 |
| Zfp185 | zinc finger protein 185 | 10600093 | 22673 | 1.42 | 0.0085 | 0.0704 |
| Zfp36l1 | zinc finger protein 36, C3H type-like 1 | 10401238 | 12192 | 1.16 | 0.0030 | 0.1677 |
| Zfp455 | zinc finger protein 455 | 10405927 | 218311 | 1.15 | 0.0047 | 0.1900 |
| Zfp54 | zinc finger protein 54 | 10442199 | 22712 | 1.21 | 0.0142 | 0.0570 |
| Zfp568 | zinc finger protein 568 | 10551803 | 243905 | 1.19 | 0.0056 | 0.0709 |
| Zfp7 | zinc finger protein 7 | 10425012 | 223669 | 1.18 | 0.0040 | 0.1079 |
| Zfp768 | zinc finger protein 768 | 10568225 | 233890 | 1.18 | 0.0052 | 0.0411 |
| Zfp772 | zinc finger protein 772 | 10559919 | 232855 | 1.20 | 0.0107 | 0.0824 |
| Zfp825 | zinc finger protein 825 | 10358432 | 235956 | 1.15 | 0.0031 | 0.1626 |
| Zic5 | zinc finger protein of the cerebellum 5 | 10422504 | 65100 | 1.22 | 0.0100 | 0.0589 |
| Zmiz1 | zinc finger, MIZ-type containing 1 | 10413212 | 328365 | 1.33 | 0.0154 | 0.0007 |
| Zmynd12 | zinc finger, MYND domain containing 12 | 10507644 | 332934 | 1.17 | 0.0015 | 0.1977 |
| Zar1 | zygote arrest 1 | 10530496 | 317755 | 1.24 | 0.0100 | 0.0581 |
