# Supplementary material for: Chronic airway-induced allergy in mice modifies gene expression in the brain toward insulin resistance and inflammatory responses
Source: J Neuroinflammation. 2013 Aug 1;10:99. doi: 10.1186/1742-2094-10-99 (PMC3750454; doi:10.1186/1742-2094-10-99)
Supplement: Additional file 2: Table 3 — Genes involved in enrichment of KEGGa pathways in the hippocampus. Significant differentially expressed genes (DEGs) between allergic mice compared to controls were detected by orthogonal projection to latent structures discriminant analysis (OPLS-DA) and subsequently subjected to pathway analysis. Genes that were involved in significantly enriching the indicated KEGG pathways in the hippocampus are shown in the list. aMAPK, mitogen activated protein kinase. Table 4. Genes involved in enrichment of KEGGa pathways in the frontal cortex. Significant differentially expressed genes (DEGs) between allergic mice compared to controls were detected by orthogonal projection to latent structures discriminant analysis (OPLS-DA) and subsequently subjected to pathway analysis. Genes that were involved in significantly enriching the indicated KEGG pathways in the frontal cortex are shown in the list. aKyoto Encyclopedia of Genes and Genomes. [file 1742-2094-10-99-S2.docx]

Table 3 Genes involved in enrichment of KEGG^a^ pathways in the hippocampus. Significant differentially expressed genes (DEGs) between allergic mice compared to controls were detected by orthogonal projection to latent structures discriminant analysis (OPLS-DA) and subsequently subjected to pathway analysis. Genes that were involved in significantly enriching each KEGG pathway in the hippocampus are depicted in the list.

| **MAPK signaling pathway (04010)** | | | | |
| --- | --- | --- | --- | --- |
| **Gene symbol** | **Gene name** | **Fold change** | **Entrez ID** | **Ensemble ID** |
| *Pdgfb* | Platelet-derived growth factor, B polypeptide | −1.12 | 18591 | ENSMUSG00000000489 |
| *Gadd45b* | Growth arrest and DNAdamage-inducible 45β | −1.19 | 17873 | ENSMUSG00000015312 |
| *Il1r1* | Interleukin 1 receptor, type I | −1.20 | 16177 | ENSMUSG00000026072 |
| *Dusp4* | Dual specificity phosphatase 4 | −1.20 | 319520 | ENSMUSG00000031530 |
| *Map3k1* | Mitogen-activated protein kinase kinasekinase 1 | 1.22 | 26401 | ENSMUSG00000021754 |
| *Bdnf* | Brain-derived neurotrophic factor | −1.19 | 12064 | ENSMUSG00000048482 |
| *Fgf5* | Fibroblast growth factor 5 | −1.11 | 14176 | ENSMUSG00000029337 |
| *Cacna1d* | Calcium channel, voltage-dependent, L-type, α1D subunit | 1.12 | 12289 | ENSMUSG00000015968 |
| *Map2k2* | Mitogen-activated protein kinase kinase 2 | −1.11 | 26396 | ENSMUSG00000035027 |
| *Hspb1* | Heat shock protein 1 | −1.17 | 15507 | ENSMUSG00000004951 |
| *Map3k5* | Mitogen-activated protein kinase kinasekinase 5 | −1.12 | 26408 | ENSMUSG00000071369 |
| **Antigen processing and presentation (04612)** | | | | |
| *H2-Ab1* | Histocompatibility 2, class II antigen A, β1 | −1.53 | 14961 | ENSMUSG00000073421 |
| *Psme2* | Proteasome (prosome, macropain) 28 subunit, β | −1.23 | 19188 | ENSMUSG00000079197 |
| *Hspa5* | Heat shock protein 5 | −1.17 | 14828 | ENSMUSG00000026864 |
| *Cd74* | CD74 antigen (invariant polypeptide of major histocompatibility complex, class II antigen-associated) | −1.32 | 16149 | ENSMUSG00000024610 |
| *Ifna9* | Interferon α9 | 1.18 | 15972 | ENSMUSG00000078644 |
| *H2-Eb1* | Histocompatibility 2, class II antigen E β | −1.18 | 14969 | ENSMUSG00000060586 |
| *H2-Aa* | Histocompatibility 2, class II antigen Aα | −1.37 | 14960 | ENSMUSG00000036594 |
| **Cytokine–cytokine receptor interaction (04060)** | | | | |
| *Tnfrsf13c* | Tumor necrosis factor receptor superfamily, member 13c | 1.16 | 72049 | ENSMUSG00000068105 |
| *Pdgfb* | Platelet-derived growth factor, B polypeptide | −1.12 | 18591 | ENSMUSG00000000489 |
| *Ccl9* | Chemokine (C-C motif) ligand 9 | −1.16 | 20308 | ENSMUSG00000019122 |
| *Il1r1* | Interleukin 1 receptor, type I | −1.20 | 16177 | ENSMUSG00000026072 |
| *Ifna9* | Interferon α9 | 1.18 | 15972 | ENSMUSG00000078644 |
| *Pf4* | Platelet factor 4 | −1.15 | 56744 | ENSMUSG00000029373 |
| *Il17ra* | Interleukin 17 receptor A | −1.13 | 16172 | ENSMUSG00000002897 |
| *Il18rap* | Interleukin 18 receptor accessory protein | 1.26 | 16174 | ENSMUSG00000026068 |
| **Neurotrophin signaling pathway (04722)** | | | | |
| *Map2k2* | Mitogen-activated protein kinase kinase 2 | −1.11 | 26396 | ENSMUSG00000035027 |
| *Map3k1* | Mitogen-activated protein kinase kinasekinase 1 | 1.22 | 26401 | ENSMUSG00000021754 |
| *Pik3cg* | Phosphoinositide3-kinase, catalytic, γ polypeptide | −1.12 | 30955 | ENSMUSG00000020573 |
| *Map3k5* | Mitogen-activated protein kinase kinasekinase 5 | −1.12 | 26408 | ENSMUSG00000071369 |
| *Bdnf* | Brain-derived neurotrophic factor | −1.19 | 12064 | ENSMUSG00000048482 |
| **Toll-like receptor signaling pathway (04620)** | | | | |
| *Map2k2* | Mitogen-activated protein kinase kinase 2 | −1.11 | 26396 | ENSMUSG00000035027 |
| *Ifna9* | Interferon α9 | 1.18 | 15972 | ENSMUSG00000078644 |
| *Pik3cg* | Phosphoinositide3-kinase, catalytic, γ polypeptide | −1.12 | 30955 | ENSMUSG00000020573 |
| **Leukocyte transendothelial migration (04670)** | | | | |
| *Ezr* | Ezrin | −1.11 | 22350 | ENSMUSG00000052397 |
| *Actn3* | Actininα 3 | −1.15 | 11474 | ENSMUSG00000006457 |
| *Pik3cg* | Phosphoinositide3-kinase, catalytic, γ polypeptide | −1.12 | 30955 | ENSMUSG00000020573 |

^a^KEGG, Kyoto Encyclopedia of Genes and Genomes; MAPK, mitogen-activated protein kinase

Table 4 Genes involved in enrichment of KEGG^a^ pathways in the frontal cortex. Significant differentially expressed genes (DEGs) between allergic mice compared to controls were detected by orthogonal projection to latent structures discriminant analysis (OPLS-DA) and subsequently subjected to pathway analysis. Genes that were involved in significantly enriching each KEGG pathway in the frontal cortex are depicted in the list

| **Complement and coagulation cascades (04610)** | | | | |
| --- | --- | --- | --- | --- |
| **Gene symbol** | **Gene name** | **Fold change** | **Entrez ID** | **Ensemble ID** |
| *A2m* | α-2-macroglobulin | 1.69 | 232345 | ENSMUSG00000030111 |
| *C4b* | Complement component 4B (Childo blood group) | 1.26 | 12268 | ENSMUSG00000073418 |
| *Bdkrb1* | Bradykininreceptor, β1 | 1.20 | 12061 | ENSMUSG00000041347 |
| *Cd55* | CD55 antigen | 1.31 | 13136 | ENSMUSG00000026399 |
| *Cd59b* | CD59b antigen | 1.27 | 333883 | ENSMUSG00000068686 |
| *Cfh* | Complement component factor h | 1.21 | 12628 | ENSMUSG00000026365 |
| *F7* | Coagulation factor VII | 1.22 | 14068 | ENSMUSG00000031443 |
| *Cd59a* | CD59a antigen | 1.54 | 12509 | ENSMUSG00000032679 |
| *Vwf* | vonWillebrand factor homolog | 1.30 | 22371 | ENSMUSG00000001930 |
| **Hematopoietic cell lineage (04640)** | | | | |
| *Itga1* | Integrin α1 | 1.19 | 109700 | ENSMUSG00000042284 |
| *Itga5* | Integrin α5 (fibronectin receptor α) | 1.24 | 16402 | ENSMUSG00000000555 |
| *Cd24a* | CD24a antigen | 1.22 | 12484 | ENSMUSG00000047139 |
| *Cd9* | CD9 antigen | 1.17 | 12527 | ENSMUSG00000030342 |
| *Cd59a* | CD59a antigen | 1.54 | 12509 | ENSMUSG00000032679 |
| *Cd55* | CD55 antigen | 1.31 | 13136 | ENSMUSG00000026399 |
| *Cd59b* | CD59b antigen | 1.27 | 333883 | ENSMUSG00000068686 |
| *Anpep* | Alanyl(membrane) aminopeptidase | 1.20 | 16790 | ENSMUSG00000039062 |
| **Cytokine-cytokine receptor interaction (04060)** | | | | |
| *Il15* | Interleukin 15 | 1.17 | 16168 | ENSMUSG00000031712 |
| *Il12a* | Interleukin 12a | 1.23 | 16159 | ENSMUSG00000027776 |
| *Lepr* | Leptinreceptor | 1.45 | 16847 | ENSMUSG00000057722 |
| *Tnfrsf1a* | Tumor necrosis factor receptor superfamily, member 1a | 1.16 | 21937 | ENSMUSG00000030341 |
| *Il15ra* | Interleukin 15 receptor, α chain | 1.15 | 16169 | ENSMUSG00000023206 |
| *Bmp7* | Bone morphogenetic protein 7 | 1.47 | 12162 | ENSMUSG00000008999 |
| *Ccl25* | Chemokine (C-C motif) ligand 25 | 1.18 | 20300 | ENSMUSG00000023235 |
| *Ifna12* | Interferon α12 | 1.25 | 242519 | ENSMUSG00000073811 |
| *Kdr* | Kinase insert domain protein receptor | 1.26 | 16542 | ENSMUSG00000062960 |
| *Tnfsf10* | Tumor necrosis factor (ligand) superfamily, member 10 | 1.25 | 22035 | ENSMUSG00000039304 |
| *Tgfbr2* | Transforming growth factor, β receptor II | 1.23 | 21813 | ENSMUSG00000032440 |
| *Pdgfd* | Platelet-derived growth factor, D polypeptide | 1.32 | 71785 | ENSMUSG00000032006 |
| *Il10rb* | Interleukin 10 receptor, β | 1.23 | 16155 | ENSMUSG00000022969 |
| **Cell adhesion molecules (CAMs) (04514)** | | | | |
| *F11r* | F11 receptor | 1.36 | 16456 | ENSMUSG00000038235 |
| *H2-Q7* | Histocompatibility 2, Q region locus 7 | 1.23 | 15018 | ENSMUSG00000060550 |
| *Cldn2* | Claudin2 | 1.98 | 12738 | ENSMUSG00000047230 |
| *Cdh1* | Cadherin 1 | 1.20 | 12550 | ENSMUSG00000000303 |
| *Cd6* | CD6 antigen | 1.17 | 12511 | ENSMUSG00000024670 |
| *Cd274* | CD274 antigen | 1.18 | 60533 | ENSMUSG00000016496 |
| *Ptprf* | Protein tyrosine phosphatase, receptor type, F | 1.16 | 19268 | ENSMUSG00000033295 |
| *Cdh3* | Cadherin 3 | 1.39 | 12560 | ENSMUSG00000061048 |
| *Mpz* | Myelin protein zero | 1.18 | 17528 | ENSMUSG00000056569 |
| *Pvrl2* | Poliovirus receptor-related 2 | 1.17 | 19294 | ENSMUSG00000062300 |
| **JAK-STAT signaling pathway (04630)** | | | | |
| *Il15* | Interleukin 15 | 1.17 | 16168 | ENSMUSG00000031712 |
| *Il12a* | Interleukin 12a | 1.23 | 16159 | ENSMUSG00000027776 |
| *Ifna12* | Interferon α12 | 1.25 | 242519 | ENSMUSG00000073811 |
| *Lepr* | Leptinreceptor | 1.45 | 16847 | ENSMUSG00000057722 |
| *Stat5a* | Signal transducer and activator of transcription 5A | 1.15 | 20850 | ENSMUSG00000004043 |
| *Il15ra* | Interleukin 15 receptor, α chain | 1.15 | 16169 | ENSMUSG00000023206 |
| *Il10rb* | Interleukin 10 receptor, β | 1.23 | 16155 | ENSMUSG00000022969 |
| *Cish* | Cytokine inducible SH2-containing protein | 1.23 | 12700 | ENSMUSG00000032578 |
| **Toll-like receptor signaling pathway (04620)** | | | | |
| *Casp8* | Caspase8 | 1.30 | 12370 | ENSMUSG00000026029 |
| *Il12a* | Interleukin 12a | 1.23 | 16159 | ENSMUSG00000027776 |
| *Tlr4* | Toll-like receptor 4 | 1.17 | 21898 | ENSMUSG00000039005 |
| *Ifna12* | Interferon α 12 | 1.25 | 242519 | ENSMUSG00000073811 |
| *Lbp* | Lipopolysaccharide-binding protein | 1.72 | 16803 | ENSMUSG00000016024 |

^a^KEGG, Kyoto Encyclopedia of Genes and Genomes.
